# Synthesis and Biological Evaluation of a Fused Structure of Indolizine and Pyrrolo[1,2-*c*]pyrimidine: Identification of Its Potent Anticancer Activity against Liver Cancer Cells

**DOI:** 10.3390/ph15111395

**Published:** 2022-11-12

**Authors:** Seonghyeon Nam, Yechan Lee, So-Hyeon Park, Wan Namkung, Ikyon Kim

**Affiliations:** College of Pharmacy and Yonsei Institute of Pharmaceutical Sciences, Yonsei University, 85 Songdogwahak-ro, Yeonsu-gu, Incheon 21983, Korea

**Keywords:** chemical space, indolizine, pyrrolo[1,2-*c*]pyrimidine, polyheterocycles, multi-component reaction, oxidative cyclization, liver cancer, anticancer activity, domino reaction, diversity-oriented synthesis

## Abstract

A highly efficient approach to a new indolizine scaffold fused with pyrrolo[1,2-*c*]pyrimidine was achieved via one-pot three-component coupling followed by an oxidative cyclization reaction. The simple two-step sequence allowed rapid access to various tetracyclic compounds from commercially available starting materials with the formation of five new bonds. Here, we observed the effects of these compounds on cell viability in HepG2, H1299, HT29, AGS, and A549 cancer cell lines. Interestingly, this fused scaffold had more potent anticancer activity in hepatocellular carcinoma HepG2 and Huh7 cells than other cancer cells. In particular, **5r** strongly decreased cell viability in HepG2 and Huh7 cells with an IC_50_ value of 0.22 ± 0.08 and 0.10 ± 0.11 µM, respectively, but had a very weak inhibitory effect on the cell viability of other cancer cell lines. In addition, **5r** significantly inhibited cell migration and induced apoptosis in HepG2 and Huh7 cells via the activation of caspase-3 and cleavage of PARP in a dose-dependent manner. Notably, the co-treatment of **5r** with gemcitabine resulted in the significant additional inhibition of cell viability in HepG2 and Huh7 cells. Our results suggest that **5r** could be used to develop new chemotype anticancer agents against liver cancers.

## 1. Introduction

**Figure 1:**
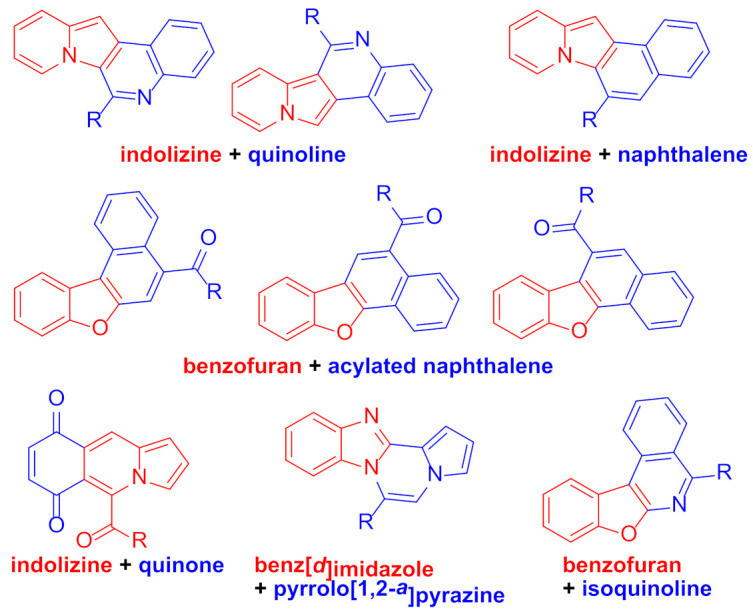
Some Fused Heterocyclic Systems Developed by Us.

Polyheterocycles are widely found in both natural products and artificial materials while exhibiting a wide range of biological and optophysical properties. Owing to their versatile usages in many different fields, efforts to expand the polyheterocyclic chemical space via design and synthesis are highly valuable [1,2]. As part of our research interest on novel polyheterocyclic structures, we designed several new heterocyclic scaffolds having two or more pharmacophores, hoping that these new fused polycycles might have intriguing properties that had not been previously discovered with each heterocyclic ring system. Along this line, we developed several efficient synthetic strategies of novel fused heterocycles [3,4,5], which enabled us to investigate their biological and/or photophysical activities (Figure 1) [6].

Liver cancer is one of the most common causes of cancer death worldwide and the fifth most common cancer in the United States [7]. Based on the announcement of Globocan 2020, 905,677 new liver cancer patients occurred in 2020 and 830,180 deaths were confirmed. The incidence of liver cancer is generally higher in developing countries and risk factors include hepatitis B virus, hepatitis C virus, fatty liver disease, smoking, alcoholic cirrhosis, obesity, and diabetes [8,9]. With respect to the surgical treatment of liver cancer, only 5 to 15% of patients in the early stage without cirrhosis can undergo surgery. In addition, commonly used drugs develop resistance within six months and long-term use causes toxicity problems and reduced drug effectiveness [10]. Despite advances in new therapeutic techniques for screening, prevention, diagnosis, and treatment, the mortality rate of the patients with liver cancer continues to rise [11]. Therefore, there is an urgent need for the development of new anticancer agents to overcome many limitations in the treatment of liver cancer. In this study, we generated a new polycyclic heteroaromatic scaffold consisting of indolizine and pyrrolo[1,2-*c*]pyrimidine and examined its anticancer effect on five different cancer cells.

**Scheme 1:**
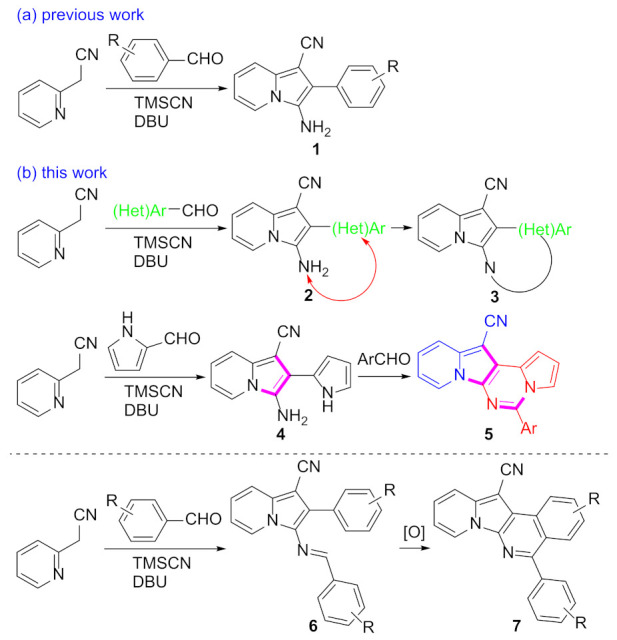
Synthetic Plan.

In 2017, Hulme and co-workers reported a direct one-pot three-component assembly route to 1-cyano-3-amino-2-arylindolizines **1** (Scheme 1a) [12]. Inspired by this work, we came up with the general idea that a new indolizine-fused heteroaromatic system **3** would be constructed via **2** by using a (hetero)aryl aldehyde in the three-component coupling process followed by oxidative cyclization (Scheme 1b). In this context, we planned to employ pyrrole-2-carboxaldehyde in the first step to gain access to **4**. The subsequent oxidative cyclization of **4** with an aldehyde leads to an indolizine skeleton fused with pyrrolo[1,2-*c*]pyrimidine **5**. As indolizine [13] and pyrrolo[1,2-*a*]pyrimidine [14,15] have been frequently employed in material and medicinal sciences, we anticipated to discover new biochemical functions with this new skeleton. Here, we wish to describe the synthesis and biological evaluation of this tetracyclic fused scaffold. While we were preparing this manuscript, the Hulme group disclosed the synthesis and photophysical properties of indolizino[3,2-*c*]isoquinoline **7** via the intermediate **6** [16].

## 2. Results and Discussion

### 2.1. Synthesis and Cell Inhibitory Activity of Pyrrolo[1′,2′:1,6]pyrimido[5,4-b]indolizines

**Scheme 2:**
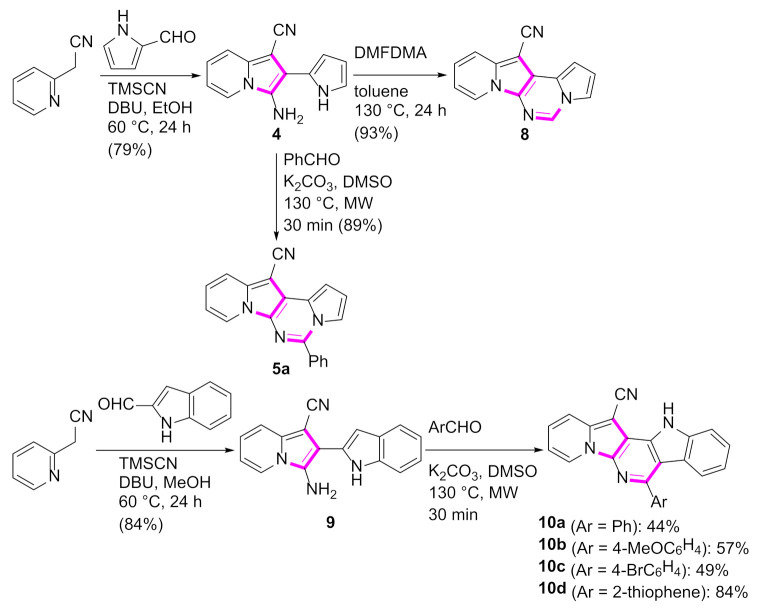
Synthesis of **5a**, **8**, and **10**.

By following Hulme’s procedure, we were able to make **4** from the reaction of pyridine-2-acetonitrile with pyrrole-2-carboxaldehyde and TMSCN in the presence of DBU at 60 °C in 79% yield (Scheme 2). The reaction of **4** with DMFDMA in toluene went smoothly to yield the basic tetracyclic skeleton **8**. The oxidative cyclization for the synthesis of the pyrimidine ring in **5** was realized by the treatment of **4** with benzaldehyde in the presence of K_2_CO_3_ in DMSO under microwave heating at 130 °C for 30 min to afford **5a** in 89% yield. It should be mentioned that this new tetracyclic heteroaromatic structure was constructed in two steps with the formation of five new bonds, illustrating the efficiency of our method. Interestingly, when **9** was allowed to react with aldehydes under similar conditions, pentacyclic indolizine–γ-carboline hybrids **10a–d** were obtained as a result of the involvement of the C3 carbon of the indole in the oxidative cyclization instead of the nitrogen of the indole to form the pyridine moiety [17,18].

Under the optimal conditions, various aldehydes were allowed to react with **4** to furnish derivatives **5** (Table 1). Electron-poor as well as electron-rich aryl moieties were installed at the C5 position of this heterocyclic system in good yields. Indolizine–pyrrolo[1,2-*c*]pyrimidine hybrids bearing heteroaromatic rings such as pyridine, indole, furan, and thiophene at the C5 site were readily accessed as well.

The preliminary biological screening of these compounds revealed that this class of compounds exhibited strong inhibitory activity against HepG2 and H1299 cells out of the five cancer cells evaluated (Table 2). In particular, stronger anticancer activities were observed with compounds **5k**, **5l**, and **5n** in HepG2 cells, having pyridine, indole, and thiophene at the C5 site of this scaffold.

Thus, more derivatives were prepared in a similar way and their anticancer activities against HepG2 and Huh7 cells were evaluated (Table 3). In general, heterocycles or aryl groups with ortho- or meta-substituent(s) at the C5 position seemed beneficial for enhanced anticancer activity. We were pleased to find that **5r** bearing a 2-hydroxyphenyl at the C5 site had more potency than **5n**. Interestingly, **5s** with a 3-hydroxyphenyl moiety at the C5 position exhibited less cytotoxicity than **5r**, indicating that the orientation of the hydroxyl in this scaffold is critical for potent activity.

More analogs of **5r** were synthesized to examine the effects of the substituent(s) of the 2-hydroxyphenyl moiety on inhibitory activity (Table 4). Unfortunately, an additional methoxyl or chlorine on the ring did not increase the potency.

### 2.2. Effect of **5r** on Cell Viability in HepG2, Huh7, AGS, H1299, A549, HaCaT, NIH3T3, and HEK293T Cells

**Figure 2:**
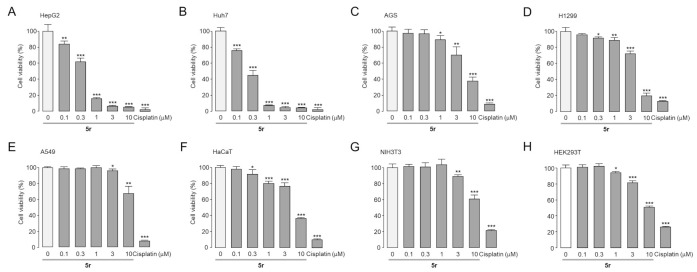
Effect of **5r** on cell viability in HepG2, Huh7, AGS, H1299, A549, HaCaT, NIH3T3, and HEK293T cells. (**A**–**H**) HepG2, Huh7, AGS, H1299, A549, HaCaT, NIH3T3, and HEK293T cells were cultured with **5r** at the indicated concentrations for 72 h, and medium was replaced every 24 h with freshly added **5r**. Cell viability was measured by MTS analysis (mean ± S.D., *n* = 5). * *p* < 0.05, ** *p* < 0.01, *** *p* < 0.001.

To further investigate the concentration-dependent effect of the most potent compound (**5r**) on cell viability in other cancer cell lines, we observed the cytotoxic effect of **5r** in HepG2, Huh7 hepatocellular carcinoma, AGS gastric adenocarcinoma, H1299 non-small cell lung carcinoma, and A549 lung carcinoma cell lines. As shown in Figure 2A,B, **5r** potently reduced cell viability in HepG2 and Huh7 cells with an IC_50_ value of 0.22 ± 0.08 and 0.10 ± 0.11 µM, respectively. However, **5r** had a weak effect on the other cancer cell lines, AGS, H1299, and A549 (Figure 2C–E). In addition, **5r** also weakly decreased cell viability in non-cancerous cells including HaCaT, NIH3T3, and HEK293T cells (Figure 2F–H). These results suggest that **5r** can be developed as a selective anticancer agent against liver cancer.

### 2.3. Inhibitory Effect of **5r** on Cell Migration in HepG2 and Huh7 Cells

**Figure 3:**
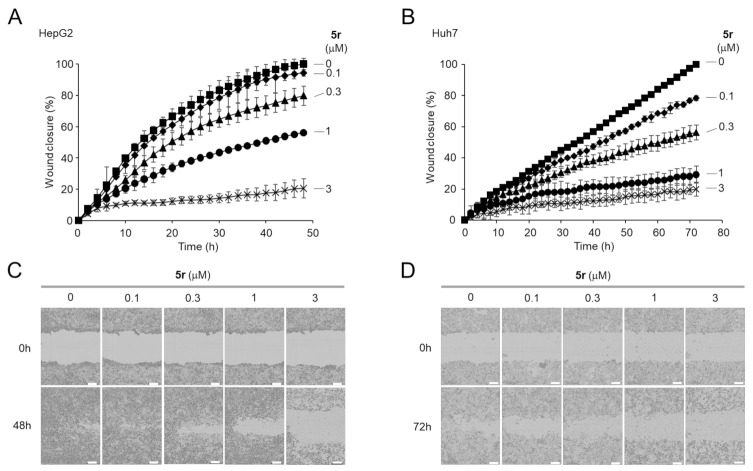
Inhibitory effect of **5r** on cell migration in HepG2 and Huh7 cells. (**A**,**B**) In vitro wound-healing assay was performed in HepG2 and Huh7 cells (mean ± S.D., *n* = 3). HepG2 and Huh7 cells were treated with the indicated concentrations of **5r**, and time-lapse images were acquired every 2 h after wound infliction. (**C**,**D**) Representative images were taken at 0, 48, or 72 h following administration of **5r** at the indicated concentrations.

To investigate the inhibitory effect of **5r** on cell migration, we performed an in vitro wound-healing assay in HepG2 and Huh7 cells. As shown in Figure 3, **5r** significantly inhibited cell migration in a dose-dependent manner in both HepG2 and Huh7 cells.

### 2.4. **5r** Induces Activation of Caspase-3 and PARP Cleavage in HepG2 and Huh7 Cells

**Figure 4:**
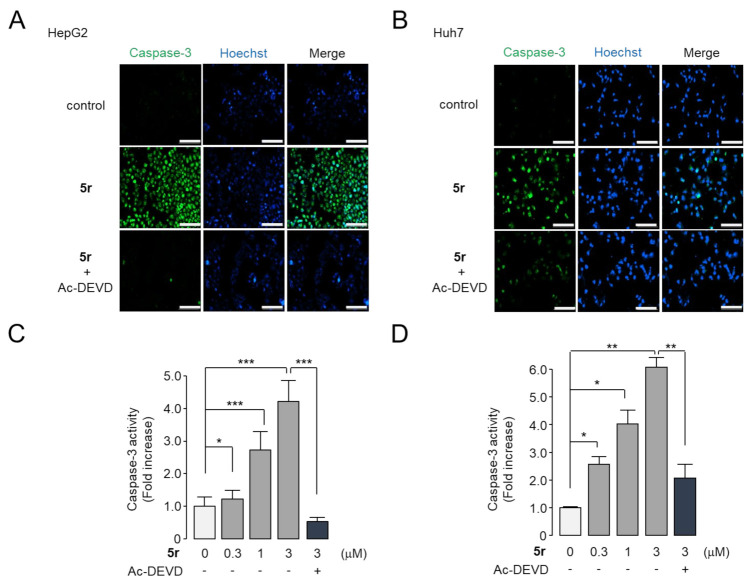

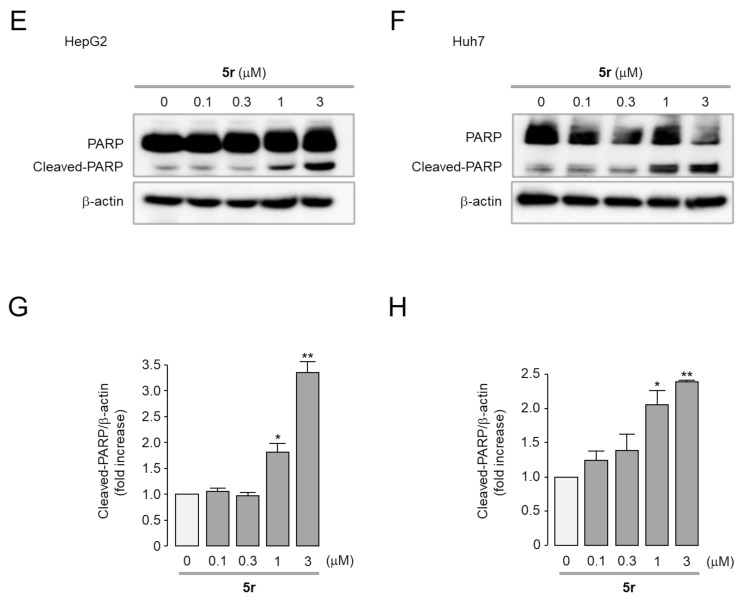
Effect of **5r** on activity of caspase-3 and cleavage of PARP in HepG2 and Huh7 cells. (**A**,**B**) HepG2 and Huh7 cells were incubated with 3 µM **5r** for 24 h and then incubated with 1 µM caspase-3 substrate (green) and 1 µM Hoechst 33,342 (blue) for 20 min before image acquisition. (**C**,**D**) Cells were cultured with **5r** at the indicated concentrations for 24 h and then 1 μM caspase-3 substrate was treated for 20 min. Caspase-3 was inhibited by 20 μM Ac-DEVD-CHO (mean ± S.D., n = 3). (**E**,**F**) Cells were cultured with **5r** at the indicated concentrations for 24 h, and expression level of PARP, cleaved-PARP and β-actin were measured by Western blot analysis. (**G**,**H**) Cleaved PARP protein intensities were normalized to β-actin (mean ± S.E., *n* = 3). * *p* < 0.05, ** *p* < 0.01, *** *p* < 0.001.

To investigate whether **5r** induces apoptosis in HepG2 and Huh7 liver cancer cells, we observed caspase-3 activation and the cleavage of PARP, a hallmark of apoptosis, in HepG2 and Huh7 cells. Interestingly, **5r** potently increased caspase-3 activity (green) in both HepG2 and Huh7 cells (Figure 4A,B). In addition, caspase-3 activity was significantly increased by **5r** in a dose-dependent manner, and the increased activity of caspase-3 was fully inhibited by Ac-DEVD-CHO, a specific caspase-3 inhibitor (Figure 4C,D). In the case of PARP cleavage, **5r** increased the expression level of cleaved PARP in a dose-dependent manner in HepG2 and Huh7 cells (Figure 4E–H). These results suggest that **5r** showed a significant anticancer effect by inducing apoptosis via the activation of caspase-3 and cleavage of PARP in HepG2 and Huh7 cells.

### 2.5. **5r** Dose Not Significantly Affect Cell Cycle of HepG2 Cells

**Figure 5:**
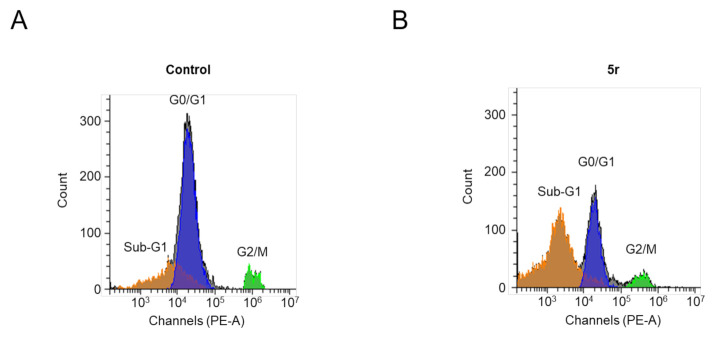
Effect of **5r** on cell cycle in HepG2 cells. (**A**,**B**) HepG2 cells were treated with 3 µM **5r** for 24 h and then cell cycle phases were measured by propidium iodide staining, followed by flow cytometry analysis.

To determine the effect of **5r** on the cell cycle of HepG2 cells, we performed a flow cytometry analysis using propidium iodide staining. As shown in Figure 5, **5r** significantly increased the ratios in the Sub-G1 (apoptotic peak) phase but did not affect the ratio in the G2/M phase in HepG2 cells. In the case of **5r**, the G0/G1 phase decreased from 64.6% to 29.3% and the Sub-G1 phase increased from 24.1% to 56.9% compared with the control. These results suggest that **5r** strongly increases apoptosis without a significant effect on cell cycle arrest.

### 2.6. Combination Effect of **5r** with Gemcitabine on Cell Viability in HepG2 and Huh7 Cells

**Figure 6:**
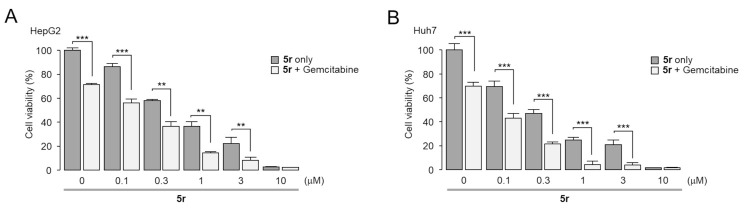
Combination effect of **5r** and gemcitabine on cell viability in HepG2 and Huh7 cells. (**A**,**B**) HepG2 and Huh7 cells were cultured with the indicated concentrations of **5r** and gemcitabine (0.3 μM) for 72 h, and medium was replaced every 24 h with freshly added **5r** and gemcitabine. Cell viability was determined by MTS analysis (mean ± S.D., *n* = 5). ** *p* < 0.01, *** *p* < 0.001.

Gemcitabine exhibits broad activity in a variety of solid tumors and has shown potent activity against hepatocellular carcinoma cells in preclinical studies [19,20]. However, gemcitabine alone showed a response rate of 0–20% in phase 2 clinical trials [21,22,23,24,25,26]. To investigate the combinational effect of **5r** with gemcitabine, a cell viability analysis was performed in HepG2 and Huh7 cells. As shown in Figure 6, interestingly, the co-treatment of **5r** with gemcitabine showed an additional effect rather than a synergistic effect. These results suggest that **5r**-based drug development could potentially increase the therapeutic effect in liver cancer.

## 3. Materials and Methods

### 3.1. General Methods

Unless specified, all reagents and starting materials were purchased from commercial sources and used as received without purification. “Concentrated” refers to the removal of volatile solvents via distillation using a rotary evaporator. “Dried” refers to pouring onto, or passing through, anhydrous magnesium sulfate followed by filtration. Flash chromatography was performed using silica gel (230−400 mesh) with hexane, ethyl acetate, and dichloromethane as the eluents. All reactions were monitored by thin-layer chromatography on 0.25 mm silica plates (F-254) visualized with UV light. Melting points were measured using a capillary melting point apparatus. Then, ^1^H and ^13^C NMR spectra were recorded on a 400 MHz NMR spectrometer and were described in terms of chemical shifts, multiplicity (s, singlet; d, doublet; t, triplet; q, quartet; m, multiplet), coupling constant in hertz (Hz), and number of protons (see Supplementary Materials). HRMS was measured with an electrospray ionization (ESI) and Q-TOF mass analyzer.

#### 3.1.1. Synthesis of **4**

To a mixture of 2-pyridylacetonitrile (472.1 μL, 4.23 mmol, 1.0 equiv) and pyrrole-2-carboxaldehyde (443.2 mg, 4.66 mmol, 1.1 equiv) in EtOH (10 mL) were added TMSCN (794.4 μL, 6.35 mmol, 1.5 equiv) and DBU (631.3 μL, 4.23 mmol, 1.0 equiv) at rt. After being stirred at 60 °C for 24 h, the reaction mixture was suction-filtered to give the desired product **4**. The filtrate was further purified by silica gel column chromatography (hexane:EtOAc:dichloromethane = 5:1:2) to give **4**. Overall, **4** was obtained in 79% yield (742.7 mg) as a brown solid.

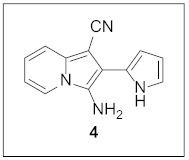
**3-Amino-2-(1*H*-pyrrol-2-yl)indolizine-1-carbonitrile (4).** Brown solid, m.p.: 192.7–193.6 °C (742.7 mg, 79%); **^1^H NMR** (400 MHz, DMSO-*d*_6_) δ 10.96 (s, 1H), 8.23 (d, *J* = 7.0 Hz, 1H), 7.48 (d, *J* = 8.8 Hz, 1H), 7.04–6.97 (m, 1H), 6.93–6.89 (m, 1H), 6.87 (t, *J* = 6.8 Hz, 1H), 6.48 (s, 1H), 6.20–6.15 (m, 1H), 4,98 (s, 2H); **^13^C{^1^H} NMR** (100 MHz, DMSO-*d*_6_) δ 133.5, 125.8, 123.8, 123.0, 121.2, 119.3, 118.2, 116.6, 112.6, 109.0, 107.6, 76.3; **HRMS** (ESI-QTOF) *m*/*z* [M+H]^+^ calcd. for C_13_H_11_N_4_ 223.0978, found 223.0970.

#### 3.1.2. General Procedure for the Synthesis of **5**

A mixture of **4** (25.0 mg, 0.11 mmol, 1.0 equiv), PhCHO (13.3 μL, 0.13 mmol, 1.2 equiv), and K_2_CO_3_ (28.9 mg, 0.22 mmol, 2.0 equiv) in DMSO (2 mL) was stirred at 130 °C (microwave heating was used) for 30 min. The reaction mixture was diluted with dichloromethane (5 mL) and washed with water (5 mL). The water layer was extracted with dichloromethane (5 mL) one more time. The organic layer was dried over MgSO_4_ and concentrated under reduced pressure to give the crude residue, which was purified by silica gel column chromatography (hexane/EtOAc:dichloromethane = 7:1:2) to afford **5a** (30.7 mg, 89%) as a yellow solid.

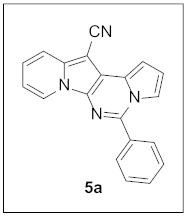
**5-Phenylpyrrolo[1′,2′:1,6]pyrimido[5,4-*b*]indolizine-12-carbonitrile (5a).** Yellow solid, m.p.: 232.5–233.2 °C (30.2 mg, 89%); **^1^H NMR** (400 MHz, CDCl_3_) δ 8.72 (d, *J* = 6.8 Hz, 1H), 7.93–7.87 (m, 2H), 7.74 (d, *J* = 8.8 Hz, 1H), 7.64–7.57 (m, 2H), 7.29–7.23 (m, 1H), 7.15 (d, *J* = 3.2 Hz, 1H), 6.94–6.87 (m, 2H); **^13^C{^1^H} NMR** (100 MHz, CDCl_3_) δ 144.1, 138.0, 134.0, 130.6, 129.0, 128.7, 128.5, 127.6, 125.2, 123.5, 117.3, 116.4, 115.1, 114.4, 112.6, 111.4, 101.2, 72.1; **HRMS** (ESI-QTOF) *m*/*z* [M+H]^+^ calcd. for C_20_H_13_N_4_ 309.1135, found 309.1135.
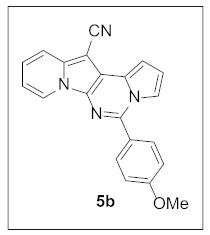
**5-(4-Methoxyphenyl)pyrrolo[1′,2′:1,6]pyrimido[5,4-*b*]indolizine-12-carbonitrile (5b).** Yellow solid, m.p.: 189.2–189.8 °C (23.8 mg, 64%); **^1^H NMR** (400 MHz, CDCl_3_) δ 8.71 (d, *J* = 6.8 Hz, 1H), 7.87 (d, *J* = 8.4 Hz, 2H), 7.72 (d, *J* = 8.8 Hz, 1H), 7.66–7.62 (m, 1H), 7.25–7.21 (m, 1H), 7.16–7.12 (m, 1H), 7.10 (d, *J* = 8.4 Hz, 2H), 6.92–6.86 (m, 2H), 3.93 (s, 3H); **^13^C{^1^H} NMR** (100 MHz, CDCl_3_) δ 161.3, 144.1, 137.9, 130.2, 128.7, 127.7, 126.4, 125.1, 123.5, 117.4, 116.6, 115.0, 114.4, 114.3, 112.6, 111.2, 101.1, 72.0, 55.5; **HRMS** (ESI-QTOF) *m*/*z* [M+H]^+^ calcd. for C_21_H_15_N_4_O 339.1240, found 339.1234.
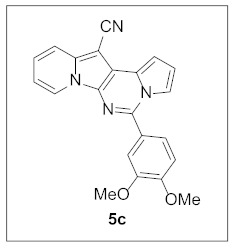
**5-(3,4-Dimethoxyphenyl)pyrrolo[1′,2′:1,6]pyrimido[5,4-*b*]indolizine-12-carbonitrile (5c).** Yellow solid, m.p.: 209.3–209.9 °C (29.2 mg, 72%); **^1^H NMR** (400 MHz, CDCl_3_) δ 8.71 (d, *J* = 6.8 Hz, 1H), 7.70 (d, *J* = 8.0 Hz, 1H), 7.68–7.64 (m, 1H), 7.52 (d, *J* = 8.4 Hz, 1H), 7.42 (s, 1H), 7.26–7.20 (m, 1H), 7.15–7.10 (m, 1H), 7.05 (d, *J* = 8.4 Hz, 1H), 6.93–6.84 (m, 2H), 4.00 (s, 3H), 3.97 (s, 3H); **^13^C{^1^H} NMR** (100 MHz, CDCl_3_) δ 151.0, 149.3, 144.1, 138.0, 128.6, 127.8, 126.5, 125.1, 123.6, 121.6, 117.4, 116.5, 115.1, 114.5, 112.6, 111.7, 111.3, 111.1, 101.2, 72.1, 56.2, 56.1; **HRMS** (ESI-QTOF) *m*/*z* [M+H]^+^ calcd. for C_22_H_17_N_4_O_2_ 369.1346, found 369.1336.
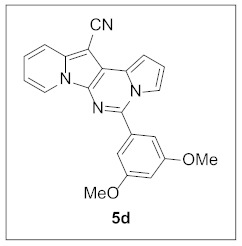
**5-(3,5-Dimethoxyphenyl)pyrrolo[1′,2′:1,6]pyrimido[5,4-*b*]indolizine-12-carbonitrile (5d).** Green solid, m.p.: 238.8–239.5 °C (36.1 mg, 89%); **^1^H NMR** (400 MHz, CDCl_3_) δ 8.72 (d, *J* = 6.8 Hz, 1H), 7.71 (d, *J* = 8.8 Hz, 1H), 7.66–7.62 (m, 1H), 7.28-7.20 (m, 1H), 7.15–7.10 (m, 1H), 7.01 (s, 2H), 6.93–6.84 (m, 2H), 6.67 (s, 1H), 3.88 (s, 6H); **^13^C{^1^H} NMR** (100 MHz, CDCl_3_) δ 161.2, 143.9, 138.0, 135.6, 128.4, 127.6, 125.3, 123.6, 117.4, 116.5, 115.1, 114.6, 112.6, 111.6, 106.7, 102.7, 101.2, 72.1, 55.7; **HRMS** (ESI-QTOF) *m*/*z* [M+H]^+^ calcd. for C_22_H_17_N_4_O_2_ 369.1346, found 369.1345.
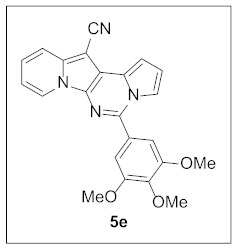
**5-(3,4,5-Trimethoxyphenyl)pyrrolo[1′,2′:1,6]pyrimido[5,4-*b*]indolizine-12-carbonitrile (5e).** Brown solid, m.p.: 244.8–245.4 °C (28.0 mg, 64%); **^1^H NMR** (400 MHz, CDCl_3_) δ 8.73 (d, *J* = 6.8 Hz, 1H), 7.71 (d, *J* = 7.6 Hz, 1H), 7.68–7.62 (m, 1H), 7.26–7.20 (m, 1H), 7.13 (s, 3H), 6.93–6.86 (m, 2H), 3.97 (s, 3H), 3.95 (s, 6H); **^13^C{^1^H} NMR** (100 MHz, CDCl_3_) δ 153.7, 143.9, 140.0, 138.0, 129.2, 128.4, 127.7, 125.3, 123.6, 117.4, 116.4, 115.2, 114.5, 112.7, 111.5, 106.0, 101.3, 72.1, 61.0, 56.4; **HRMS** (ESI-QTOF) *m*/*z* [M+H]^+^ calcd. for C_23_H_19_N_4_O_3_ 399.1452, found 399.1449.
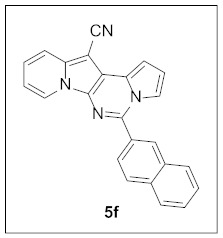
**5-(Naphthalen-2-yl)pyrrolo[1′,2′:1,6]pyrimido[5,4-*b*]indolizine-12-carbonitrile (5f).** Yellow solid, m.p.: 218.2–218.9 °C (26.0 mg, 66%); **^1^H NMR** (400 MHz, CDCl_3_) δ 8.77 (d, *J* = 7.2 Hz, 1H), 8.44 (s, 1H), 8.06 (d, *J* = 8.4 Hz, 1H), 8.00–7.93 (m, 3H), 7.77 (d, *J* = 7.6 Hz, 1H), 7.70 (d, *J* = 2.8 Hz, 1H), 7.64–7.59 (m, 2H), 7.31–7.27 (m, 1H), 7.19 (d, *J* = 3.6 Hz, 1H), 6.95–6.90 (m, 2H); **^13^C{^1^H} NMR** (100 MHz, CDCl_3_) δ 144.1, 138.1, 134.2, 133.0, 131.3, 128.8, 128.7, 128.6, 127.9, 127.8, 127.6, 127.0, 125.4, 125.3, 123.6, 117.5, 116.5, 115.3, 114.5, 112.7, 111.6, 101.3, 72.2; **HRMS** (ESI-QTOF) *m*/*z* [M+H]^+^ calcd. for C_24_H_15_N_4_ 359.1291, found 359.1285.
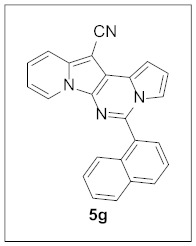
**5-(Naphthalen-1-yl)pyrrolo[1′,2′:1,6]pyrimido[5,4-*b*]indolizine-12-carbonitrile (5g).** Yellow solid, m.p.: 120.5–121.1 °C (31.9 mg, 81%); **^1^H NMR** (400 MHz, CDCl_3_) δ 8.73 (d, *J* = 6.8 Hz, 1H), 8.10 (d, *J* = 8.0 Hz, 1H), 7.99 (d, *J* = 8.0 Hz, 1H), 7.81 (d, *J* = 7.2 Hz, 1H), 7.78 (d, *J* = 9.6 Hz, 1H), 7.68 (t, *J* = 8.0 Hz, 1H), 7.56 (t, *J* = 7.1 Hz, 1H), 7.51 (d, *J* = 8.8 Hz, 1H), 7.42 (d, *J* = 7.2 Hz, 1H), 7.31–7.26 (m, 1H), 7.20-7.16 (m, 1H), 6.97-6.93 (m, 1H), 6.90 (t, *J* = 6.7 Hz, 1H), 6.82–6.77 (m, 1H); **^13^C{^1^H} NMR** (100 MHz, CDCl_3_) δ 143.4, 138.1, 133.8, 131.2, 130.9, 128.7, 128.4, 127.7, 127.4, 127.3, 126.7, 125.5, 125.4, 124.7, 123.7, 117.5, 116.5, 115.2, 114.9, 112.7, 111.9, 101.2, 72.3; **HRMS** (ESI-QTOF) *m*/*z* [M+H]^+^ calcd. for C_24_H_15_N_4_ 359.1291, found 359.1290.
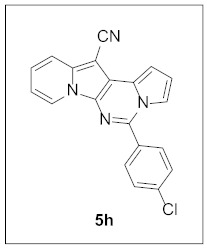
**5-(4-Chlorophenyl)pyrrolo[1′,2′:1,6]pyrimido[5,4-*b*]indolizine-12-carbonitrile (5h).** Yellow solid, m.p.: 230.0–230.8 9 °C (27.5 mg, 73%); **^1^H NMR** (400 MHz, CDCl_3_) δ 8.71 (d, *J* = 6.8 Hz, 1H), 7.87 (d, *J* = 7.2 Hz, 2H), 7.75 (d, *J* = 8.8 Hz, 1H), 7.62–7.54 (m, 3H), 7.29 (d, *J* = 7.6 Hz, 1H), 7.19–7.13 (m, 1H), 6.97–6.88 (m, 2H); **^13^C{^1^H} NMR** (100 MHz, CDCl_3_) δ 142.9, 138.2, 136.7, 132.4, 130.1, 129.3, 128.5, 127.7, 125.4, 123.5, 117.5, 116.3, 115.4, 114.1, 112.8, 111.6, 101.4, 72.2; **HRMS** (ESI-QTOF) *m*/*z* [M+H]^+^ calcd. for C_20_H_12_ClN_4_ 343.0745, found 343.0719.
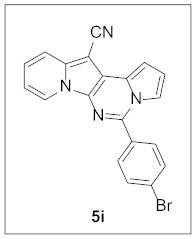
**5-(4-Bromophenyl)pyrrolo[1′,2′:1,6]pyrimido[5,4-*b*]indolizine-12-carbonitrile (5i).** Yellow solid, m.p.: 227.6–228.4 °C (32.8 mg, 77%); **^1^H NMR** (400 MHz, CDCl_3_) δ 8.68 (d, *J* = 6.8 Hz, 1H), 7.79 (d, *J* = 8.0 Hz, 2H), 7.76–7.69 (m, 3H), 7.57 (d, *J* = 2.8 Hz, 1H), 7.30–7.23 (m, 1H), 7.13 (d, *J* = 3.2 Hz, 1H), 6.94–6.87 (m, 2H); **^13^C{^1^H} NMR** (100 MHz, CDCl_3_) δ 142.9, 138.1, 132.9, 132.3, 130.2, 128.4, 127.7, 125.5, 125.0, 123.5, 117.4, 116.3, 115.4, 112.8, 111.6, 101.4, 72.2; **HRMS** (ESI-QTOF) *m*/*z* [M+H]^+^ calcd. for C_20_H_12_BrN_4_ 387.0240, found 387.0233.
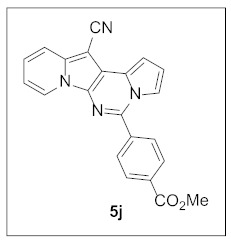
**Methyl 4-(12-cyanopyrrolo[1′,2′:1,6]pyrimido[5,4-*b*]indolizin-5-yl)benzoate (5j).** Yellow solid, m.p.: 231.2–231.7 °C (25.8 mg, 64%); **^1^H NMR** (400 MHz, CDCl_3_) δ 8.73 (d, *J* = 6.8 Hz, 1H), 8.26 (d, *J* = 8.0 Hz, 2H), 8.01 (d, *J* = 8.0 Hz, 2H), 7.75 (d, *J* = 8.8 Hz, 1H), 7.59 (s, 1H), 7.30 (d, *J* = 8.0 Hz, 1H), 7.19–7.14 (m, 1H), 6.97–6.88 (m, 2H), 4.00 (s, 3H); **^13^C{^1^H} NMR** (100 MHz, CDCl_3_) δ 166.3, 142.8, 138.3, 138.1, 131.9, 130.2, 128.7, 127.8, 125.6, 123.6, 117.5, 116.3, 115.5, 114.1, 112.8, 111.8, 101.5, 72.3, 52.5; **HRMS** (ESI-QTOF) *m*/*z* [M+H]^+^ calcd. for C_22_H_15_N_4_O_2_ 367.1190, found 367.1185.
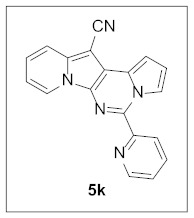
**5-(Pyridin-2-yl)pyrrolo[1′,2′:1,6]pyrimido[5,4-*b*]indolizine-12-carbonitrile (5k).** Green solid, m.p.: 192.0–192.6 °C (29.6 mg, 87%); **^1^H NMR** (400 MHz, CDCl_3_) δ 8.99–8.95 (m, 1H), 8.83–8.79 (m, 1H), 8.77 (d, *J* = 6.8 Hz, 1H), 8.43 (d, *J* = 8.0 Hz, 1H), 7.95 (t, *J* = 7.0 Hz, 1H), 7.74 (d, *J* = 9.0 Hz, 1H), 7.51–7.43 (m, 1H), 7.33-7.26 (m, 1H), 7.22–7.18 (m, 1H), 7.01–6.97 (m, 1H), 6.94 (t, *J* = 6.9 Hz, 1H); **^13^C{^1^H} NMR** (100 MHz, CDCl_3_) δ 152.9, 148.2, 140.0, 138.5, 136.8, 127.7, 127.6, 125.6, 124.9, 124.5, 123.5, 117.2, 117.0, 116.4, 115.1, 112.7, 112.5, 100.9, 72.2; **HRMS** (ESI-QTOF) *m*/*z* [M+H]^+^ calcd. for C_19_H_12_N_5_ 310.1087, found 310.1069.
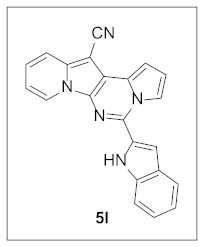
**5-(1*H*-Indol-2-yl)pyrrolo[1′,2′:1,6]pyrimido[5,4-*b*]indolizine-12-carbonitrile (5l).** Brown solid, m.p.: 270.8–271.4 °C (27.1 mg, 71%); **^1^H NMR** (400 MHz, CDCl_3_) δ 9.46 (s, 1H), 8.72 (d, *J* = 6.8 Hz, 1H), 8.22–8.18 (m, 1H), 7.75 (d, *J* = 8.0 Hz, 1H), 7.67 (d, *J* = 9.2 Hz, 1H), 7.53 (d, *J* = 8.0 Hz, 1H), 7.43 (s, 1H), 7.37 (t, *J* = 7.6 Hz, 1H), 7.25-7.19 (m, 1H), 7.17–7.13 (m, 1H), 7.03 (t, *J* = 3.2 Hz, 1H), 6.92 (t, *J* = 6.8 Hz, 1H); **^13^C{^1^H} NMR** (100 MHz, DMSO-*d*_6_) δ 138.2, 137.2, 136.3, 129.8, 128.5, 127.8, 127.6, 127.2, 124.8, 124.7, 122.1, 120.4, 117.3, 116.5, 116.3, 115.4, 113.8, 112.2, 110.4, 105.7, 100.6, 71.0; **HRMS** (ESI-QTOF) *m*/*z* [M+H]^+^ calcd. for C_22_H_14_N_5_ 348.1244, found 348.1215.
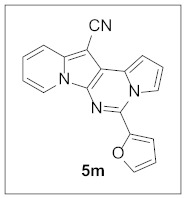
**5-(Furan-2-yl)pyrrolo[1′,2′:1,6]pyrimido[5,4-*b*]indolizine-12-carbonitrile (5m).** Yellow solid, m.p.: 238.1–238.6 °C (29.5 mg, 90%); **^1^H NMR** (400 MHz, CDCl_3_) δ 8.73 (d, *J* = 6.8 Hz, 1H), 8.38 (d, *J* = 2.8 Hz, 1H), 7.77–7.69 (m, 2H), 7.45 (d, *J* = 3.2 Hz, 1H), 7.30–7.27 (m, 1H), 7.17 (d, *J* = 4.0 Hz, 1H), 7.02–6.99 (m, 1H), 6.93 (t, *J* = 6.8 Hz, 1H), 6.71 (dd, *J* = 3.6, 1.6 Hz, 1H); **^13^C{^1^H} NMR** (100 MHz, CDCl_3_) δ 148.3, 144.3, 138.4, 134.3, 127.9, 125.5, 123.7, 117.5, 116.4, 115.7, 115.0, 114.4, 112.6, 112.2, 111.6, 101.0, 72.4; **HRMS** (ESI-QTOF) *m*/*z* [M+H]^+^ calcd. for C_18_H_11_N_4_O 299.0927, found 299.0920.
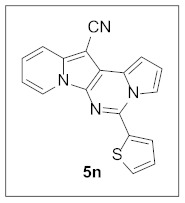
**5-(Thiophen-2-yl)pyrrolo[1′,2′:1,6]pyrimido[5,4-*b*]indolizine-12-carbonitrile (5n).** Yellow solid, m.p.: 220.8–221.5 °C (22.8 mg, 66%); **^1^H NMR** (400 MHz, CDCl_3_) δ 8.69 (d, *J* = 6.8 Hz, 1H), 8.04–7.99 (m, 1H), 7.93 (d, *J* = 4.4 Hz, 1H), 7.70 (d, *J* = 8.8 Hz, 1H), 7.60 (d, *J* = 5.2 Hz, 1H), 7.26-7.22 (m, 2H), 7.17–7.12 (m, 1H), 6.97 (t, *J* = 3.2 Hz, 1H), 6.91 (t, *J* = 7.2 Hz, 1H); **^13^C{^1^H} NMR** (100 MHz, CDCl_3_) δ 138.2, 138.0, 136.6, 129.0, 128.5, 128.0, 127.7, 125.4, 123.7, 117.4, 116.4, 115.6, 114.3, 112.7, 111.3, 101.4, 72.2; **HRMS** (ESI-QTOF) *m*/*z* [M+H]^+^ calcd. for C_18_H_11_N_4_S 315.0699, found 315.0714.
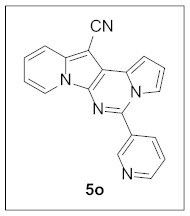
**5-(Pyridin-3-yl)pyrrolo[1′,2′:1,6]pyrimido[5,4-*b*]indolizine-12-carbonitrile (5o).** Yellow solid, m.p.: 238.3–238.9 °C (30.6 mg, 90%); **^1^H NMR** (400 MHz, CDCl_3_) δ 9.18 (s, 1H), 8.86–8.81 (m, 1H), 8.71 (d, *J* = 6.4 Hz, 1H), 8.25 (d, *J* = 8.0 Hz, 1H), 7.75 (d, *J* = 8.0 Hz, 1H), 7.60–7.51 (m, 2H), 7.29 (t, *J* = 7.9 Hz, 1H), 7.19–7.14 (m, 1H), 6.97–6.89 (m, 2H); **^13^C{^1^H} NMR** (100 MHz, CDCl_3_) δ 151.4, 149.7, 141.1, 138.3, 136.0, 130.2, 128.4, 127.8, 125.7, 123.60, 123.55, 117.5, 116.2, 115.8, 113.7, 112.9, 111.9, 101.6, 72.4; **HRMS** (ESI-QTOF) *m*/*z* [M+H]^+^ calcd. for C_19_H_12_N_5_ 310.1087, found 310.1091.
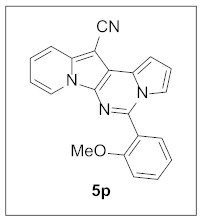
**5-(2-Methoxyphenyl)pyrrolo[1′,2′:1,6]pyrimido[5,4-*b*]indolizine-12-carbonitrile (5p).** Brown solid, m.p.: 245.4–245.9 °C (27.9 mg, 75%); **^1^H NMR** (400 MHz, CDCl_3_) δ 8.73 (d, *J* = 6.8 Hz, 1H), 7.75 (d, *J* = 8.8 Hz, 1H), 7.62–7.51 (m, 2H), 7.28–7.22 (m, 1H), 7.17 (t, *J* = 7.4 Hz, 1H), 7.15–7.08 (m, 2H), 7.08–7.04 (m, 1H), 6.98 (t, *J* = 6.8 Hz, 1H), 6.84 (t, *J* = 3.0 Hz, 1H), 3.77 (s, 3H); **^13^C{^1^H} NMR** (100 MHz, CDCl_3_) δ 157.5, 142.8, 137.9, 132.0, 131.0, 128.3, 126.9, 125.1, 123.7, 123.1, 121.2, 117.3, 116.6, 114.9, 114.7, 112.5, 111.8, 111.4, 100.8, 72.1, 55.8; **HRMS** (ESI-QTOF) *m*/*z* [M+H]^+^ calcd. for C_21_H_15_N_4_O 339.1240, found 339.1243.
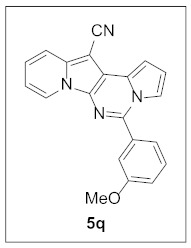
**5-(3-Methoxyphenyl)pyrrolo[1′,2′:1,6]pyrimido[5,4-*b*]indolizine-12-carbonitrile (5q).** Yellow solid, m.p.: 182.3–182.9 °C (27.5 mg, 74%); **^1^H NMR** (400 MHz, CDCl_3_) δ 8.72 (d, *J* = 6.8 Hz, 1H), 7.73 (d, *J* = 9.2 Hz, 1H), 7.64–7.60 (m, 1H), 7.53–7.45 (m, 2H), 7.41 (s, 1H), 7.25 (t, *J* = 8.0 Hz, 1H), 7.16–7.10 (m, 2H), 6.93–6.86 (m, 2H), 3.90 (s, 3H); **^13^C{^1^H} NMR** (100 MHz, CDCl_3_) δ 160.0, 143.9, 138.0, 135.2, 130.1, 128.5, 127.7, 125.2, 123.6, 120.8, 117.4, 116.5, 116.4, 115.1, 114.5, 114.2, 112.6, 111.5, 101.2, 72.1, 55.5; **HRMS** (ESI-QTOF) *m*/*z* [M+H]^+^ calcd. for C_21_H_15_N_4_O 339.1240, found 339.1241.
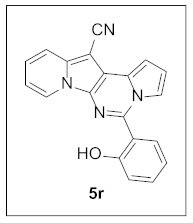
**5-(2-Hydroxyphenyl)pyrrolo[1′,2′:1,6]pyrimido[5,4-*b*]indolizine-12-carbonitrile (5r).** Yellow solid, m.p.: 248.4–249.1 °C (24.3 mg, 68%); **^1^H NMR** (400 MHz, CDCl_3_) δ 9.71 (s, 1H), 8.59 (d, *J* = 6.8 Hz, 1H), 7.98 (d, *J* = 7.6 Hz, 1H), 7.95–7.92 (m, 1H), 7.75 (d, *J* = 9.2 Hz, 1H), 7.47 (t, *J* = 7.6 Hz, 1H), 7.29 (t, *J* = 8.0 Hz, 1H), 7.20 (d, *J* = 8.4 Hz, 1H), 7.17 (d, *J* = 7.2 Hz, 1H), 7.08 (t, *J* = 8.0 Hz, 1H), 6.99–6.93 (m, 2H); **^13^C{^1^H} NMR** (100 MHz, CDCl_3_) δ 156.3, 142.2, 138.3, 132.6, 127.9, 127.7, 126.4, 125.6, 123.0, 119.7, 118.3, 117.7, 117.3, 116.0, 115.9, 115.4, 113.3, 111.9, 102.1, 72.8; **HRMS** (ESI-QTOF) *m*/*z* [M+H]^+^ calcd. for C_20_H_13_N_4_O 325.1084, found 325.1074.
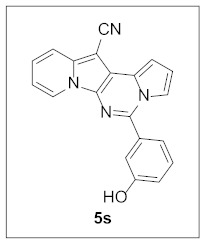
**5-(3-Hydroxyphenyl)pyrrolo[1′,2′:1,6]pyrimido[5,4-*b*]indolizine-12-carbonitrile (5s).** Green solid, m.p.: 318.7–319.3 °C (28.9 mg, 81%); **^1^H NMR** (400 MHz, DMSO-*d*_6_) δ 9.93 (s, 1H), 8.85 (d, *J* = 6.8 Hz, 1H), 7.83 (d, *J* = 8.8 Hz, 1H), 7.69–7.66 (m, 1H), 7.49-7.41 (m, 2H), 7.34 (d, *J* = 7.6 Hz, 1H), 7.29 (s, 1H), 7.13 (t, *J* = 6.8 Hz, 1H), 7.05 (d, *J* = 8.0 Hz, 1H), 6.99–6.93 (m, 2H); **^13^C{^1^H} NMR** (100 MHz, DMSO-*d*_6_) δ 158.2, 144.2, 137.9, 134.9, 130.6, 128.4, 127.3, 127.1, 124.5, 119.6, 118.2, 117.3, 116.5, 115.9, 115.7, 115.3, 114.1, 110.8, 100.6, 70.8; **HRMS** (ESI-QTOF) *m*/*z* [M+H]^+^ calcd. for C_20_H_13_N_4_O 325.1084, found 325.1090.
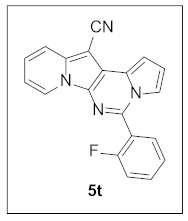
**5-(2-Fluorophenyl)pyrrolo[1′,2′:1,6]pyrimido[5,4-*b*]indolizine-12-carbonitrile (5t).** Yellow solid, m.p.: 220.2–220.7 °C (19.0 mg, 53%); **^1^H NMR** (400 MHz, CDCl_3_) δ 8.72 (d, *J* = 6.8 Hz, 1H), 7.78–7.69 (m, 2H), 7.67–7.56 (m, 1H), 7.40 (t, *J* = 7.5 Hz, 1H), 7.36–7.22 (m, 3H), 7.19–7.15 (m, 1H), 6.95–6.88 (m, 2H); **^13^C{^1^H} NMR** (100 MHz, CDCl_3_) δ 161.5, 159.0, 139.6, 138.2, 132.5 (d, *J* = 8.2 Hz), 131.3 (d, *J* = 2.4 Hz), 128.1, 127.2, 125.5, 125.0 (d, *J* = 3.4 Hz), 123.7, 117.5, 116.45, 116.41, 115.3, 114.4 (d, *J* = 2.2 Hz), 112.7, 112.1, 101.3, 72.3; **HRMS** (ESI-QTOF) *m*/*z* [M+H]^+^ calcd. for C_20_H_12_FN_4_ 327.1041, found 327.1040.
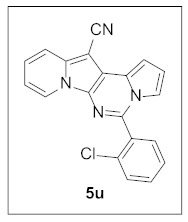
**5-(2-Chlorophenyl)pyrrolo[1′,2′:1,6]pyrimido[5,4-*b*]indolizine-12-carbonitrile (5u).** Yellow solid, m.p.: 249.2–249.7 °C (25.3 mg, 67%); **^1^H NMR** (400 MHz, CDCl_3_) δ 8.72 (d, *J* = 6.8 Hz, 1H), 7.76(d, *J* = 8.8 Hz, 1H), 7.65–7.59 (m, 2H), 7.56 (t, *J* = 7.6, 1H), 7.51 (t, *J* = 7.6, 1H), 7.31–7.26 (m, 1H), 7.20–7.14 (m, 1H), 7.06–7.01 (m, 1H), 6.94–6.88 (m 2H); **^13^C{^1^H} NMR** (100 MHz, CDCl_3_) δ 141.6, 138.2, 133.6, 133.1, 131.7, 131.1, 130.3, 128.0, 127.6, 127.0, 125.5, 123.7, 117.4, 116.4, 115.5, 114.2, 112.7, 112.1, 101.3, 72.3; **HRMS** (ESI-QTOF) *m*/*z* [M+H]^+^ calcd. for C_20_H_12_ClN_4_ 343.0745, found 343.0743.
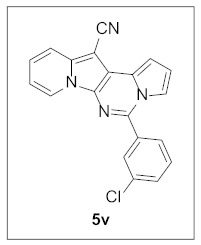
**5-(3-Chlorophenyl)pyrrolo[1′,2′:1,6]pyrimido[5,4-*b*]indolizine-12-carbonitrile (5v).** Yellow solid, m.p.: 243.1–243.8 °C (26.8 mg, 71%); **^1^H NMR** (400 MHz, CDCl_3_) δ 8.71 (d, *J* = 6.4 Hz, 1H), 7.90 (s, 1H), 7.81 (d, *J* = 6.8 Hz, 1H), 7.74 (d, *J* = 9.2 Hz, 1H), 7.62–7.50 (m, 3H), 7.29 (d, *J* = 7.2 Hz, 1H), 7.19–7.13 (m, 1H), 6.97–6.89 (m, 2H); **^13^C{^1^H} NMR** (100 MHz, CDCl_3_) δ 142.5, 138.2, 135.6, 135.1, 130.7, 130.2, 129.0, 128.4, 127.7, 126.7, 125.5, 123.6, 117.5, 116.3, 115.5, 114.1, 112.8, 111.8, 101.5, 72.3; **HRMS** (ESI-QTOF) *m*/*z* [M+H]^+^ calcd. for C_20_H_12_ClN_4_ 343.0745, found 343.0739.
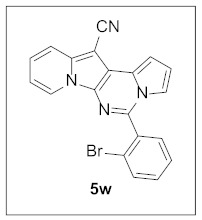
**5-(2-Bromophenyl)pyrrolo[1′,2′:1,6]pyrimido[5,4-*b*]indolizine-12-carbonitrile (5w).** Yellow solid, m.p.: 222.1–222.6 °C (19.6 mg, 46%); **^1^H NMR** (400 MHz, CDCl_3_) δ 8.74 (d, *J* = 6.8 Hz, 1H), 7.93–7.87 (m, 2H), 7.76 (d, *J* = 8.8 Hz, 1H), 7.64–7.58 (m, 3H), 7.31–7.27 (m, 1H), 7.16 (d, *J* = 3.6 Hz, 1H), 6.96–6.88 (m, 2H); **^13^C{^1^H} NMR** (100 MHz, CDCl_3_) δ 138.1, 134.0, 130.6, 129.0, 128.7, 127.7, 125.3, 123.6, 120.6, 117.5, 116.5, 115.2, 114.4, 112.7, 111.6, 104.0, 101.6, 101.3, 72.2; **HRMS** (ESI-QTOF) *m*/*z* [M+H]^+^ calcd. for C_20_H_12_BrN_4_ 387.0240, found 387.0208.
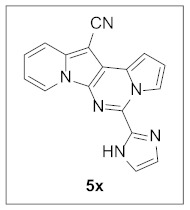
**5-(1*H*-Imidazol-2-yl)pyrrolo[1′,2′:1,6]pyrimido[5,4-*b*]indolizine-12-carbonitrile (5x).** Dark green solid, m.p.: 304.1–304.9 °C (24.3 mg, 74%); **^1^H NMR** (400 MHz, DMSO-*d*_6_) δ 13.38 (s, 1H), 9.46–9.39 (m, 1H), 9.05 (d, *J* = 6.8 Hz, 1H), 7.83 (d, *J* = 8.8 Hz, 1H), 7.63 (s, 1H), 7.48 (t, *J* = 5.8 Hz, 1H), 7.36 (s, 1H), 7.24 (t, *J* = 6.0 Hz, 1H), 7.09–7.03 (m, 1H), 7.00–6.94 (m, 1H); **^13^C{^1^H} NMR** (100 MHz, DMSO-*d*_6_) δ 141.1, 138.1, 133.0, 130.7, 127.3, 127.1, 124.8, 120.7, 117.2, 116.4, 115.6, 113.7, 110.7, 100.2, 71.0; **HRMS** (ESI-QTOF) *m*/*z* [M+H]^+^ calcd. for C_17_H_11_N_6_ 299.1040, found 299.1027.
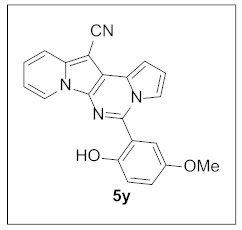
**5-(2-Hydroxy-5-methoxyphenyl)pyrrolo[1′,2′:1,6]pyrimido[5,4-*b*]indolizine-12-carbonitrile (5y).** Yellow solid, m.p.: 240.3–240.7 °C (14.4 mg, 37%); **^1^H NMR** (400 MHz, DMSO-*d*_6_) δ 9.69 (s, 1H), 8.88 (d, *J* = 6.6 Hz, 1H), 7.85 (d, *J* = 8.4 Hz, 1H), 7.47 (d, *J* = 8.0 Hz, 1H), 7.20 (s, 1H), 7.16–7.04 (m, 3H), 7.00 (d, *J* = 8.8 Hz, 1H), 6.95–6.89 (m, 2H), 3.73 (s, 3H); **^13^C{^1^H} NMR** (100 MHz, CDCl_3_) δ 152.7, 150.0, 142.0, 138.3, 127.8, 126.5, 125.6, 123.1, 119.2, 119.0, 117.7, 116.0, 115.3, 113.2, 112.4, 111.9, 102.1, 72.7, 56.0; **HRMS** (ESI-QTOF) *m*/*z* [M+H]^+^ calcd. for C_21_H_15_N_4_O_2_ 355.1190, found 355.1201.
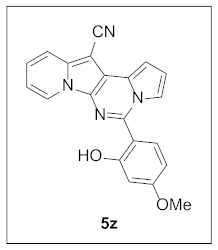
**5-(2-Hydroxy-4-methoxyphenyl)pyrrolo[1′,2′:1,6]pyrimido[5,4-*b*]indolizine-12-carbonitrile (5z).** Yellow solid, m.p.: 235.7–236.4 °C (34.7 mg, 89%); **^1^H NMR** (400 MHz, CDCl_3_) δ 10.31 (s, 1H), 8.57 (d, *J* = 6.8 Hz, 1H), 7.96 (s, 1H), 7.94 (d, *J* = 7.2 Hz, 1H), 7.75 (d, *J* = 8.8 Hz, 1H), 7.31–7.27 (m, 1H), 7.16 (d, *J* = 2.4 Hz, 1H), 6.98–6.93 (m, 2H), 6.70 (d, *J* = 2.4 Hz, 1H), 6.64 (dd, *J* = 8.8 Hz, 2.4 Hz, 1H), 3.90 (s, 3H); **^13^C{^1^H} NMR** (100 MHz, CDCl_3_) δ 162.9, 158.6, 142.7, 138.0, 128.8, 127.9, 126.3, 125.2, 122.9, 117.6, 116.0, 115.8, 115.4, 113.1, 111.3, 109.9, 106.8, 102.5, 102.0, 72.6, 55.5; **HRMS** (ESI-QTOF) *m*/*z* [M+H]^+^ calcd. for C_21_H_15_N_4_O_2_ 355.1190, found 355.1194.
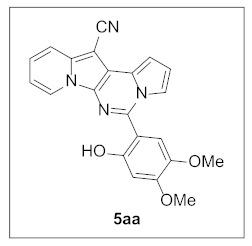
**5-(2-Hydroxy-4,5-dimethoxyphenyl)pyrrolo[1′,2′:1,6]pyrimido[5,4-*b*]indolizine-12-carbonitrile (5aa).** Yellow solid, m.p.: 243.7–244.4 °C (29.6 mg, 70%); **^1^H NMR** (400 MHz, CDCl_3_) δ 9.86 (s, 1H), 8.56 (d, *J* = 6.8 Hz, 1H), 7.98–7.95 (m, 1H), 7.76 (d, *J* = 8.8 Hz, 1H), 7.45 (s, 1H), 7.31–7.27 (m, 1H), 7.16 (d, *J* = 3.6 Hz, 1H), 6.99–6.92 (m, 2H), 6.72 (s, 1H), 3.98 (s, 3H), 3.89 (s, 3H); **^13^C{^1^H} NMR** (100 MHz, CDCl_3_) δ 152.7, 152.1, 142.7, 142.4, 137.9, 127.6, 126.4, 125.3, 122.8, 117.6, 115.9, 115.2, 113.2, 111.1, 110.4, 108.2, 102.0, 101.9, 72.6, 56.8, 56.1; **HRMS** (ESI-QTOF) *m*/*z* [M+Na]^+^ calcd. for C_22_H_16_N_4_NaO_3_ 407.1115, found 407.1140.
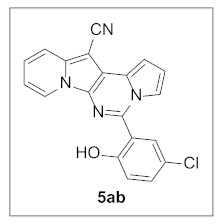
**5-(5-Chloro-2-hydroxyphenyl)pyrrolo[1′,2′:1,6]pyrimido[5,4-*b*]indolizine-12-carbonitrile (5ab).** Brown solid, m.p.: 276.5–277.3 °C (15.0 mg, 38%); **^1^H NMR** (400 MHz, DMSO-*d*_6_) δ 10.49 (s, 1H), 8.88 (d, *J* = 6.8 Hz, 1H), 7.83 (d, *J* = 8.8 Hz, 1H), 7.62 (s, 1H), 7.52 (d, *J* = 8.4 Hz, 1H), 7.45 (t, *J* = 7.9 Hz, 1H), 7.22 (s, 1H), 7.14–7.05 (m, 2H), 6.95–6.90 (m, 2H); **^13^C{^1^H} NMR** (100 MHz, DMSO-*d*_6_) δ 154.6, 141.6, 137.6, 131.6, 130.4, 127.7, 126.8, 125.9, 124.3, 122.8, 122.4, 118.0, 116.9, 116.1, 115.4, 115.0, 113.7, 110.8, 99.9, 70.4; **HRMS** (ESI-QTOF) *m*/*z* [M+H]^+^ calcd. for C_20_H_12_ClN_4_O 359.0694, found 359.0693.

#### 3.1.3. Synthesis of **8**

A mixture of **4** (25.0 mg, 0.11 mmol, 1.0 equiv) and DMFDMA (17.3 μL, 0.13 mmol, 1.2 equiv) in toluene (1 mL) was stirred at 130 °C for 24 h. The reaction mixture was concentrated under reduced pressure to give the crude residue, which was purified by silica gel column chromatography (hexane:EtOAc:dichloromethane = 5:1:2) to afford **8** (23.8 mg, 93%) as a green solid.

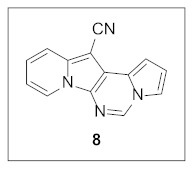
**Pyrrolo[1′,2′:1,6]pyrimido[5,4-*b*]indolizine-12-carbonitrile (8).** Green solid, m.p.: 248.8–249.5 °C (23.8 mg, 93%); **^1^H NMR** (400 MHz, CDCl_3_) δ 8.63 (d, *J* = 6.8 Hz, 1H), 8.55 (s, 1H), 7.69 (d, *J* = 8.8 Hz, 1H), 7.51–7.45 (m, 1H), 7.29–7.21 (m, 1H), 7.01 (d, *J* = 3.6 Hz, 1H), 6.89–6.95 (m, 2H); **^13^C{^1^H} NMR** (100 MHz, CDCl_3_) δ 137.9, 134.5, 127.7, 126.1, 125.3, 123.4, 117.4, 116.3, 115.9, 113.2, 112.8, 112.5, 100.8, 72.4; **HRMS** (ESI-QTOF) *m*/*z* [M+H]^+^ calcd. for C_14_H_9_N_4_ 233.0822, found 233.0820.

#### 3.1.4. Synthesis of **9**

Compound **9** was prepared with 2-pyridylacetonitrile (472.1 μL, 4.23 mmol, 1.0 equiv) by following the same procedures for the synthesis of **4**.

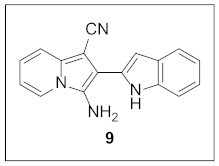
**3-Amino-2-(1*H*-indol-2-yl)indolizine-1-carbonitrile (9).** Green solid, m.p.: 223.9–224.4 °C (967.6 mg, 84%); **^1^H NMR** (400 MHz, DMSO-*d*_6_) δ 11.20 (s, 1H), 8.30 (d, *J* = 6.8 Hz, 1H), 7.58 (d, *J* = 8.4 Hz, 1H), 7.55 (d, *J* = 9.2 Hz, 1H), 7.49 (d, *J* = 8.0 Hz, 1H), 7.12 (t, *J* = 7.4 Hz, 1H), 7.08–7.00 (m, 2H), 6.91 (t, *J* = 6.8 Hz, 1H), 6.83 (s, 1H), 5.40 (s, 2H); **^13^C{^1^H} NMR** (100 MHz, DMSO-*d*_6_) δ 137.1, 133.8, 131.0, 128.7, 128.3, 123.1, 121.7, 121.5, 120.1, 119.8, 117.9, 116.9, 112.9, 111.9, 106.0, 100.7, 77.0; **HRMS** (ESI-QTOF) *m*/*z* [M+H]^+^ calcd. for C_17_H_13_N_4_ 273.1135, found 273.1140.

#### 3.1.5. Synthesis of **10**

Compounds **10** were prepared with **9** (30.0 mg, 0.11 mmol, 1.0 equiv) by following the same procedures for the synthesis of **5**.

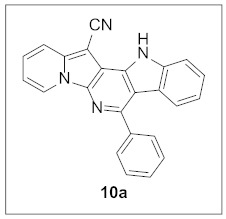
**7-Phenyl-12*H*-indolizino[2′,3′:5,6]pyrido[4,3-*b*]indole-13-carbonitrile (10a).** Yellow solid, m.p.: 329.6–330.1 °C (17.3 mg, 44%); **^1^H NMR** (400 MHz, DMSO-*d*_6_) δ 12.50 (s, 1H), 9.13 (d, *J* = 6.8 Hz, 1H), 7.92 (d, *J* = 8.8 Hz, 1H), 7.86–7.79 (m, 3H), 7.68–7.62 (m, 2H), 7.59 (d, *J* = 8.4 Hz, 1H), 7.52–7.41 (m, 2H), 7.16–7.09 (m, 1H); **^13^C{^1^H} NMR** (100 MHz, DMSO-*d*_6_) δ 149.6, 140.6, 140.5, 140.4, 137.9, 136.9, 129.6, 129.3, 129.2, 128.9, 126.1, 125.5, 121.8, 121.3, 120.7, 117.5, 117.0, 114.2, 113.1, 106.2, 69.0; **HRMS** (ESI-QTOF) *m*/*z* [M+H]^+^ calcd. for C_24_H_15_N_4_ 359.1291, found 359.1293.
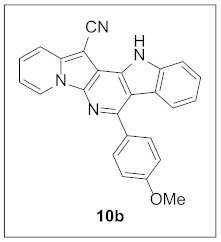
**7-(4-Methoxyphenyl)-12*H*-indolizino[2′,3′:5,6]pyrido[4,3-*b*]indole-13-carbonitrile (10b).** Green solid, m.p.: 356.3–356.8 °C (24.4 mg, 57%); **^1^H NMR** (400 MHz, DMSO-*d*_6_) δ 12.45 (s, 1H), 9.10 (d, *J* = 6.8 Hz, 1H), 7.90 (d, *J* = 8.8 Hz, 1H), 7.81 (d, *J* = 6.8 Hz, 1H), 7.78 (d, *J* = 8.8 Hz, 2H), 7.63–7.55 (m, 2H), 7.45 (t, *J* = 7.6 Hz, 1H), 7.19 (d, *J* = 8.8 Hz, 2H), 7.17–7.08 (m, 2H), 3.91 (s, 3H); **^13^C{^1^H} NMR** (100 MHz, DMSO-*d*_6_) δ 164.9, 154.3, 145.2, 145.1, 142.7, 141.7, 137.7, 135.8, 134.0, 130.8, 130.2, 126.7, 126.2, 125.5, 122.3, 121.8, 119.0, 117.9, 110.7, 73.7, 60.5; **HRMS** (ESI-QTOF) *m*/*z* [M+H]^+^ calcd. for C_25_H_17_N_4_O 389.1397, found 389.1398.
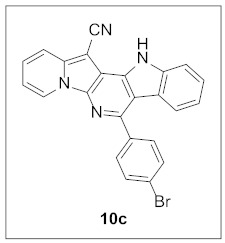
**7-(4-Bromophenyl)-12*H*-indolizino[2′,3′:5,6]pyrido[4,3-*b*]indole-13-carbonitrile (10c).** Brown solid, m.p.: 352.1–352.9 °C (23.6 mg, 49%); **^1^H NMR** (400 MHz, DMSO-*d*_6_) δ 12.50 (s, 1H), 9.09 (d, *J* = 6.8 Hz, 1H), 7.90 (d, *J* = 8.8 Hz, 1H), 7.87–7.82 (m, 3H), 7.79 (d, *J* = 8.4 Hz, 2H), 7.59 (t, *J* = 7.6 Hz, 1H), 7.53 (d, *J* = 8.0 Hz, 1H), 7.46 (t, *J* = 7.0 Hz, 1H), 7.20–7.08 (m, 2H); **^13^C{^1^H} NMR** (100 MHz, DMSO-*d*_6_) δ 148.2, 141.1, 140.4, 137.5, 136.4, 133.1, 131.5, 131.2, 129.5, 128.6, 126.2, 125.6, 122.9, 121.8, 121.1, 120.7, 117.6, 116.9, 115.1, 113.1, 106.7, 69.2; **HRMS** (ESI-QTOF) *m*/*z* [M+H]^+^ calcd. for C_24_H_14_BrN_4_ 437.0396, found 437.0398.
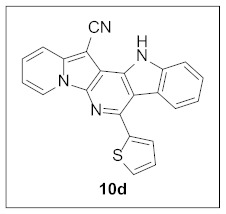
**7-(Thiophen-2-yl)-12*H*-indolizino[2′,3′:5,6]pyrido[4,3-*b*]indole-13-carbonitrile (10d).** Yellow solid, m.p.: 326.8–327.3 °C (33.7 mg, 84%); **^1^H NMR** (400 MHz, DMSO-*d*_6_) δ 12.54 (s, 1H), 9.09 (d, *J* = 6.8 Hz, 1H), 7.98 (d, *J* = 8.0 Hz, 1H), 7.93 (d, *J* = 8.8 Hz, 1H), 7.87 (d, *J* = 5.2 Hz, 1H), 7.84 (d, *J* = 8.4 Hz, 1H), 7.79–7.73 (m, 1H), 7.62 (t, *J* = 7.8 Hz, 1H), 7.50 (d, *J* = 8.0 Hz, 1H), 7.39–7.34 (m, 1H), 7.23 (t, *J* = 7.6 Hz, 1H), 7.17 (t, *J* = 7.2 Hz, 1H); **^13^C{^1^H} NMR** (100 MHz, DMSO-*d*_6_) δ 142.8, 142.7, 140.7, 140.5, 137.6, 137.2, 129.7, 128.6, 128.5, 128.0, 126.4, 125.5, 121.7, 121.4, 120.9, 117.6, 116.9, 114.3, 113.4, 113.3, 106.5, 69.2; **HRMS** (ESI-QTOF) *m*/*z* [M+H]^+^ calcd. for C_22_H_13_N_4_S 365.0855, found 365.0879.

### 3.2. Bioassay

#### 3.2.1. Cell Culture

HepG2, Huh7, H1299, HT29, AGS and A549 cells were purchased from the Korean Cell Line Bank (Seoul, Korea) and HaCaT cells were obtained from Prof. Sohee Kwon (Yonsei University, Korea). Liver cancer cells (HepG2), human keratinocyte cells (HaCaT), and lung cancer cells (A549) were grown in DMEM high-glucose medium, liver cancer cells (Huh7) and non-small cell lung cancer cells (H1299) were grown in RPMI 1640, and gastric cancer cells (AGS) were grown in Coon’s modified F12 medium. All media were supplemented with 10% FBS, 100 Units/mL penicillin, and 100 μg/mL streptomycin. All cells were incubated at 37 °C, 5% CO_2_ and 95% humidity.

#### 3.2.2. Cell Viability Assay

Cell viability assay was conducted using Cell Titer 96^®^ AQueous One Solution Cell proliferation Assay kit (Promega, Madison, WI, USA). Five cancer cells and HaCaT cells were plated in 96-well microplates with growth medium for 24 h. When cell confluency reached ~30%, cells were treated with DMSO (vehicle) and test compounds. The medium and test compounds were replaced every 24 h. To measure cell viability, the cells were incubated with MTS solution for 1 h. The absorbance was measured by using Infinite M200 microplate reader (Tecan, Männedorf, Switzerland) at 490 nm.

#### 3.2.3. In Vitro Wound-Healing Assay

The suppressive effect of **5r** on cell migration was assessed through in vitro wound-healing assay. HepG2 and Huh7 cells were grown to approximately 100% confluence to form a monolayer in a 96-well microplate. Wounds were formed through 96-well wound maker (Essen BioScience, Ann Arbor, MI, USA). Then, growth medium was removed and washed out twice with phosphate-buffered saline (PBS) and incubated with 200 μL of DMEM and RPMI 1640 medium containing **5r** or vehicle (DMSO). Images of the wound area were taken by using IncuCyte ZOOM (Essen BioScience, Ann Arbor MI, USA), and the percentage of wound closure was measured by using IncuCyte software.

#### 3.2.4. Caspase-3 Activity Assay

HepG2 and Huh7 cells were plated in 96-black-well plate to about 40% confluence, and then **5r** was treated for 24 h. To assess activity of caspase-3, the growth medium was changed with 100 μL of PBS containing 1 μM caspase-3 substrate, NucView 488, and incubated at room temperature. After 20 min, 1 μM Hoechst 33342 was treated to stain the cells. Activity of caspase-3 was suppressed by Ac-DEVD-CHO, a selective caspase-3 inhibitor. FLUOstar Omega microplate reader (BMG Labtech, Ortenberg, Germany) was used to assess the fluorescence of NucView 488 and Hoechst, and Lionheart FX Automated Microscope (BioTek, Winooski, VT, USA) was used to acquire the fluorescence microscopy images.

#### 3.2.5. Western Blot Analysis

For Western blot analysis, HepG2 and Huh7 cells were lysed with RIPA buffer (50 mM Tris-HCl, PH 7.4, 1% Nonidet P-40, 0.25% sodium deoxycholate, 150 mM NaCl, 1 mM EDTA, 1 mM Na_3_VO_4_, and protease inhibitor). Lysed samples were centrifuged at 13,000 RPM for 20 min at 4 °C to remove the cell debris, and equal amounts (60 μg protein/lane) of supernatant protein were divided by 4–12% Tris Glycine Precast Gel (KOMA BIOTECH, Seoul, Korea). Then, PVDF membranes (Millipore, Billerica, MA, USA) were used to transfer the separated proteins. Membrane blocking was performed by Tris-buffered saline with 0.1% Tween 20 (TBST) containing 5% bovine serum albumin (BSA) at room temperature for 50 min. The membranes were incubated with primary antibodies overnight at 4 °C with the indicated primary antibodies: anti-cleaved PARP (BD Biosciences) and anti-β-actin (Santa Cruz Biotechnology, Dallas, TX, USA). After incubating overnight, the membranes were washed out three times in 0.1% TBST and incubated with horseradish-peroxidase-conjugated secondary antibodies for 1 h. After 1 h, membranes were detected by using ECL Plus Western blotting detection system (GE Healthcare, Piscataway, NJ, USA).

#### 3.2.6. Flow Cytometry Analysis

HepG2 cells were grown to approximately 40% confluence in a 6-well plate and then **5r** was treated for 24 h. After 24 h, HepG2 cells were washed out twice with PBS and centrifuged at 1000 RPM for 2 min at room temperature. The cells were stained with propidium iodide for 15 min and then cell cycle phases were measured by using FACS (Beckman Coulter, Fullerton, CA, USA).

## 4. Conclusions

In summary, a new indolizine fused with pyrrolo[1,2-*c*]pyrimidine was designed and synthesized via one-pot three-component coupling followed by oxidative cyclization. This two-step protocol enabled the rapid construction of a novel tetracyclic heteroaromatic chemical library. The biological investigation of these compounds in five cancer cells revealed that some reduced the cell viability of HepG2 and Huh7 cells more strongly than those of the other three cancer cells. The structure–activity relationship study of these compounds led us to identify **5r** showing potent and selective anticancer activity against liver cancer cells (HepG2 and Huh7). Notably, **5r** had a weak inhibitory effect on the cell viability in nontumorigenic human keratinocytes (HaCaT) cells. Moreover, **5r** strongly inhibited cell migration and induced apoptosis through increase of caspase-3 activity and cleavage of PARP in a dose-dependent manner in HepG2 and Huh7 cells. In addition, co-treatment of **5r** with gemcitabine showed additional effect on cell viability in HegG2 and Huh7 cells. Taken together, these results suggest that **5r** could be used for the development of novel anticancer agents with distinctive polyheterocyclic pharmacophore to overcome the limitations of current liver cancer therapeutics.

## Figures and Tables

**Table 1:**
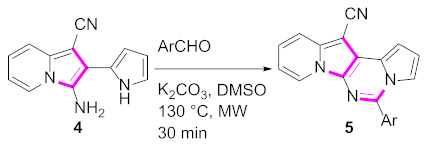
Synthesis of **5** *^a,b^*.

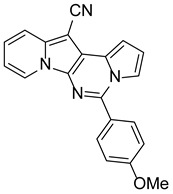	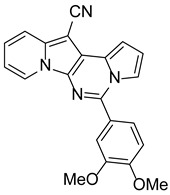	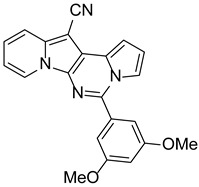
**5b** (64%)	**5c** (72%)	**5d** (89%)
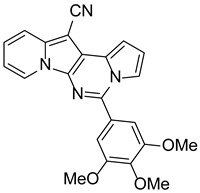	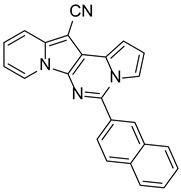	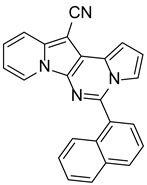
**5e** (64%)	**5f** (66%)	**5g** (81%)
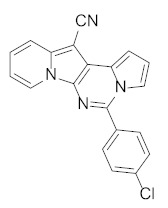	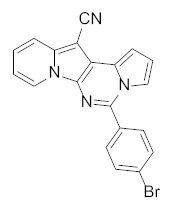	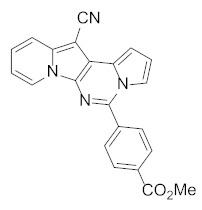
**5h** (73%)	**5i** (77%)	**5j** (64%)
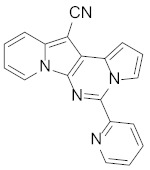	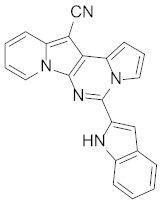	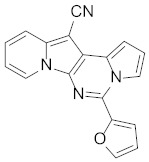
**5k** (87%)	**5l** (71%)	**5m** (90%)
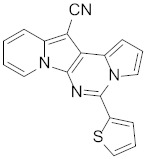		
**5n** (66%)		

*^a^* A mixture of **4** (25.0 mg, 0.11 mmol, 1.0 equiv), ArCHO (1.2 equiv), and K_2_CO_3_ (2.0 equiv) in DMSO (2 mL) was stirred at 130 °C (microwave heating) for 30 min. *^b^* Isolated yield (%).

**Table 2:** Cytotoxicity of **5**, **8**, and **10** in HepG2, H1299, HT29, AGS and A549 cells (mean, *n* = 3).

Compound	% Inhibition of Cell Viability at 10 μM				
	HepG2	H1299	HT29	AGS	A549
**8**	<25	<25	<25	<25	<25
**5a**	85.0	81.5	<25	<25	<25
**5b**	57.0	79.1	<25	<25	25.5
**5c**	76.7	94.0	44.0	56.0	47.1
**5d**	57.3	83.4	<25	<25	<25
**5e**	37.7	89.0	<25	<25	25.3
**5f**	92.5	88.4	<25	<25	<25
**5g**	49.2	82.8	<25	<25	<25
**5h**	88.4	80.0	26.9	31.0	52.5
**5i**	87.4	92.1	<25	<25	36.7
**5j**	42.2	52.8	<25	<25	<25
**5k**	90.6	83.8	28.8	<25	29.0
**5l**	99.2	62.3	58.6	<25	46.9
**5m**	64.0	81.9	<25	<25	<25
**5n**	98.9	70.3	48.4	<25	38.0
**10a**	<25	68.8	61.2	48.4	45.9
**10b**	<25	<25	<25	<25	<25
**10c**	39.6	<25	<25	<25	<25

**Table 3:** Inhibitory effect of **5n–x** and **10d** on cell viability of HepG2, Huh7, and HaCaT cells (mean, *n* = 5).

Compound	Structure	IC_50_ (μM)		
		HepG2	Huh7	HaCaT
**5n**	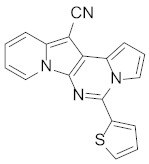	0.28	0.75	13.34
**5o**	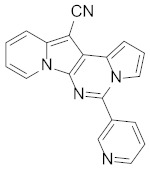	2.00	1.47	3.98
**5p**	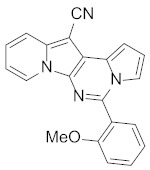	3.42	2.25	16.23
**5q**	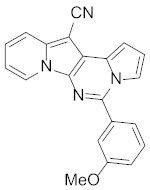	1.31	1.45	8.30
**5r**	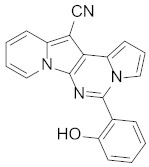	0.22	0.10	6.91
**5s**	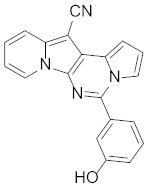	2.94	4.02	8.49
**5t**	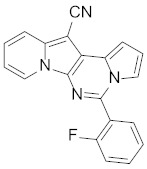	1.58	1.92	6.78
**5u**	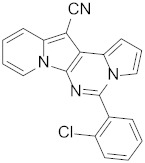	2.10	3.95	11.52
**5v**	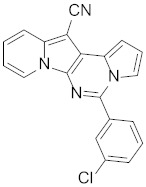	1.48	1.33	15.33
**5w**	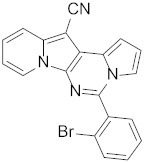	1.75	2.72	9.53
**5x**	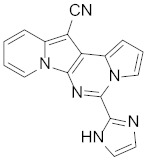	3.52	7.85	24.05
**10d**	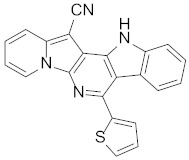	2.27	0.60	0.81

**Table 4:** Inhibitory effect of **5y–ab** on cell viability of HepG2, Huh7, and HaCaT cells (mean, *n* = 5).

Compound	Structure	IC_50_ (μM)		
		HepG2	Huh7	HaCaT
**5r**	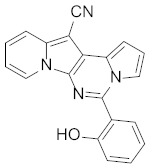	0.22	0.10	6.91
**5y**	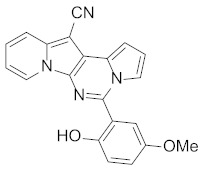	0.79	0.64	2.25
**5z**	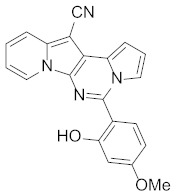	0.64	0.71	1.95
**5aa**	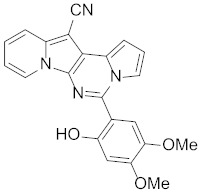	1.17	0.87	2.21
**5ab**	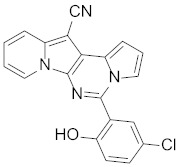	0.84	0.71	2.30

## Data Availability

Data is contained within the article and Supplementary Material.

## Supplementary Materials

The following supporting information can be downloaded at: https://www.mdpi.com/article/10.3390/ph15111395/s1; ^1^H and ^13^C NMR spectra of synthesized compounds.
