# Supplementary material for: Synthesis and Biological Evaluation of a Fused Structure of Indolizine and Pyrrolo[1,2-c]pyrimidine: Identification of Its Potent Anticancer Activity against Liver Cancer Cells
Source: Pharmaceuticals (Basel). 2022 Nov 12;15(11):1395. doi: 10.3390/ph15111395 (PMC9694334; doi:10.3390/ph15111395)

Supplementary Material

Synthesis and Biological Evaluation of a Fused Structure of Indolizine and Pyrrolo[1,2-*c*]pyrimidine: Identification of Its Potent Anticancer Activity Against Liver Cancer Cells

Seonghyeon Nam,[‡] Yechan Lee,[‡] So-Hyeon Park, Wan Namkung*, and Ikyon Kim*

College of Pharmacy and Yonsei Institute of Pharmaceutical Sciences, Yonsei University

85 Songdogwahak-ro, Yeonsu-gu, Incheon, 21983, Republic of Korea

* Corresponding authors.

Tel.: +82 32 749 4519; fax: +82 32 749 4105; e-mail: wnamkung@yonsei.ac.kr

Tel.: +82 32 749 4515; fax: +82 32 749 4105; e-mail: ikyonkim@yonsei.ac.kr

[‡] These authors contributed equally.

Table of Contents

Copies of ^{1}H and ^{13}C NMR spectra of **4** **S3**

Copies of ^{1}H and ^{13}C NMR spectra of **5** **S4-31**

Copies of ^{1}H and ^{13}C NMR spectra of **8** **S32**

Copies of ^{1}H and ^{13}C NMR spectra of **9** **S33**

Copies of ^{1}H and ^{13}C NMR spectra of **10** **S34-37**

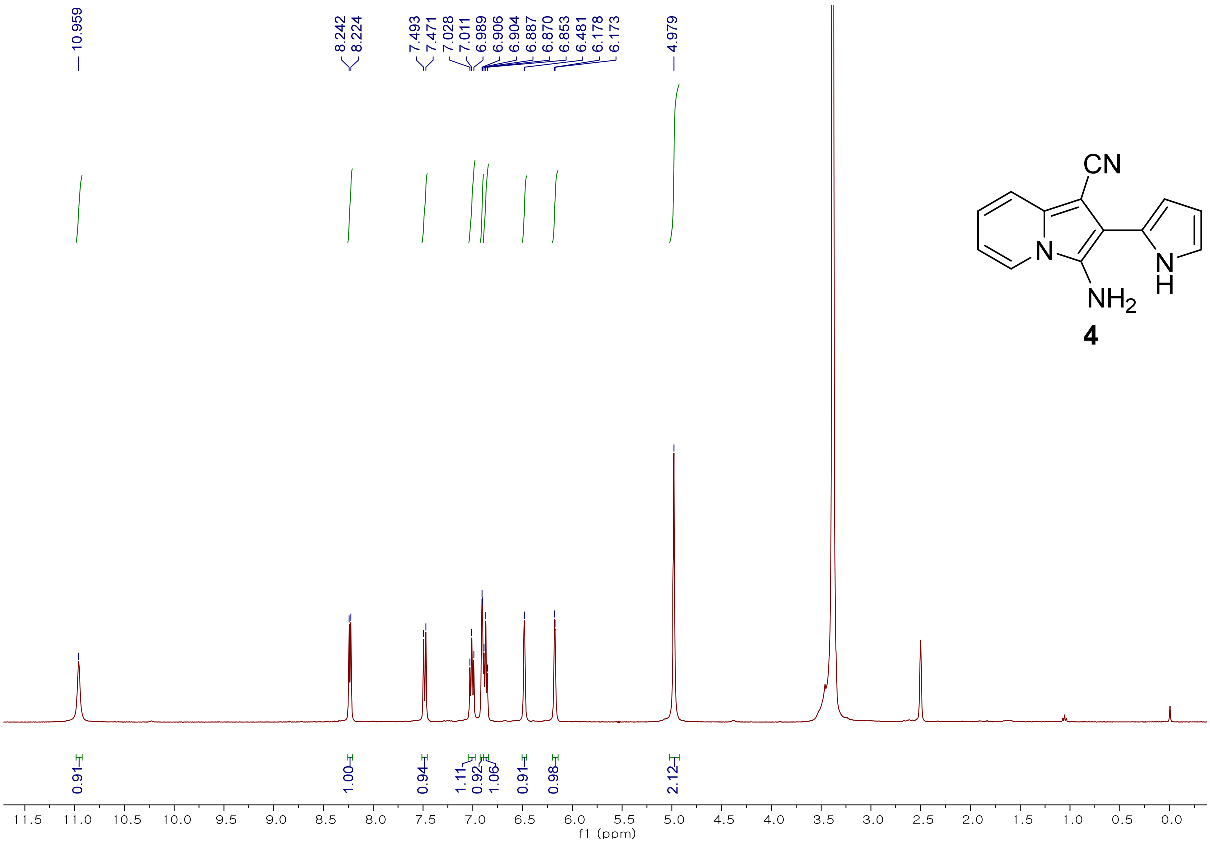
10.959
8.242
8.224
7.493
7.471
7.028
7.011
6.989
6.906
6.904
6.887
6.870
6.853
6.481
6.178
6.173
4.979
CN
N
NH2
N
H
4
0.91
1.00
0.94
1.11
0.92
1.06
0.91
0.98
2.12
f1 (ppm)

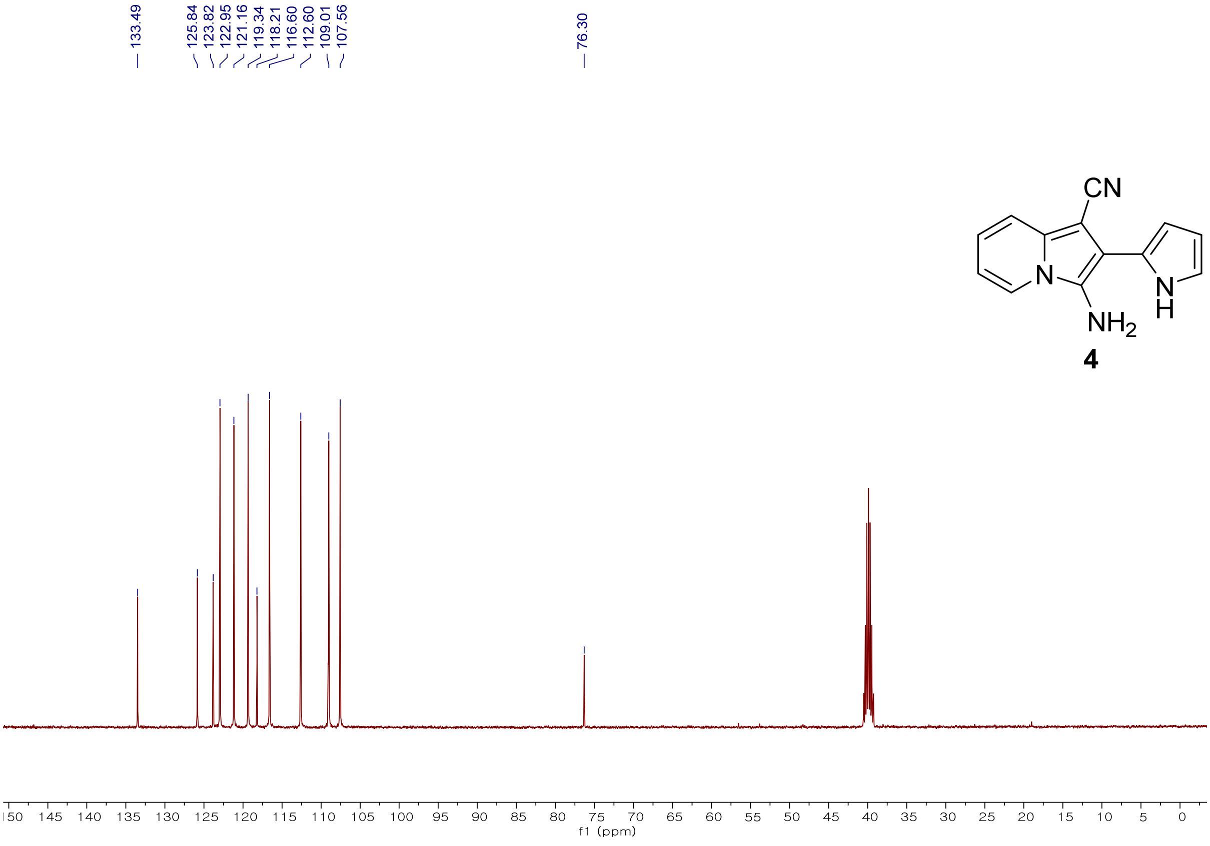
133.49
125.84
123.82
122.95
121.16
119.34
118.21
116.60
112.60
109.01
107.56
76.30
CN
N
NH2
N
H
4
f1 (ppm)

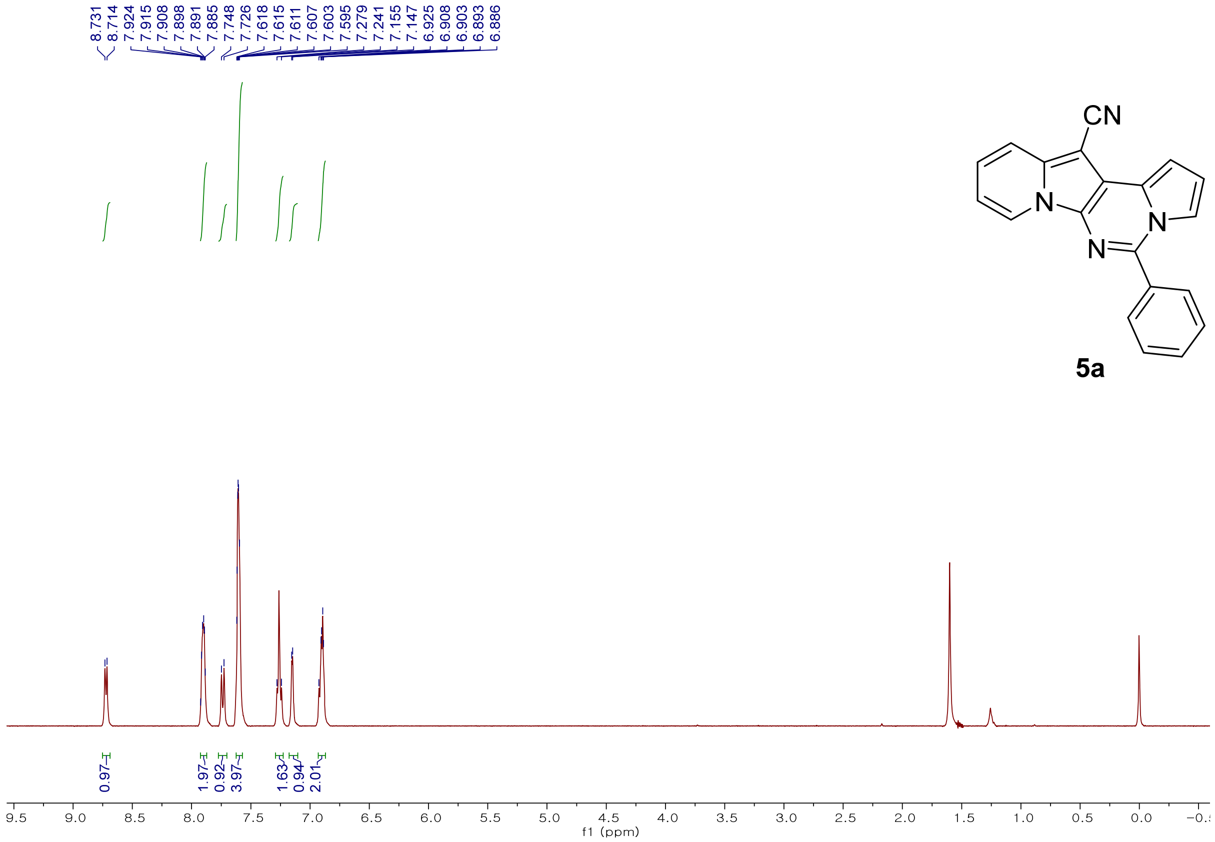
8.731
8.714
7.924
7.915
7.908
7.898
7.891
7.885
7.748
7.726
7.618
7.615
7.611
7.607
7.603
7.595
7.279
7.241
7.155
7.147
6.925
6.908
6.903
6.893
6.886
CN
N
N
N
5a
0.97
1.97
0.92
3.97
1.63
0.94
2.01
f1 (ppm)

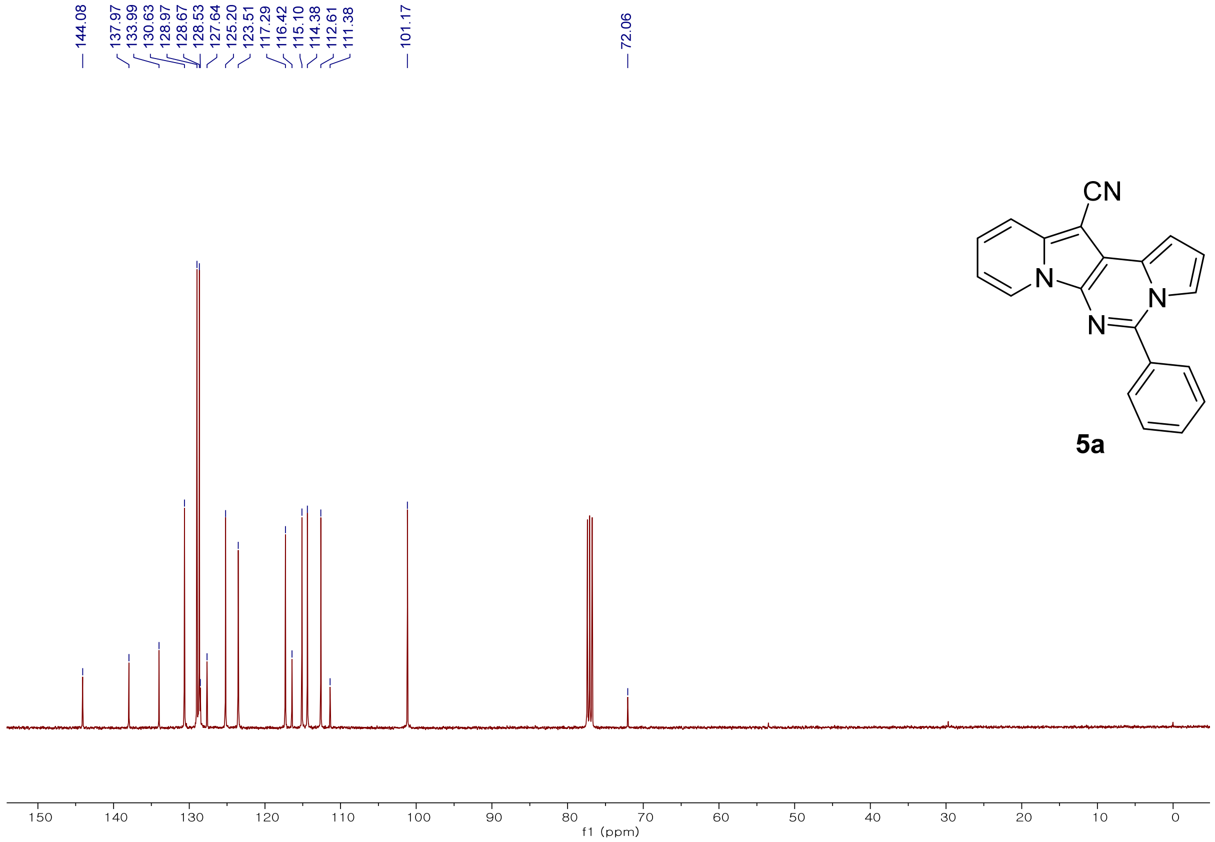
144.08
137.97
133.99
130.63
128.97
128.67
128.53
127.64
125.20
123.51
117.29
116.42
115.10
114.38
112.61
111.38
101.17
72.06
CN
N
N
N
5a
f1 (ppm)

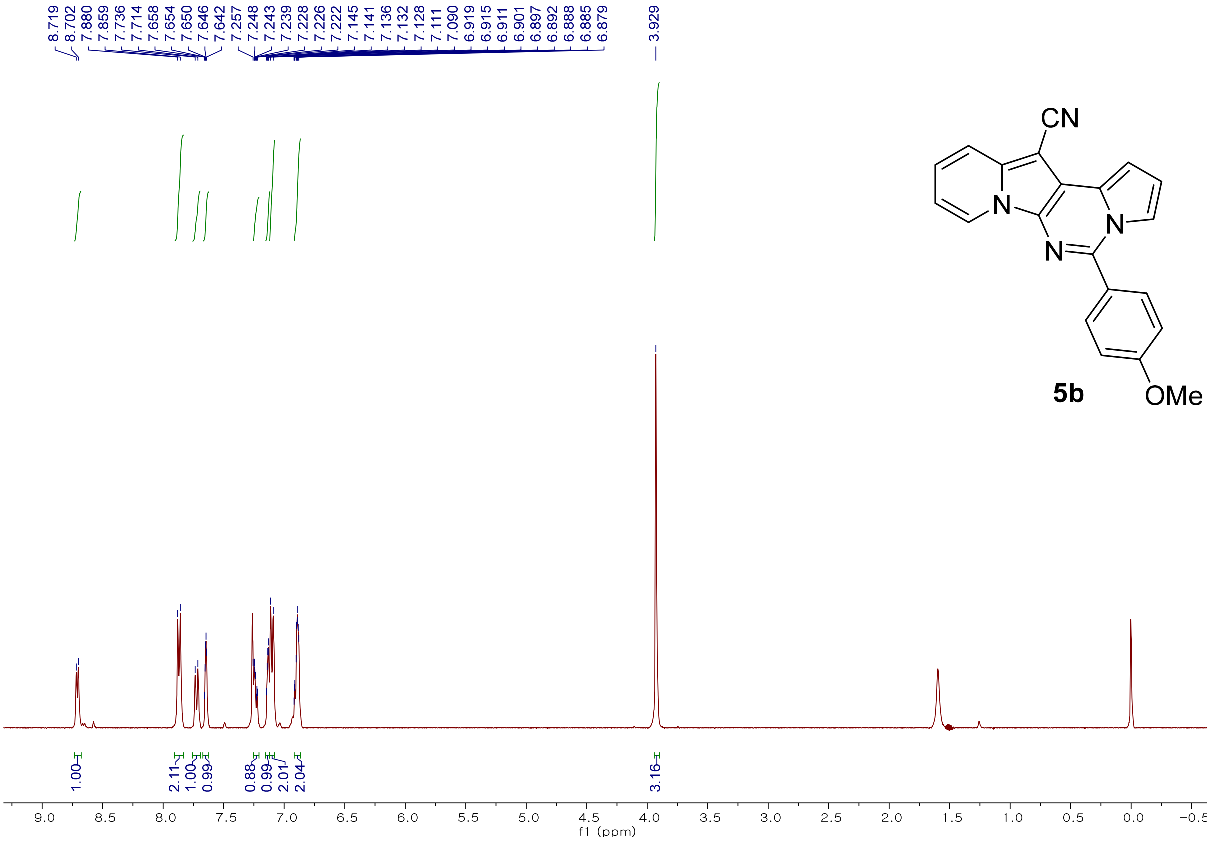
8.719
8.702
7.880
7.859
7.736
7.714
7.658
7.654
7.650
7.646
7.642
7.257
7.248
7.243
7.239
7.228
7.226
7.222
7.145
7.141
7.136
7.132
7.128
7.111
7.090
6.919
6.915
6.911
6.901
6.897
6.892
6.888
6.885
6.879
3.929
CN
N
N
N
5b
OMe
1.00
2.11
1.00
0.99
0.88
0.99
2.01
2.04
3.16
f1 (ppm)

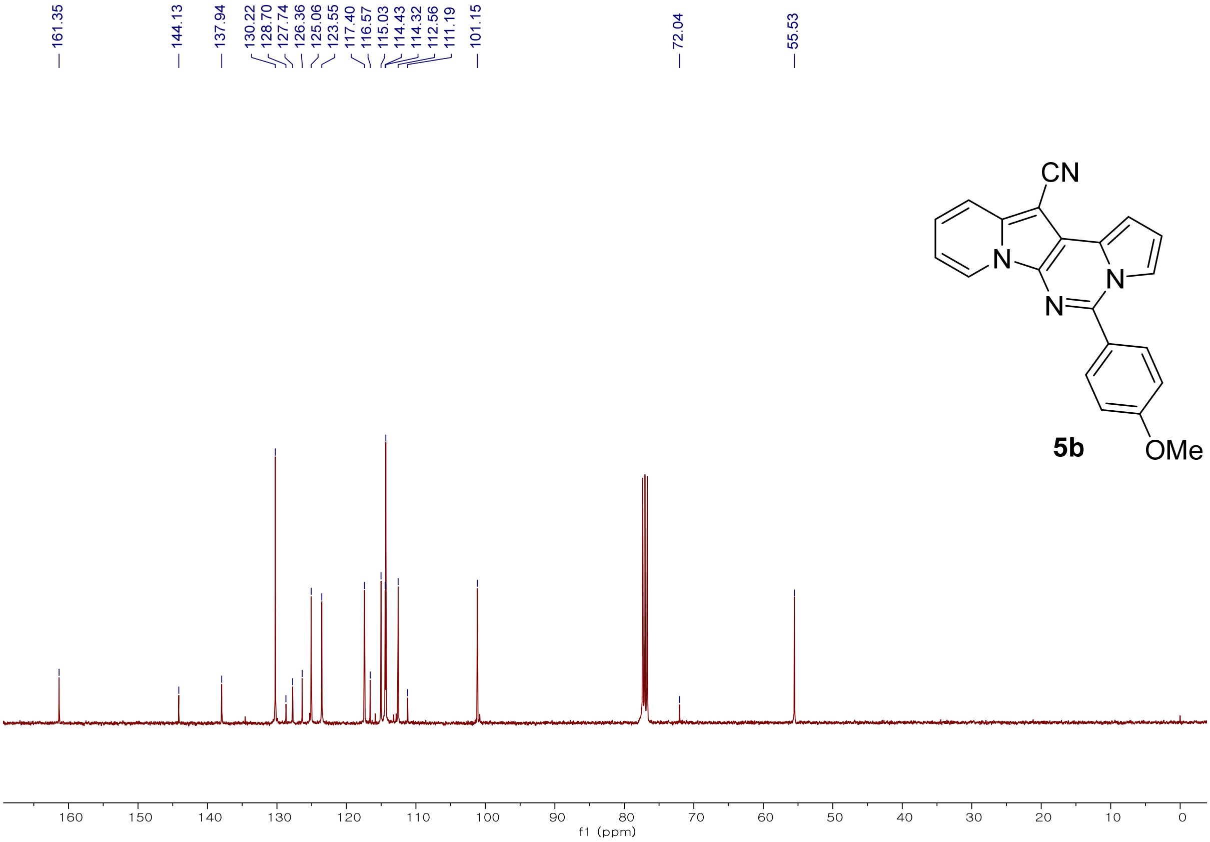
161.35
144.13
137.94
130.22
128.70
127.74
126.36
125.06
123.55
117.40
116.57
115.03
114.43
114.32
112.56
111.19
101.15
72.04
55.53
CN
N
N
N
5b
OMe
f1 (ppm)

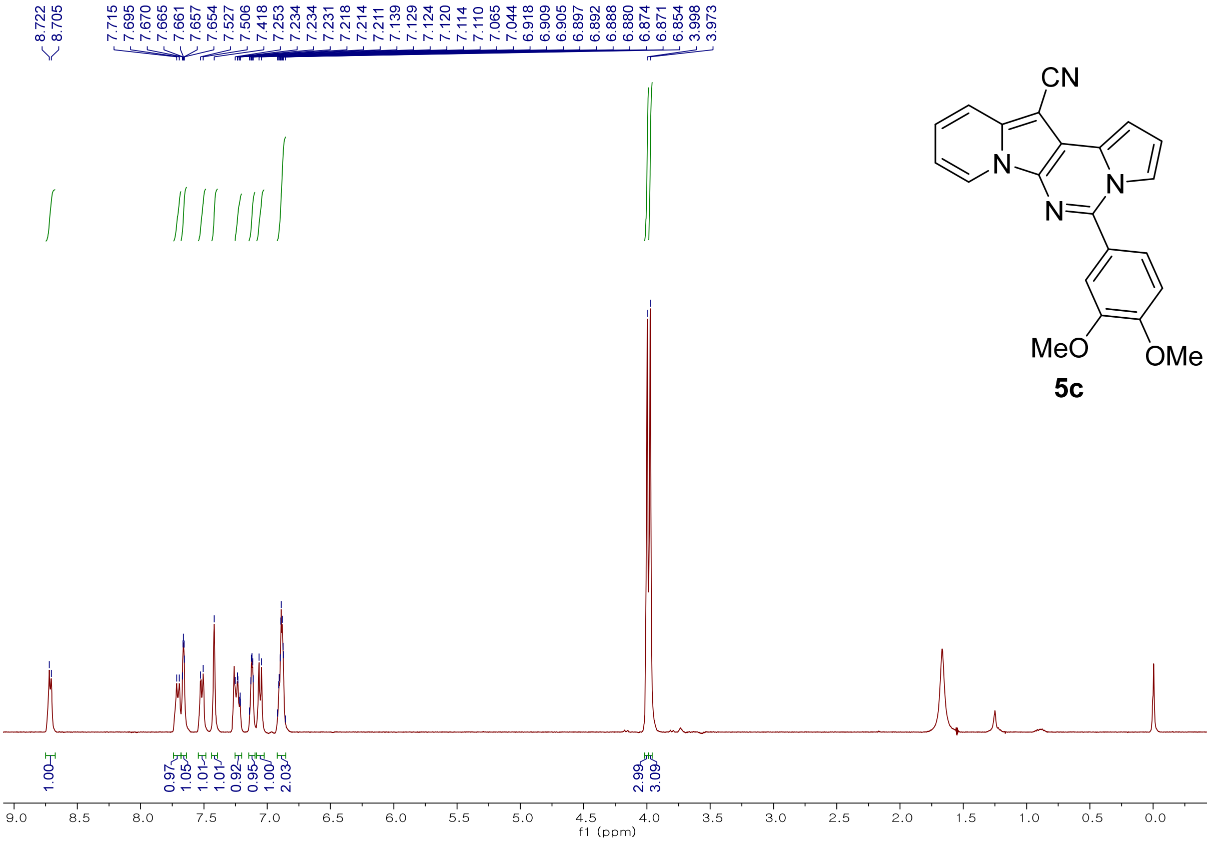
8.722
8.705
7.715
7.695
7.670
7.665
7.661
7.657
7.654
7.527
7.506
7.418
7.253
7.234
7.234
7.231
7.218
7.214
7.211
7.139
7.129
7.124
7.120
7.114
7.110
7.065
7.044
6.918
6.909
6.905
6.897
6.892
6.888
6.880
6.874
6.871
6.854
3.998
3.973
CN
N
N
N
MeO
OMe
1.00
0.97
1.05
1.01
1.01
0.92
0.95
1.00
2.03
2.99
3.09
f1 (ppm)

5c

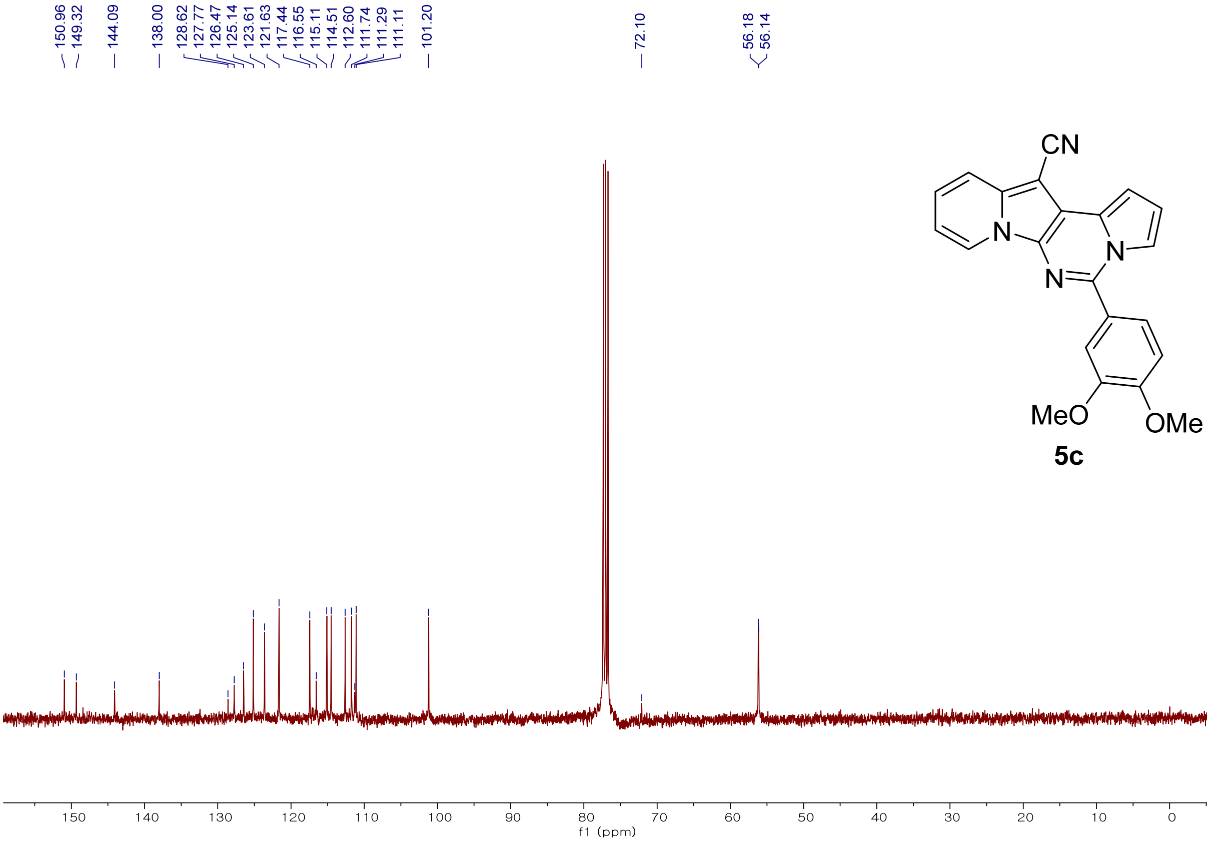
150.96
149.32
144.09
138.00
128.62
127.77
126.47
125.14
123.61
121.63
117.44
116.55
115.11
114.51
112.60
111.74
111.29
111.11
101.20
72.10
56.18
56.14
CN
N
N
N
MeO
OMe
f1 (ppm)

5c

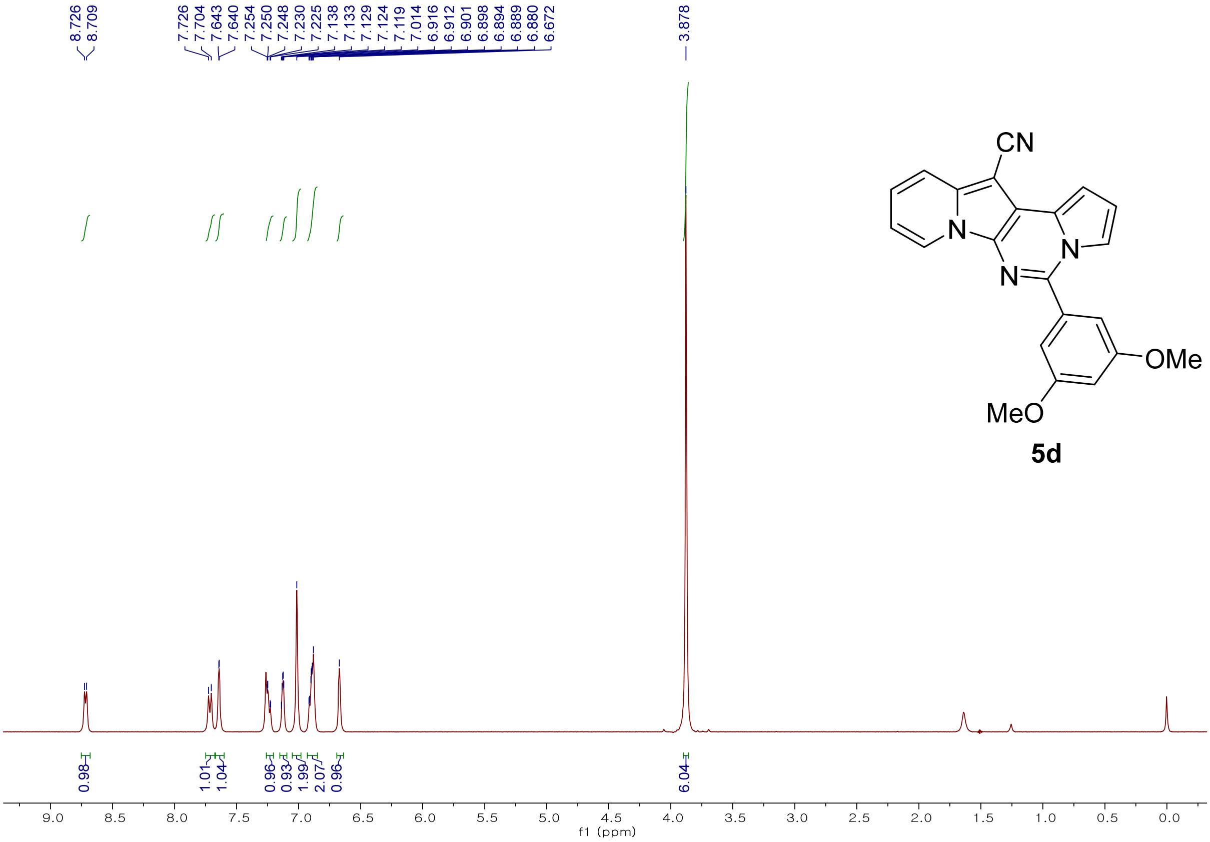
8.726
8.709
7.726
7.704
7.643
7.640
7.254
7.250
7.248
7.230
7.225
7.138
7.133
7.129
7.124
7.119
7.014
6.916
6.912
6.901
6.898
6.894
6.889
6.880
6.672
3.878
CN
N
N
N
OMe
MeO
5d
0.98
1.01
1.04
0.96
0.93
1.99
2.07
0.96
6.04
f1 (ppm)

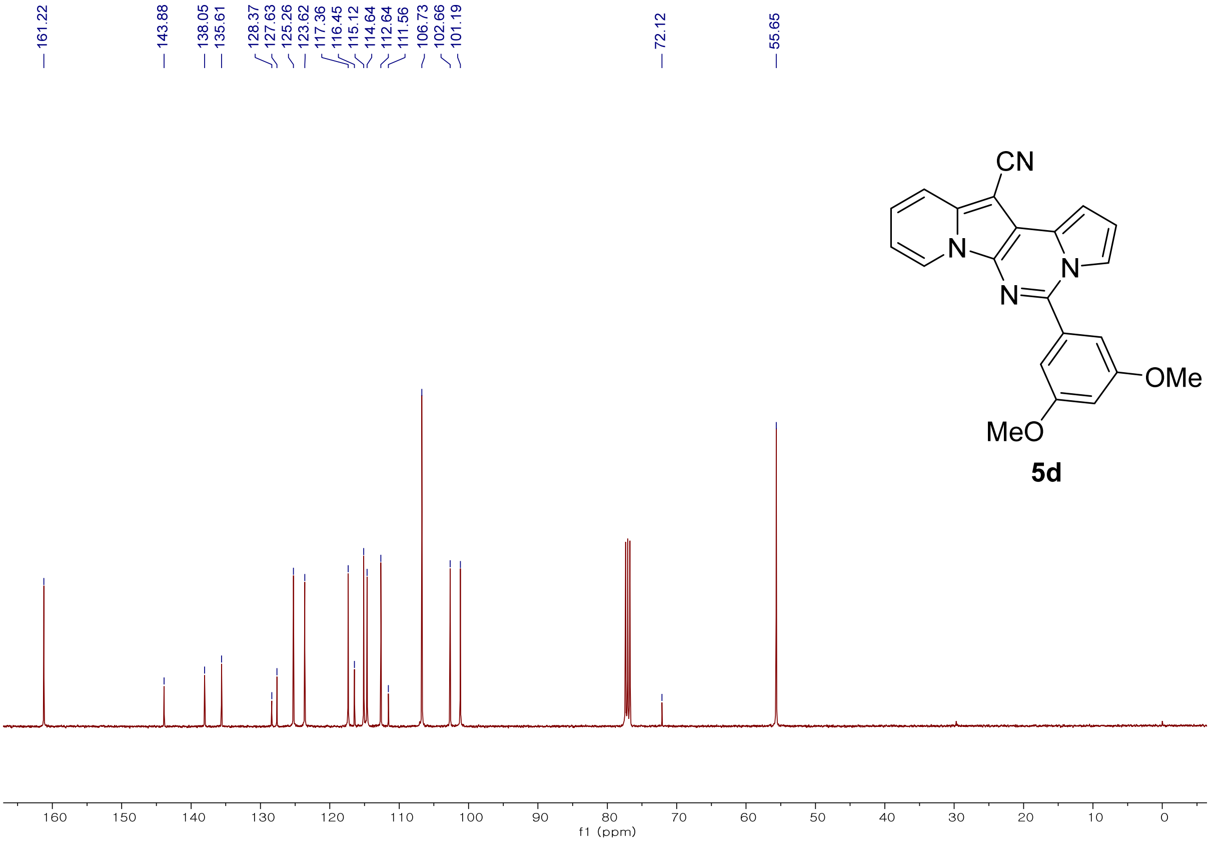
161.22
143.88
138.05
135.61
128.37
127.63
125.26
123.62
117.36
116.45
115.12
114.64
112.64
111.56
106.73
102.66
101.19
72.12
55.65
CN
N
N
N
OMe
MeO
5d
f1 (ppm)

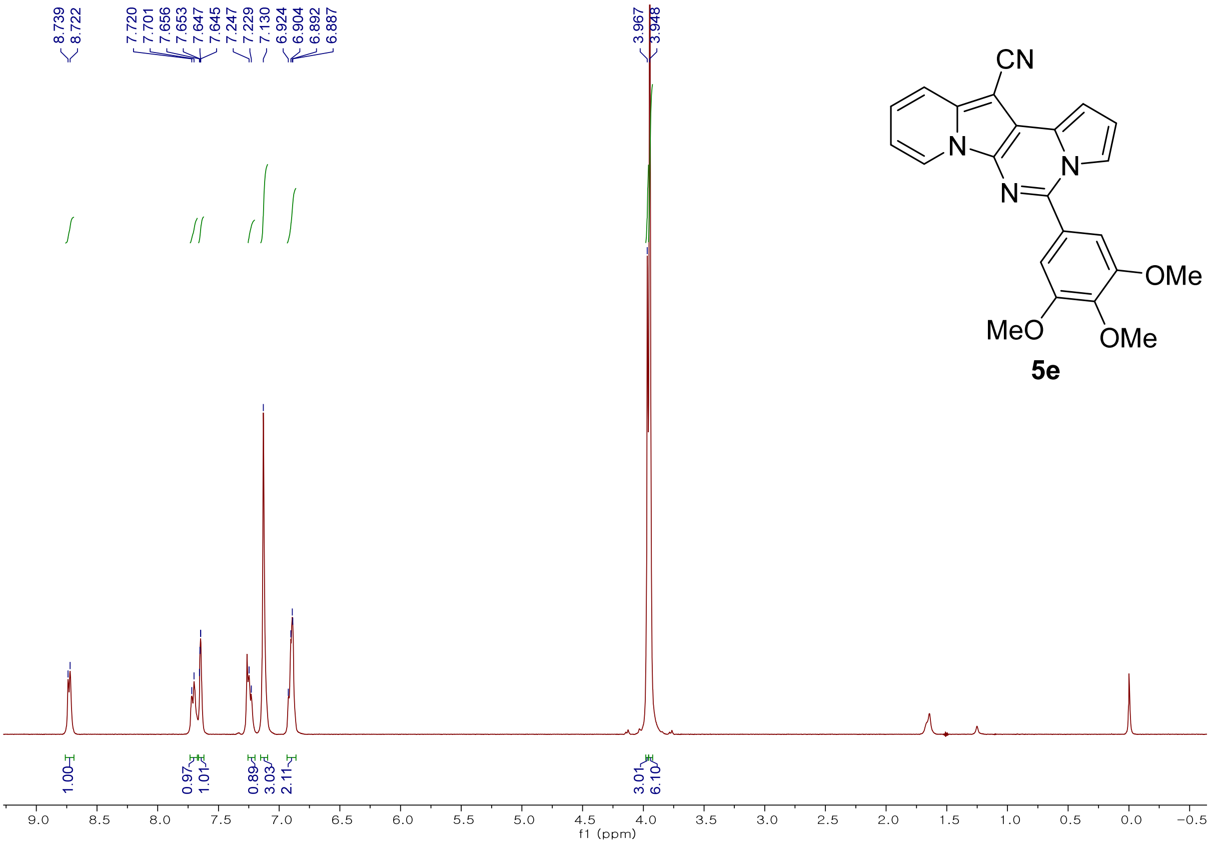
8.739
8.722
7.720
7.701
7.656
7.653
7.647
7.645
7.247
7.229
7.130
6.924
6.904
6.892
6.887
3.967
3.948
CN
N
N
N
OMe
MeO
OMe
5e
1.00
0.97
1.01
0.89
3.03
2.11
3.01
6.10
9.0
8.5
8.0
7.5
7.0
6.5
6.0
5.5
5.0
4.5
4.0
3.5
3.0
2.5
2.0
1.5
1.0
0.5
0.0
−0.5
f1 (ppm)

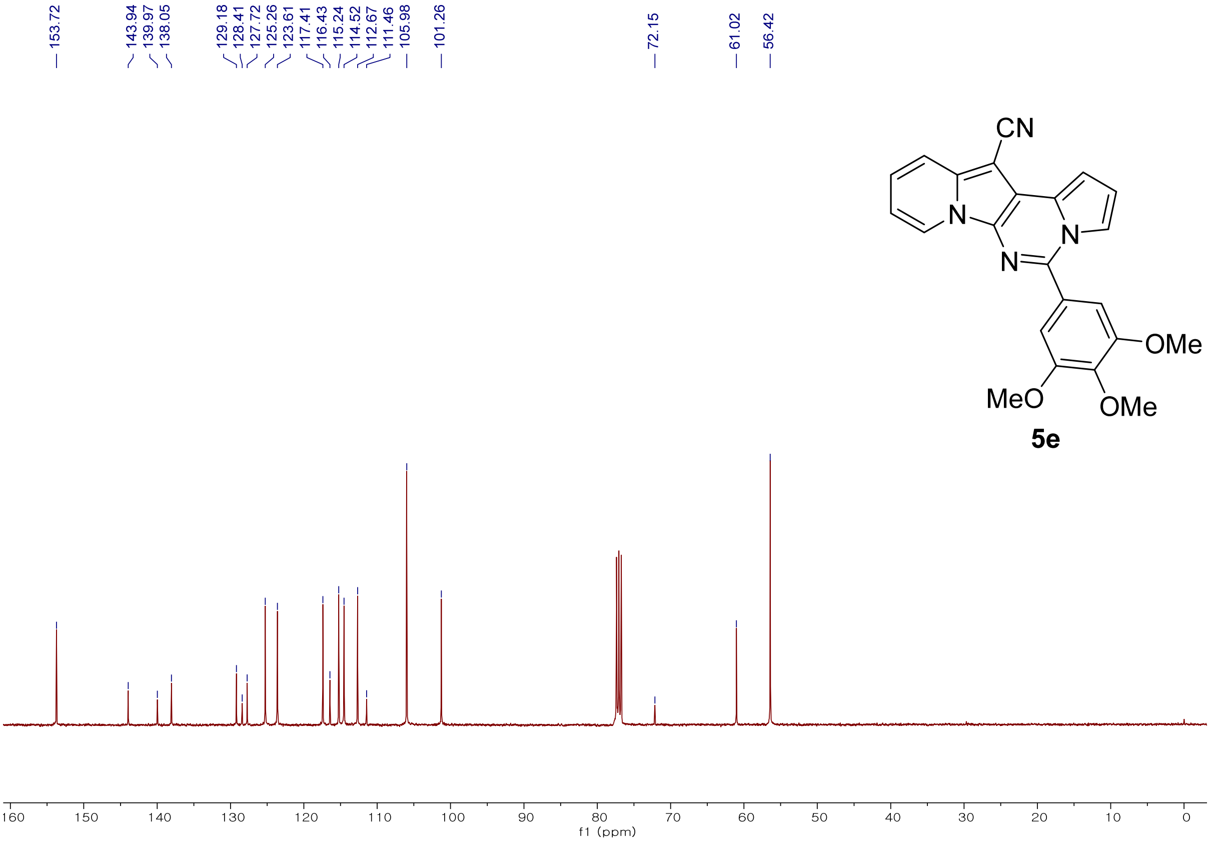
153.72
143.94
139.97
138.05
129.18
128.41
127.72
125.26
123.61
117.41
116.43
115.24
114.52
112.67
111.46
105.98
101.26
72.15
61.02
56.42
CN
N
N
N
OMe
MeO
OMe
5e
160
150
140
130
120
110
100
90
80
70
60
50
40
30
20
10
0
f1 (ppm)

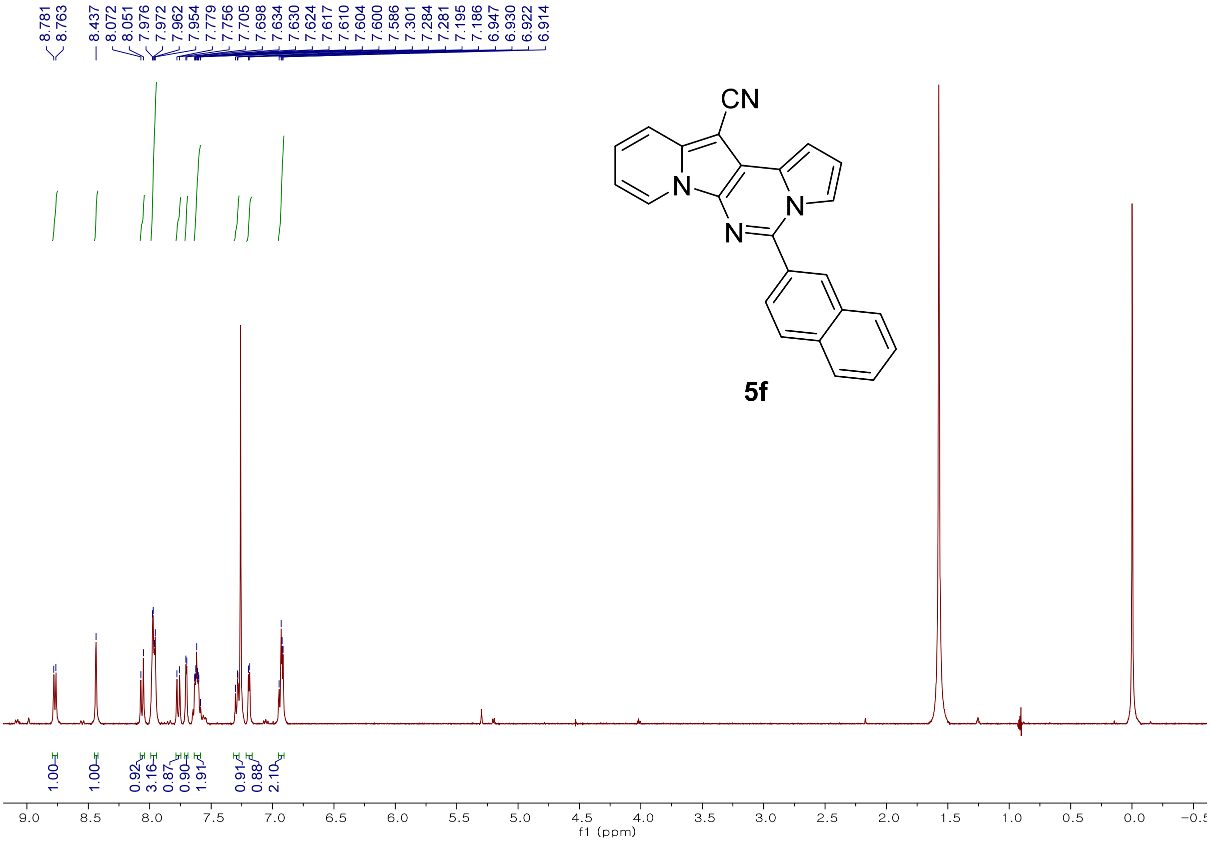

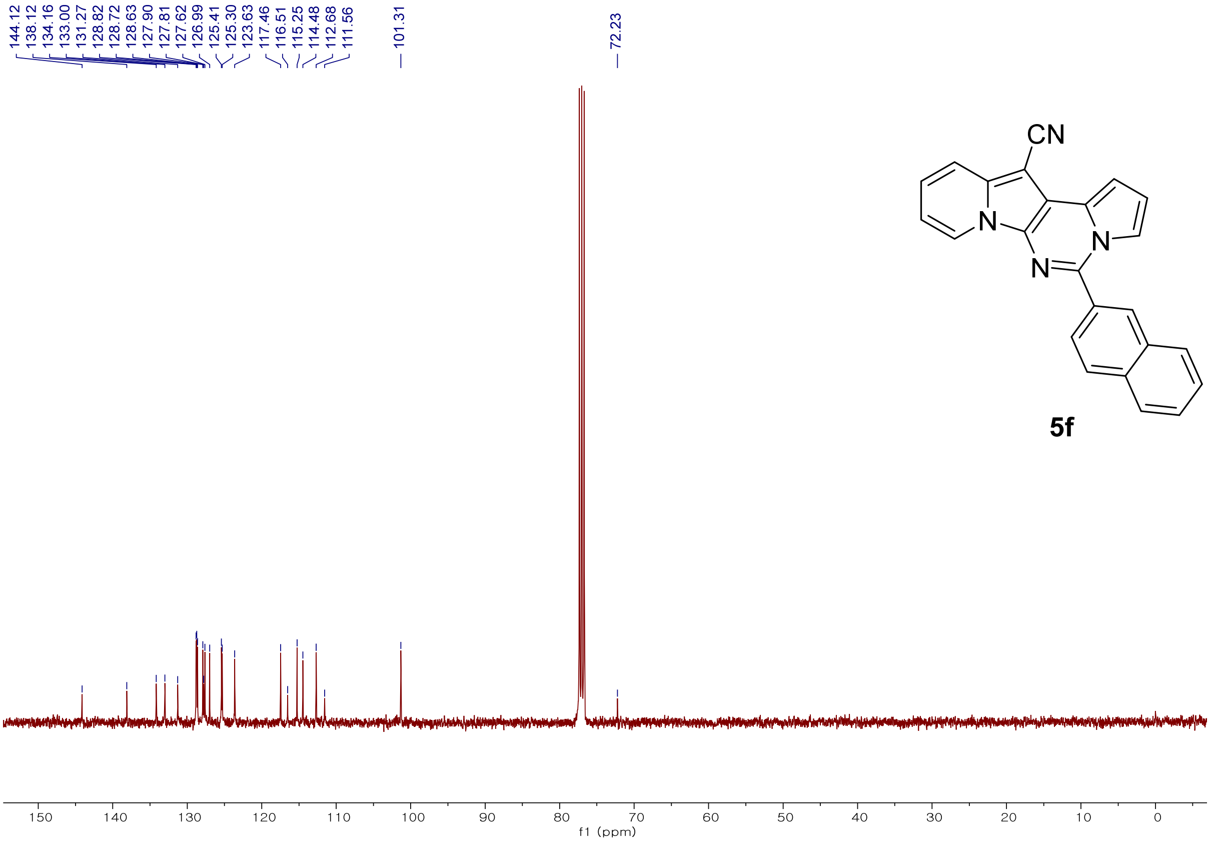

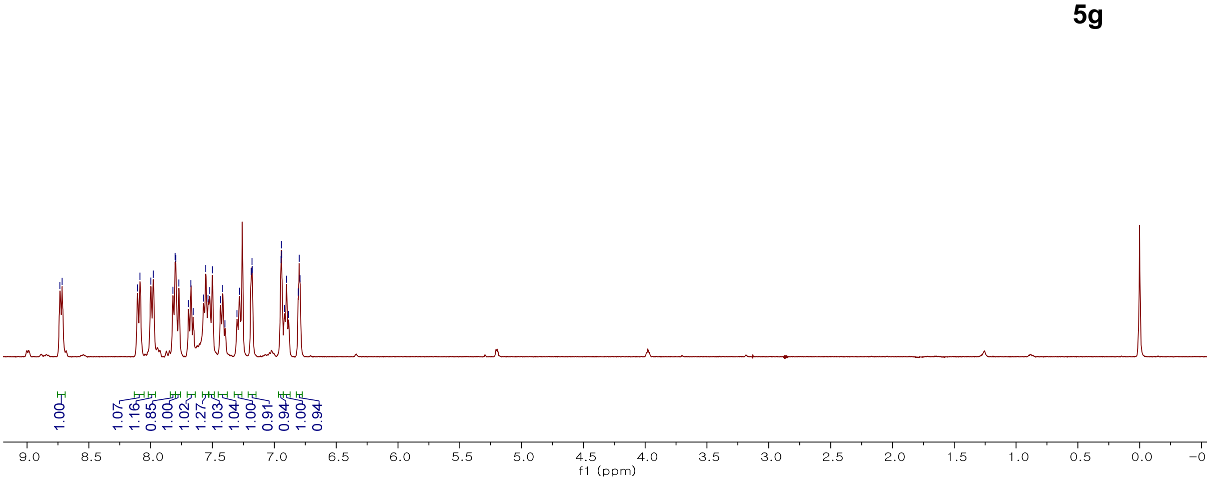
8.734
8.717
8.107
8.087
7.999
7.979
7.820
7.802
7.797
7.773
7.695
7.675
7.656
7.572
7.555
7.537
7.523
7.501
7.435
7.417
7.399
7.302
7.282
7.187
7.181
7.179
6.947
6.943
6.940
6.918
6.901
6.885
6.807
6.799
6.792
1.00
1.07
1.16
0.85
1.00
1.02
1.27
1.03
1.04
1.00
0.91
0.94
1.00
0.94
f1 (ppm)

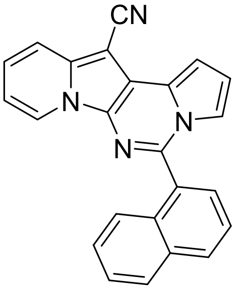
CN
N
N
N

5g

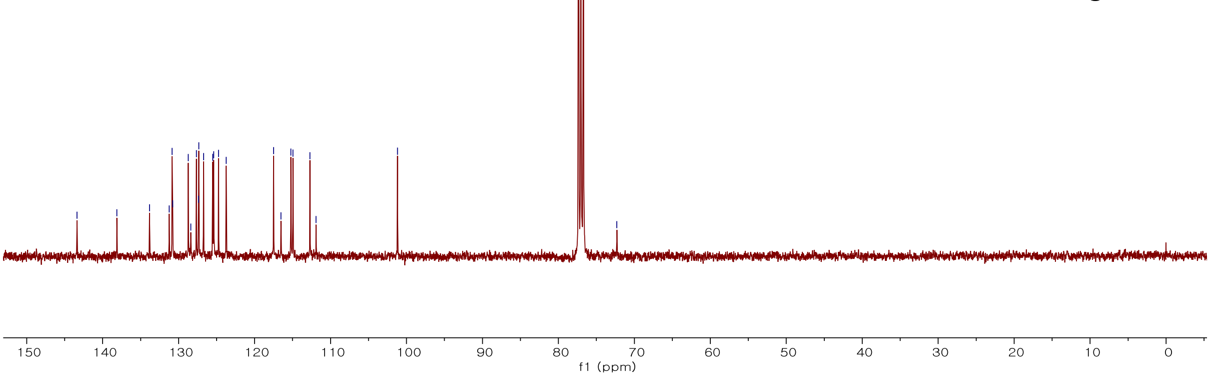
143.38
138.13
133.83
131.23
130.87
130.78
128.73
128.40
127.66
127.38
127.34
126.72
125.51
125.37
124.74
123.74
117.49
116.52
115.21
114.95
112.71
111.91
101.18
72.30
f1 (ppm)

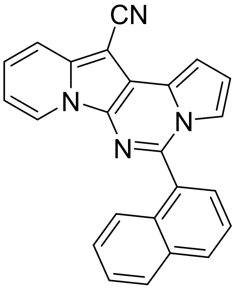
CN
N
N
N

5g

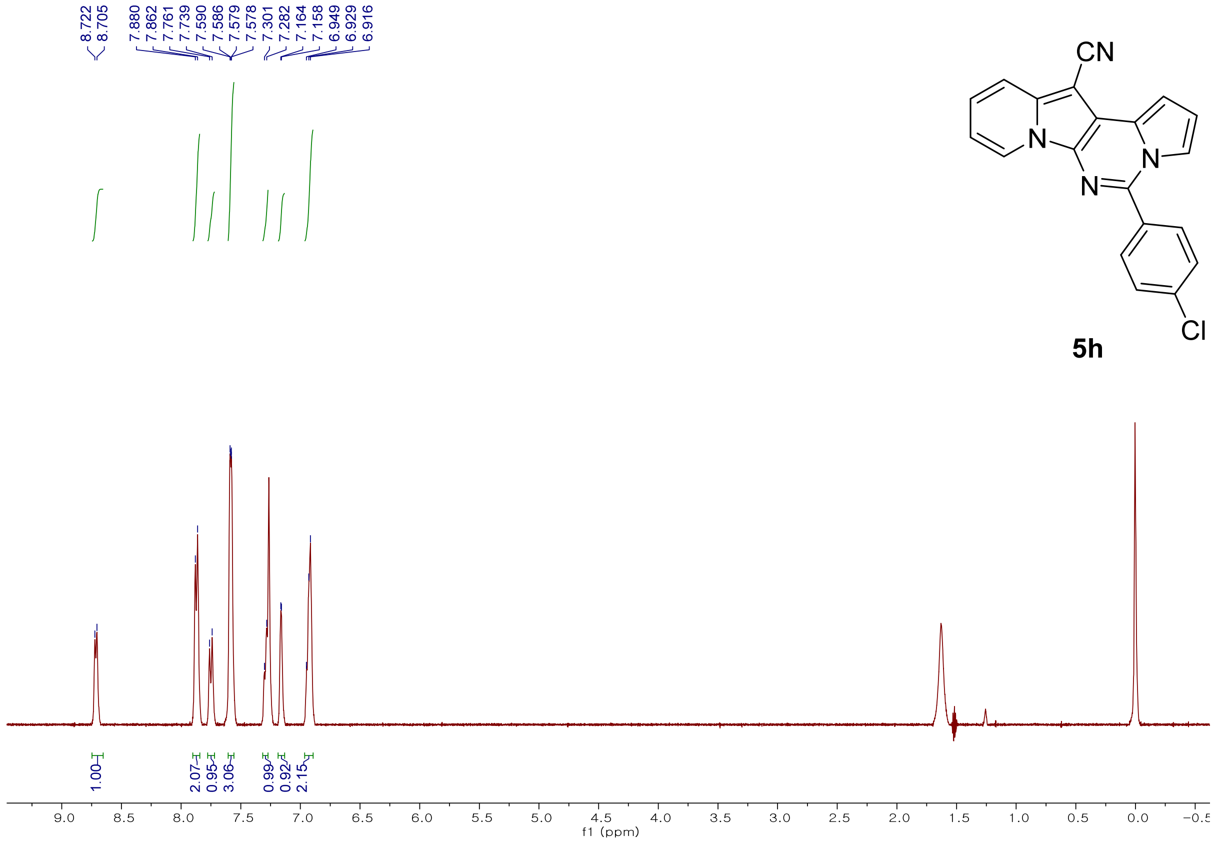
8.722
8.705
7.880
7.862
7.761
7.739
7.590
7.586
7.579
7.578
7.301
7.282
7.164
7.158
6.949
6.929
6.916
1.00
2.07
0.95
3.06
0.99
0.92
2.15
f1 (ppm)
CN
N
N
N
Cl

5h

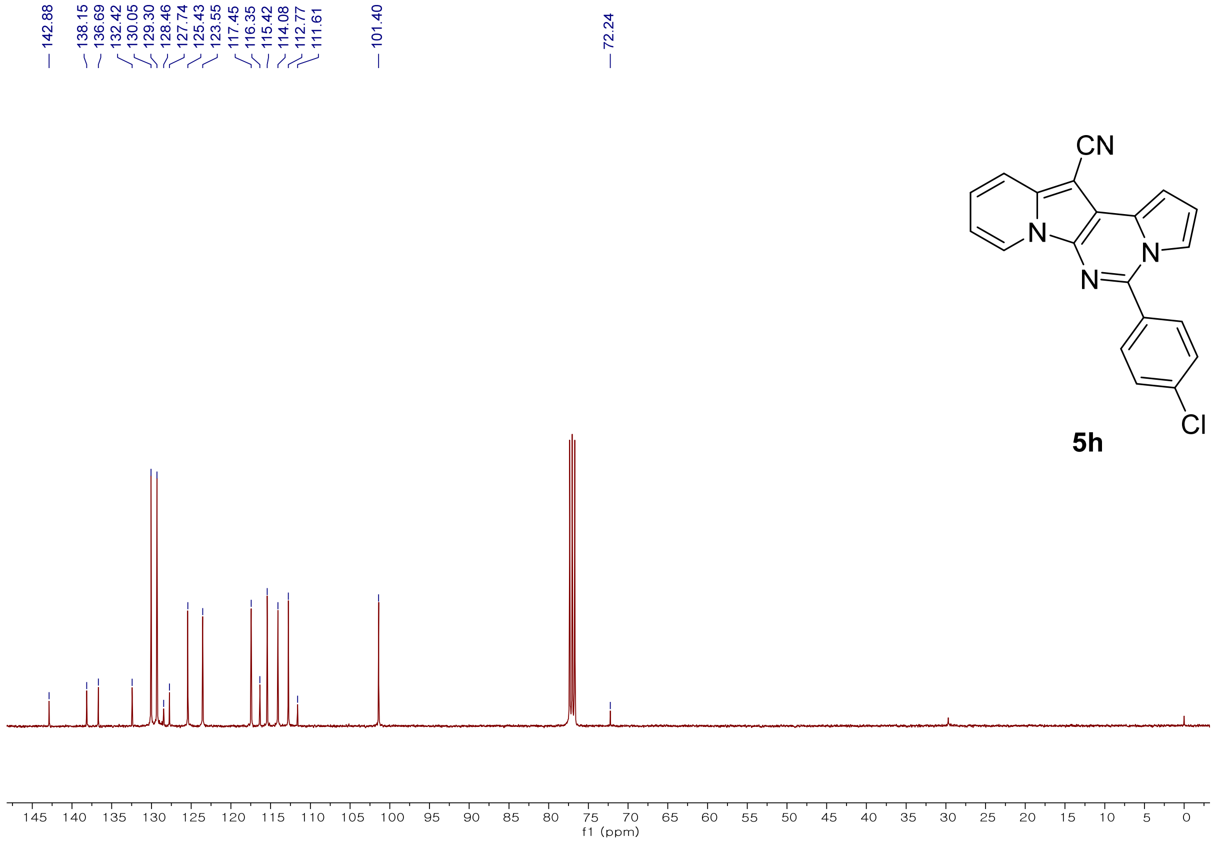
142.88
138.15
136.69
132.42
130.05
129.30
128.46
127.74
125.43
123.55
117.45
116.35
115.42
114.08
112.77
111.61
101.40
72.24
f1 (ppm)
CN
N
N
N
Cl

5h

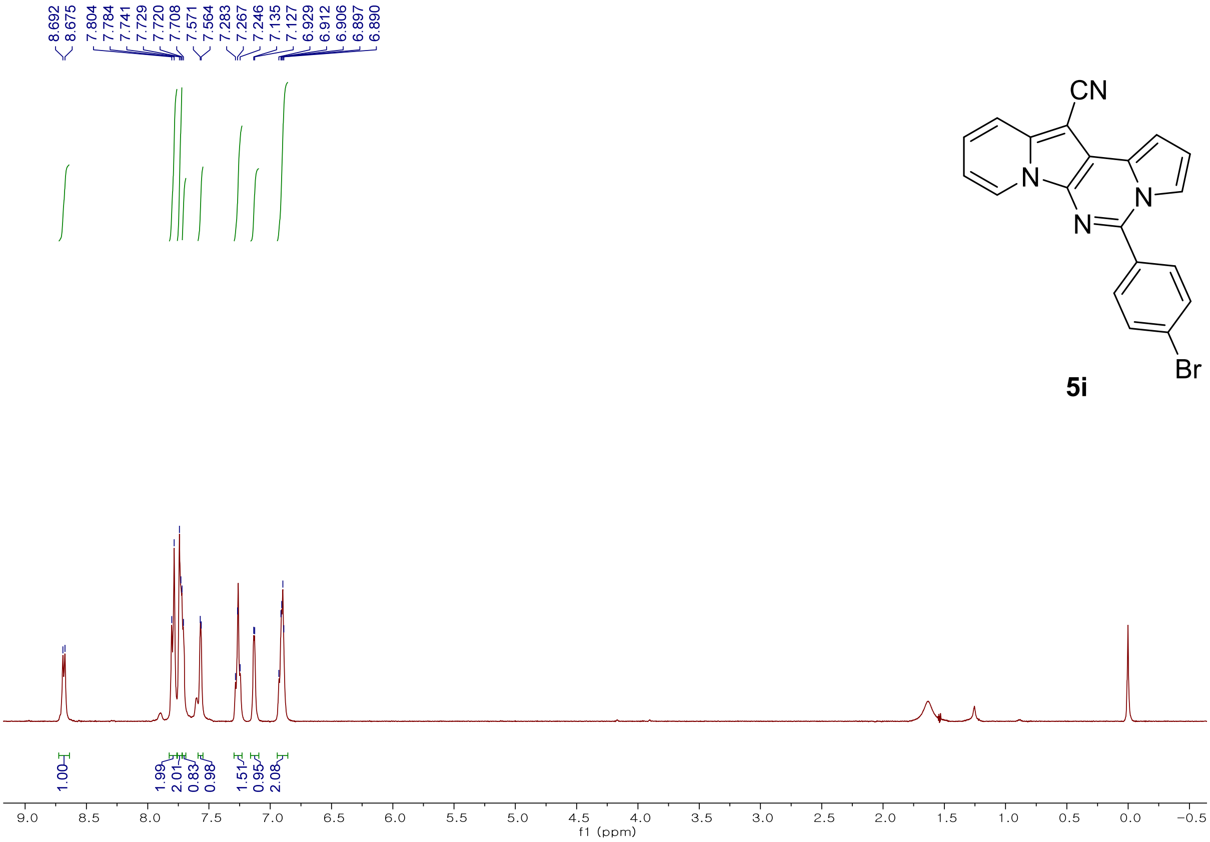
8.692
8.675
7.804
7.784
7.741
7.729
7.720
7.708
7.571
7.564
7.283
7.267
7.246
7.135
7.127
6.929
6.912
6.906
6.897
6.890
1.00
1.99
2.01
0.83
0.98
1.51
0.95
2.08
f1 (ppm)
CN
N
N
N
Br

5i

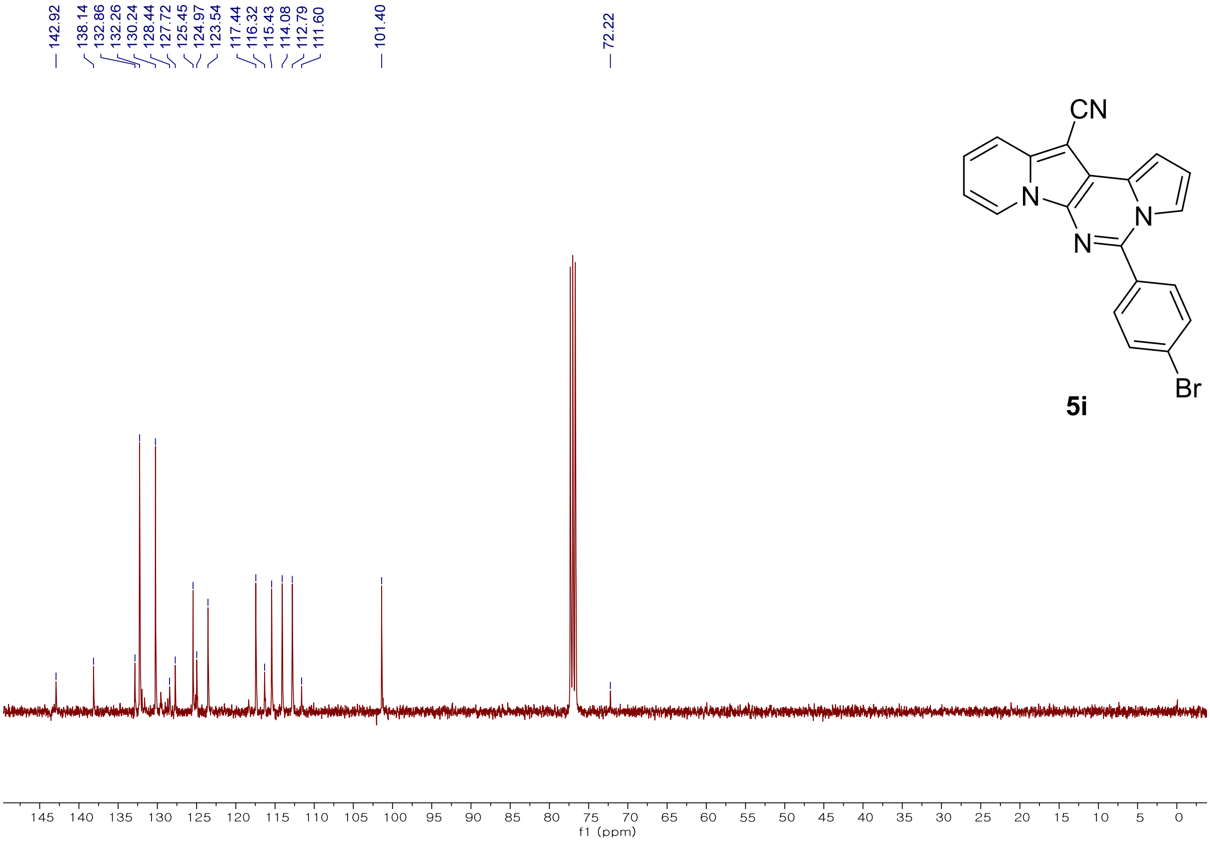
142.92
138.14
132.86
132.26
130.24
128.44
127.72
125.45
124.97
123.54
117.44
116.32
115.43
114.08
112.79
111.60
101.40
72.22
f1 (ppm)
CN
N
N
N
Br

5i

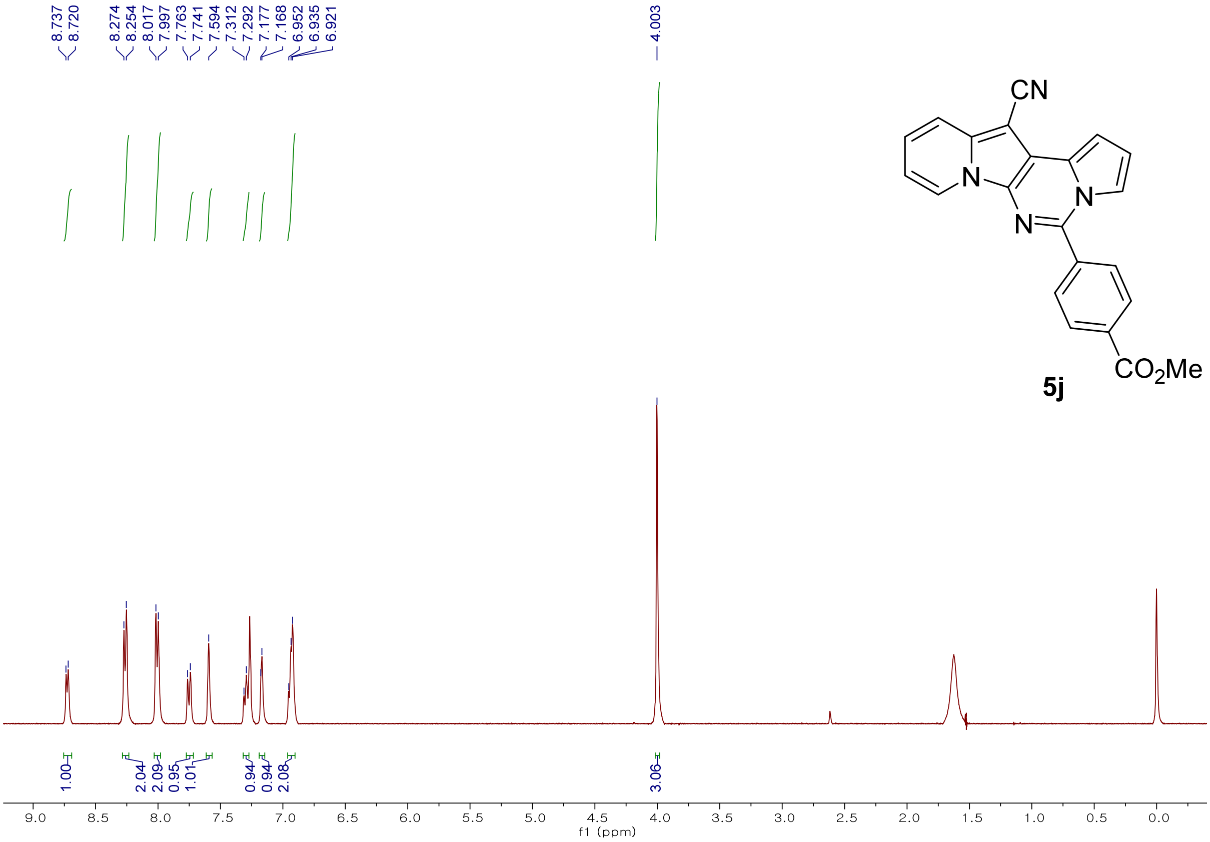
8.737
8.720
8.274
8.254
8.017
7.997
7.763
7.741
7.594
7.312
7.292
7.177
7.168
6.952
6.935
6.921
4.003
CN
N
N
N
CO2Me
1.00
2.04
2.09
0.95
1.01
0.94
0.94
2.08
3.06
f1 (ppm)

5j

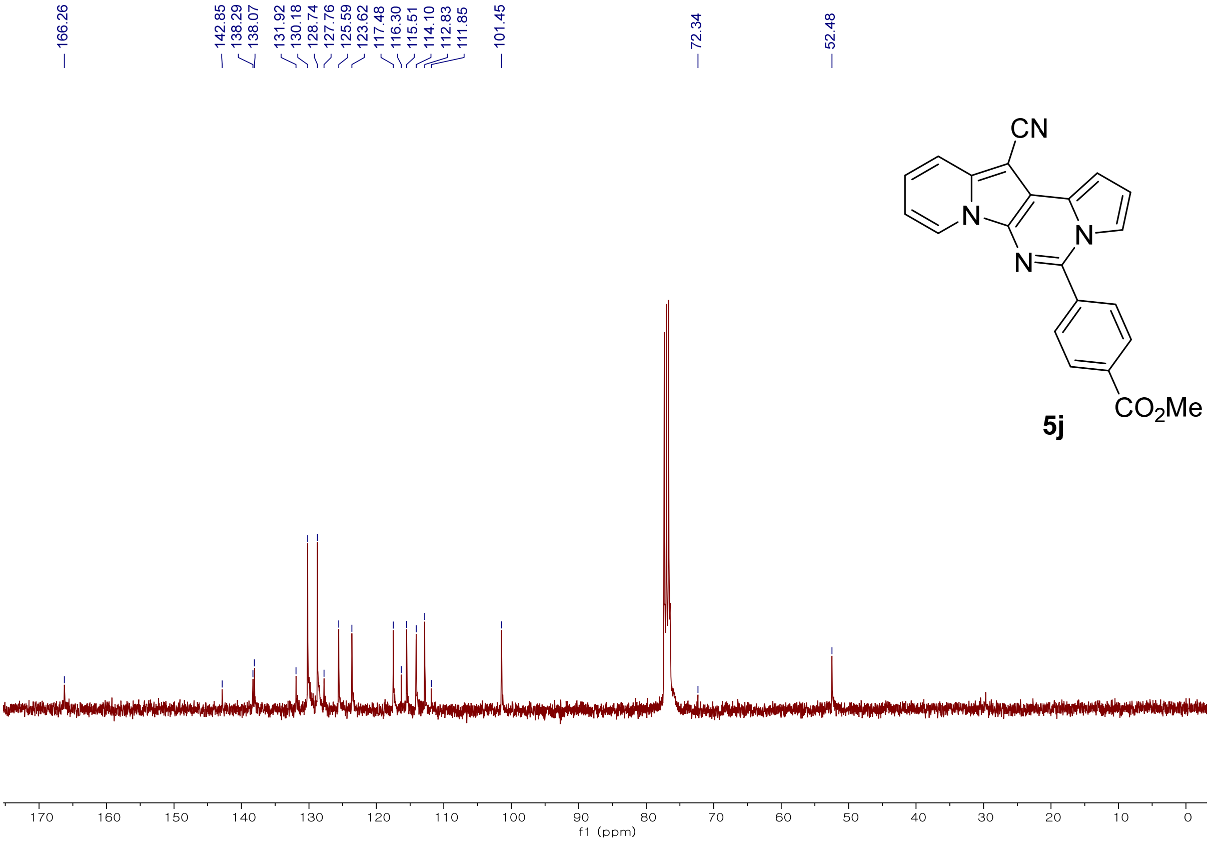
166.26
142.85
138.29
138.07
131.92
130.18
128.74
127.76
125.59
123.62
117.48
116.30
115.51
114.10
112.83
111.85
101.45
72.34
52.48
CN
N
N
N
CO2Me
f1 (ppm)

5j

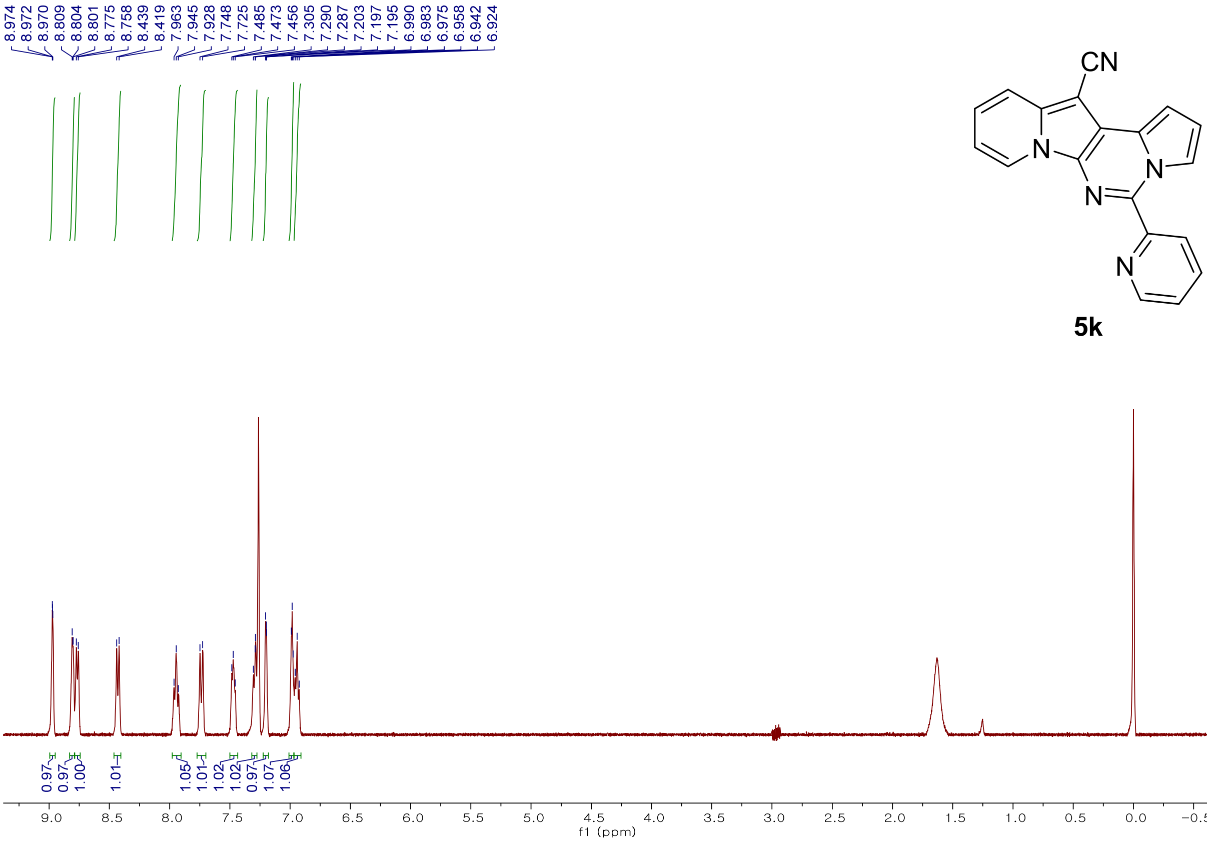
8.974
8.972
8.970
8.809
8.804
8.801
8.775
8.758
8.439
8.419
7.963
7.945
7.928
7.748
7.725
7.485
7.473
7.456
7.305
7.290
7.287
7.203
7.197
7.195
6.990
6.983
6.975
6.958
6.942
6.924
CN
N
N
N
N
5k
0.97
0.97
1.00
1.01
1.05
1.01
1.02
1.02
0.97
1.07
1.06
f1 (ppm)

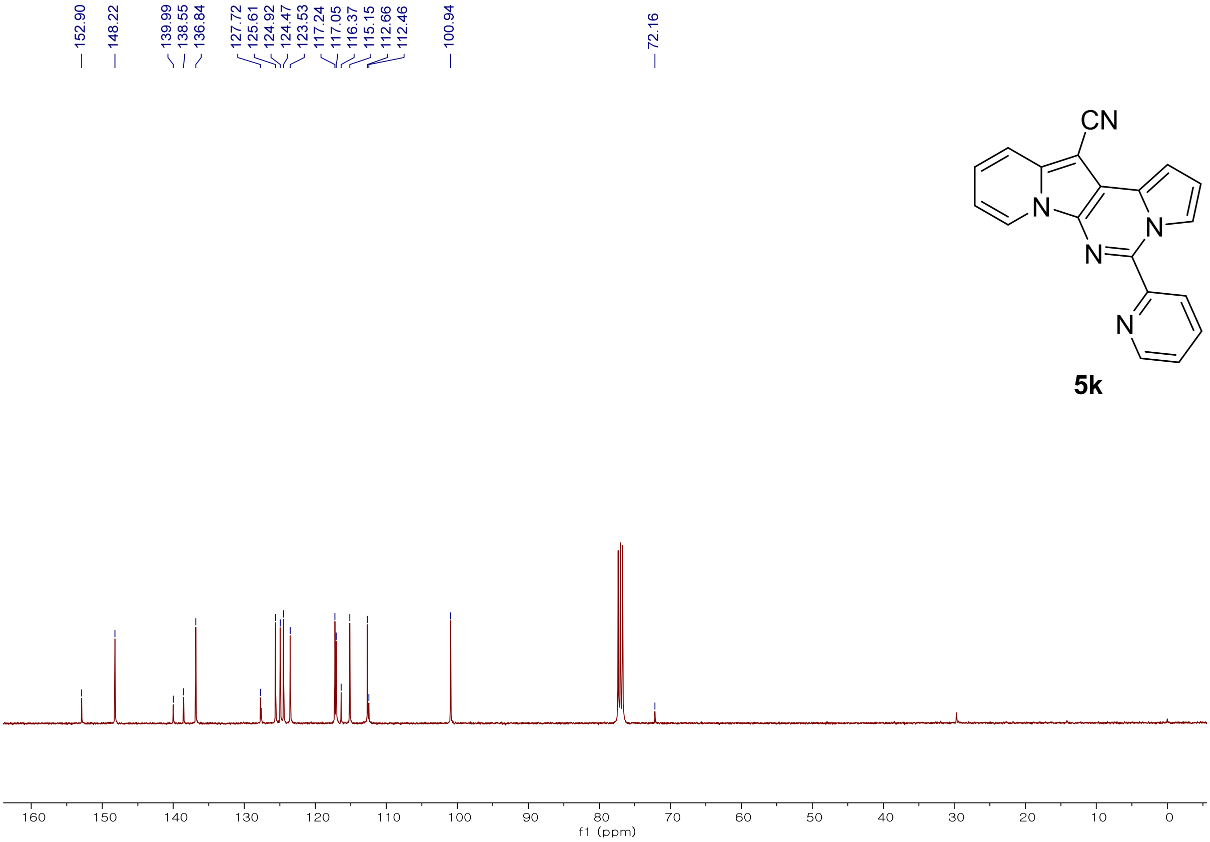
152.90
148.22
139.99
138.55
136.84
127.72
125.61
124.92
124.47
123.53
117.24
117.05
116.37
115.15
112.66
112.46
100.94
72.16
CN
N
N
N
N
5k
f1 (ppm)

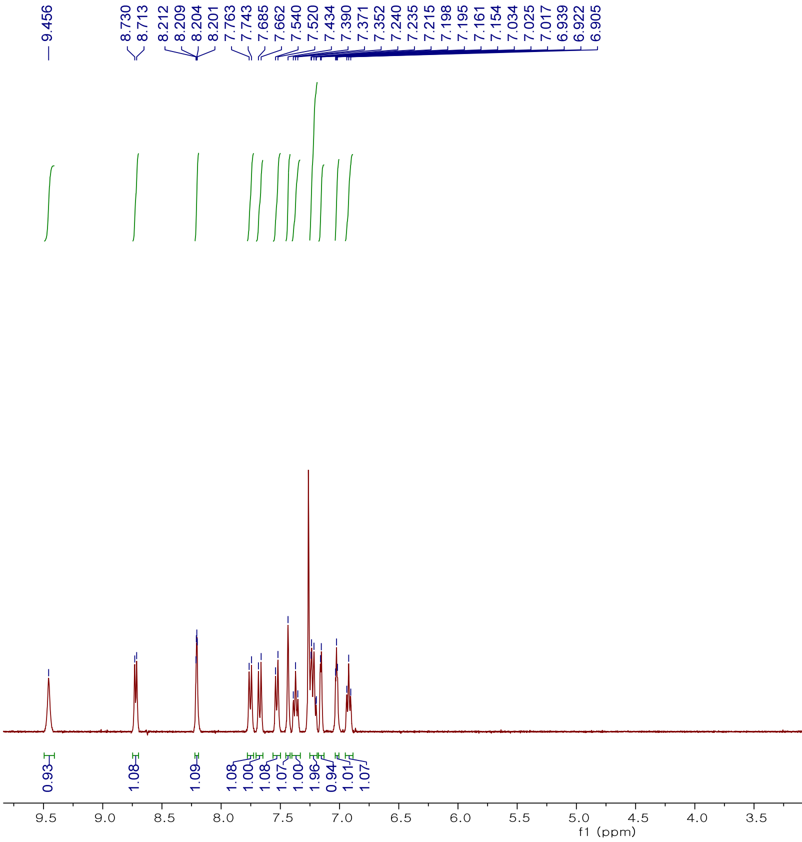

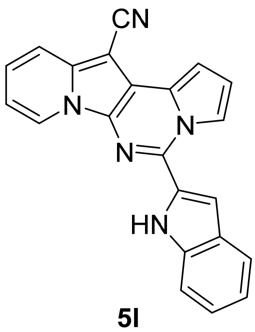

5l

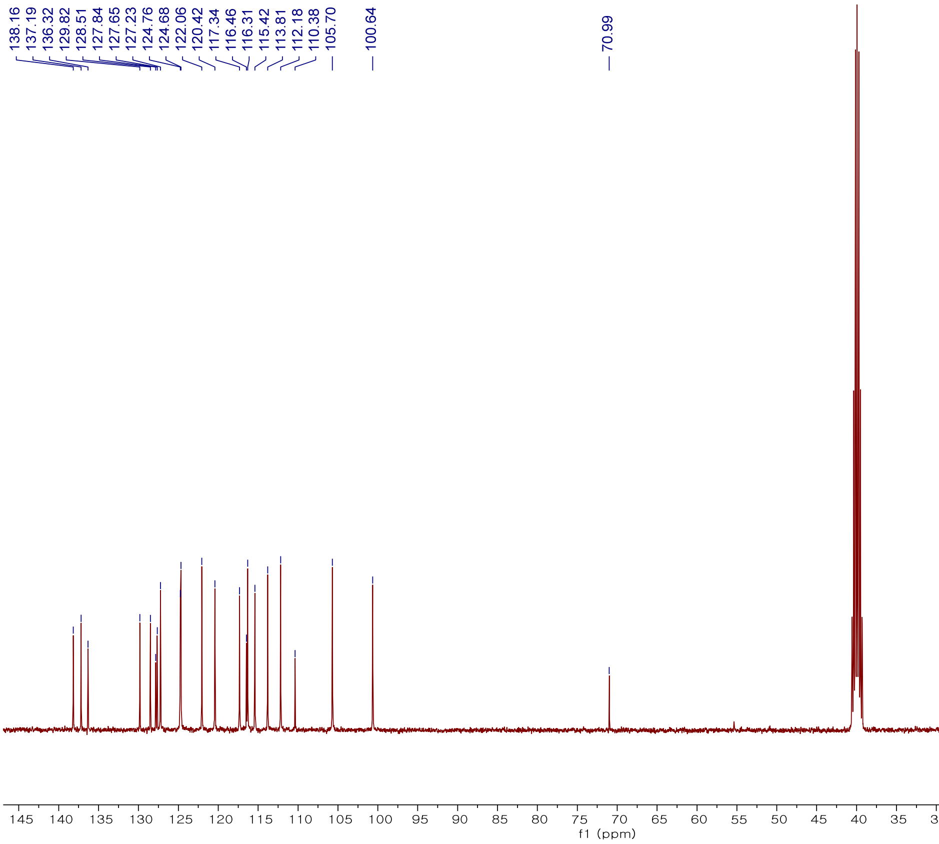

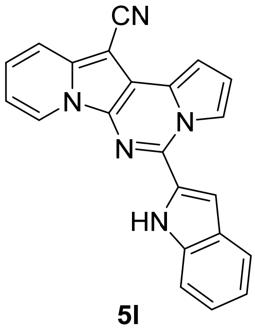

5l

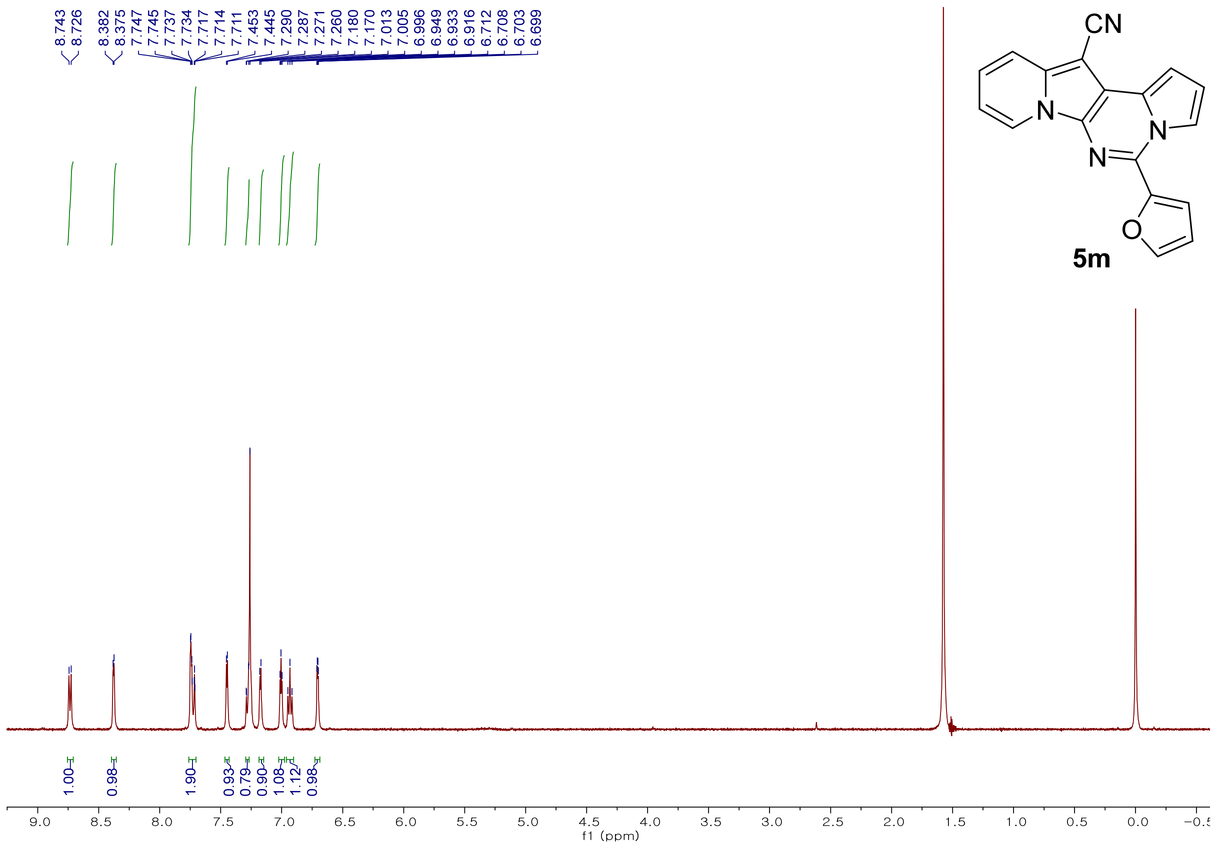
8.743
8.726
8.382
8.375
7.747
7.745
7.737
7.734
7.717
7.714
7.711
7.453
7.445
7.290
7.287
7.271
7.260
7.180
7.170
7.013
7.005
6.996
6.949
6.933
6.916
6.712
6.708
6.703
6.699
CN
N
N
N
O
5m
1.00
0.98
1.90
0.93
0.79
0.90
1.08
1.12
0.98
f1 (ppm)

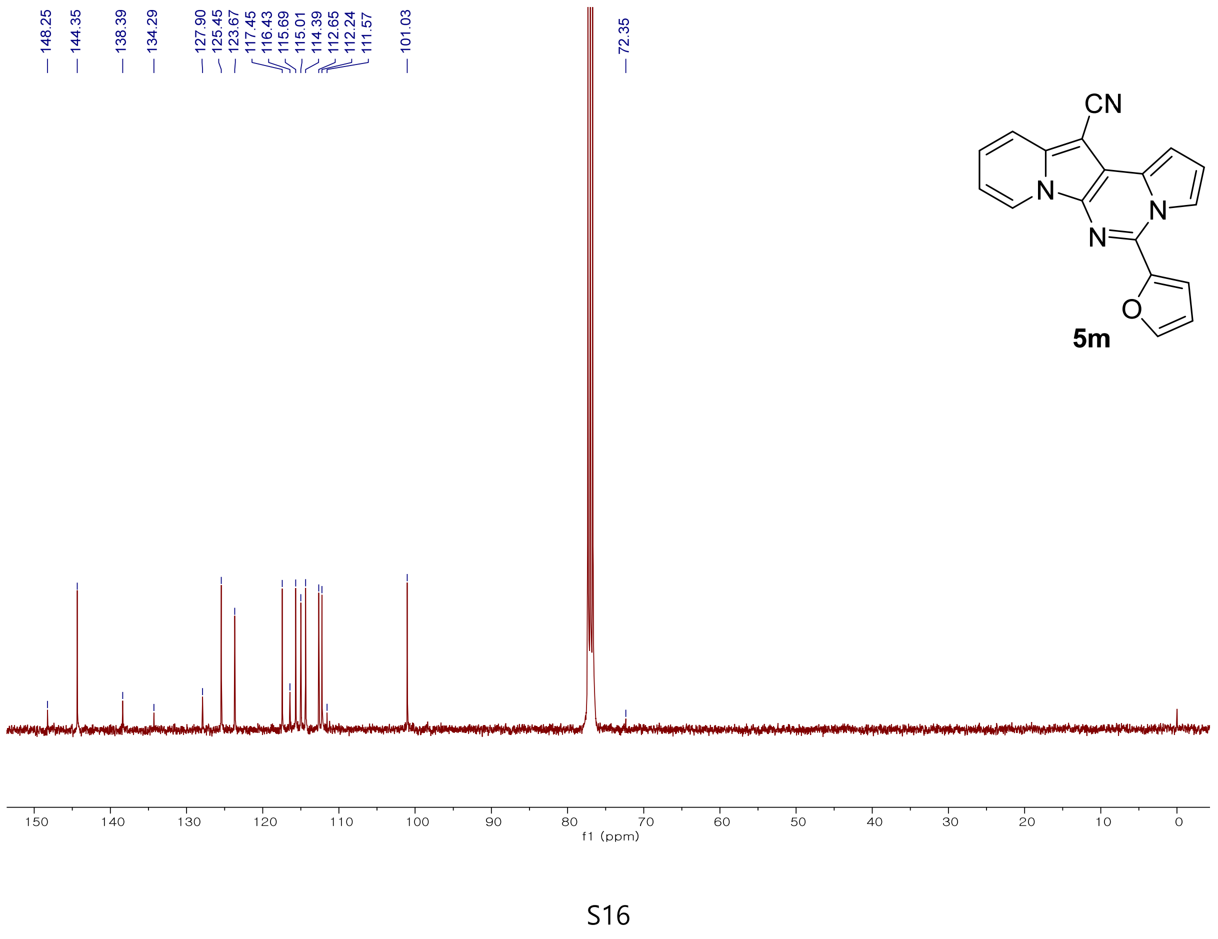
148.25
144.35
138.39
134.29
127.90
125.45
123.67
117.45
116.43
115.69
115.01
114.39
112.65
112.24
111.57
101.03
72.35
CN
N
N
N
O
5m
f1 (ppm)

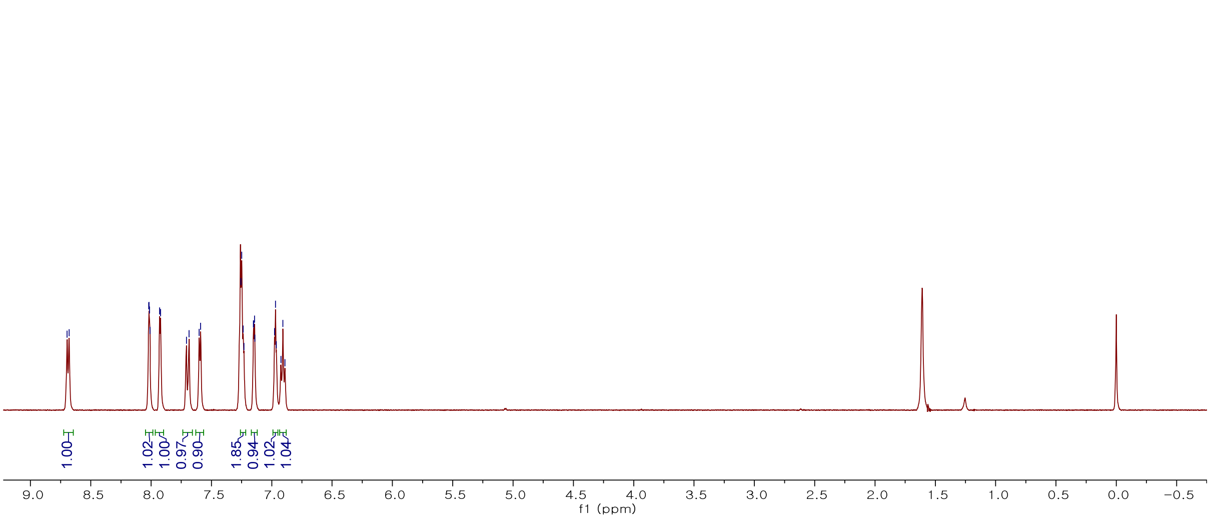
8.696
8.679
8.020
8.018
8.013
8.010
7.930
7.921
7.707
7.685
7.602
7.589
7.255
7.249
7.237
7.230
7.152
7.149
7.143
7.140
6.977
6.969
6.962
6.925
6.908
6.890
1.00
1.02
1.00
0.97
0.90
1.85
0.94
1.02
1.04
9.0 8.5 8.0 7.5 7.0 6.5 6.0 5.5 5.0 4.5 4.0 3.5 3.0 2.5 2.0 1.5 1.0 0.5 0.0 −0.5
f1 (ppm)

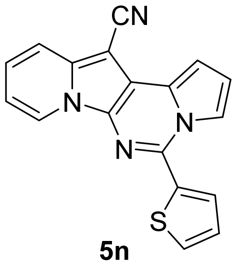
CN
N
N
N
S

5n

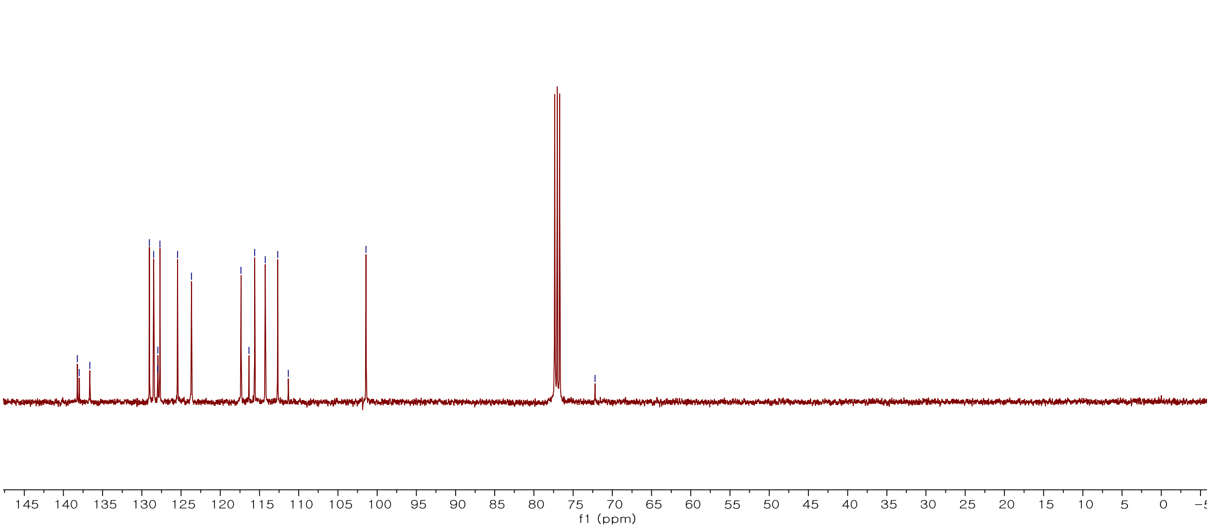
138.23
137.98
136.64
129.04
128.49
127.99
127.96
127.69
125.45
123.67
117.36
116.35
115.60
114.26
112.68
111.32
101.42
72.21
145 140 135 130 125 120 115 110 105 100 95 90 85 80 75 70 65 60 55 50 45 40 35 30 25 20 15 10 5 0
f1 (ppm)

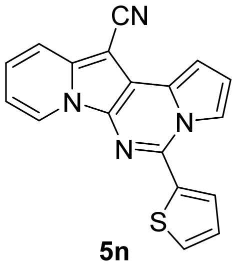
CN
N
N
N
S

5n

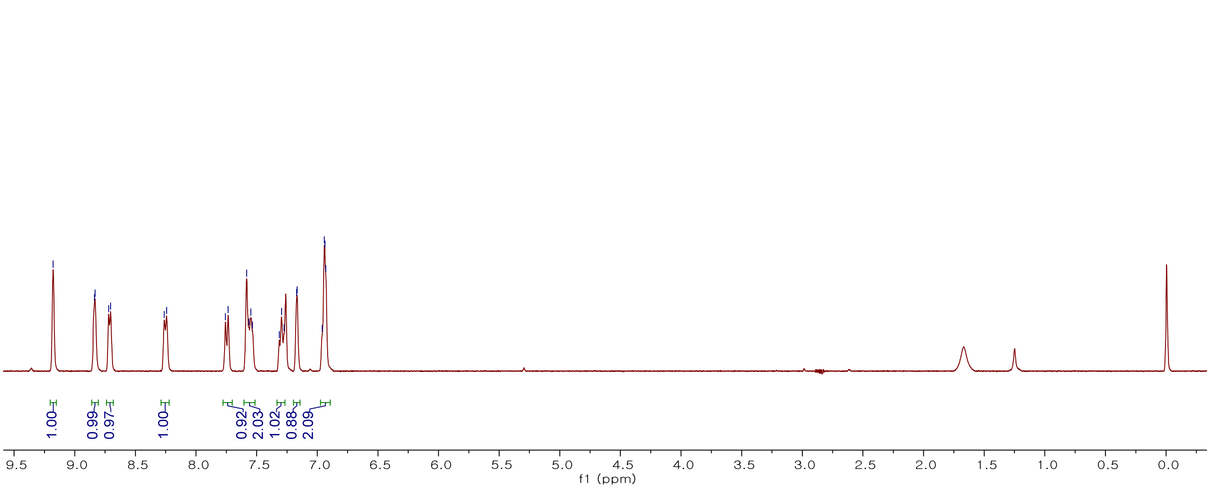
9.178
8.838
8.832
8.720
8.704
8.262
8.242
7.758
7.736
7.583
7.563
7.548
7.534
7.313
7.294
7.274
7.170
7.167
6.959
6.942
6.936
6.930
1.00
0.99
0.97
1.00
0.92
2.03
1.02
0.88
2.09
9.5 9.0 8.5 8.0 7.5 7.0 6.5 6.0 5.5 5.0 4.5 4.0 3.5 3.0 2.5 2.0 1.5 1.0 0.5 0.0
f1 (ppm)

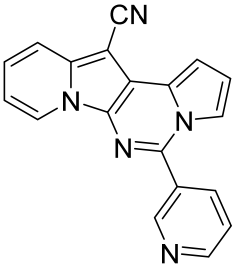
CN
N
N
N
N

5o

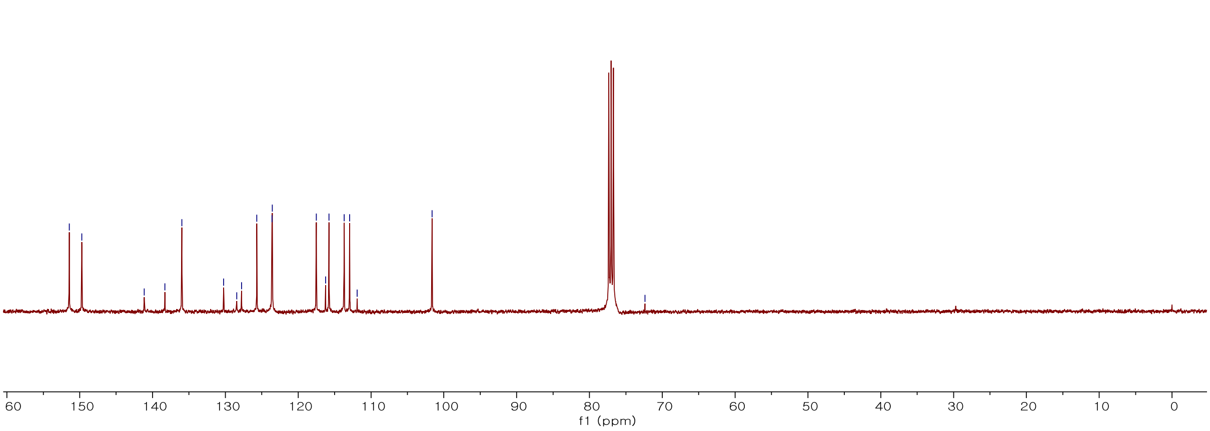
151.44
149.71
141.14
138.31
135.98
130.23
128.44
127.78
125.68
123.60
123.55
117.51
116.24
115.77
113.69
112.93
111.90
101.61
72.38
160 150 140 130 120 110 100 90 80 70 60 50 40 30 20 10 0
f1 (ppm)

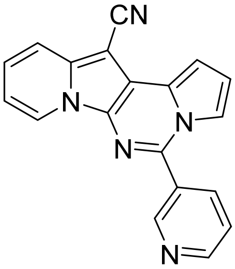
CN
N
N
N
N

5o

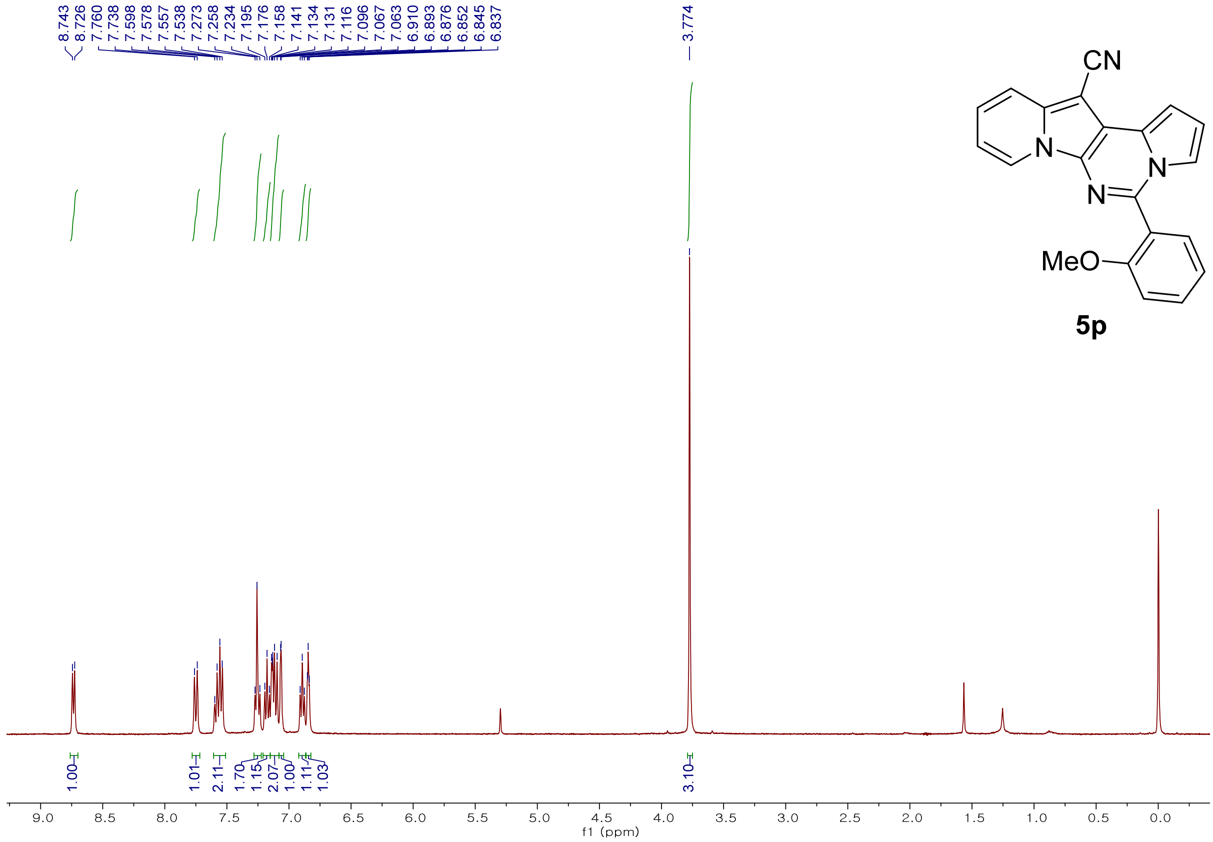
8.743
8.726
7.760
7.738
7.598
7.578
7.557
7.538
7.273
7.258
7.234
7.195
7.176
7.158
7.141
7.134
7.131
7.116
7.096
7.067
7.063
6.910
6.893
6.876
6.852
6.845
6.837
3.774
CN
N
N
N
MeO
5p
1.00
1.01
2.11
1.70
1.15
2.07
1.00
1.11
1.03
3.10
f1 (ppm)

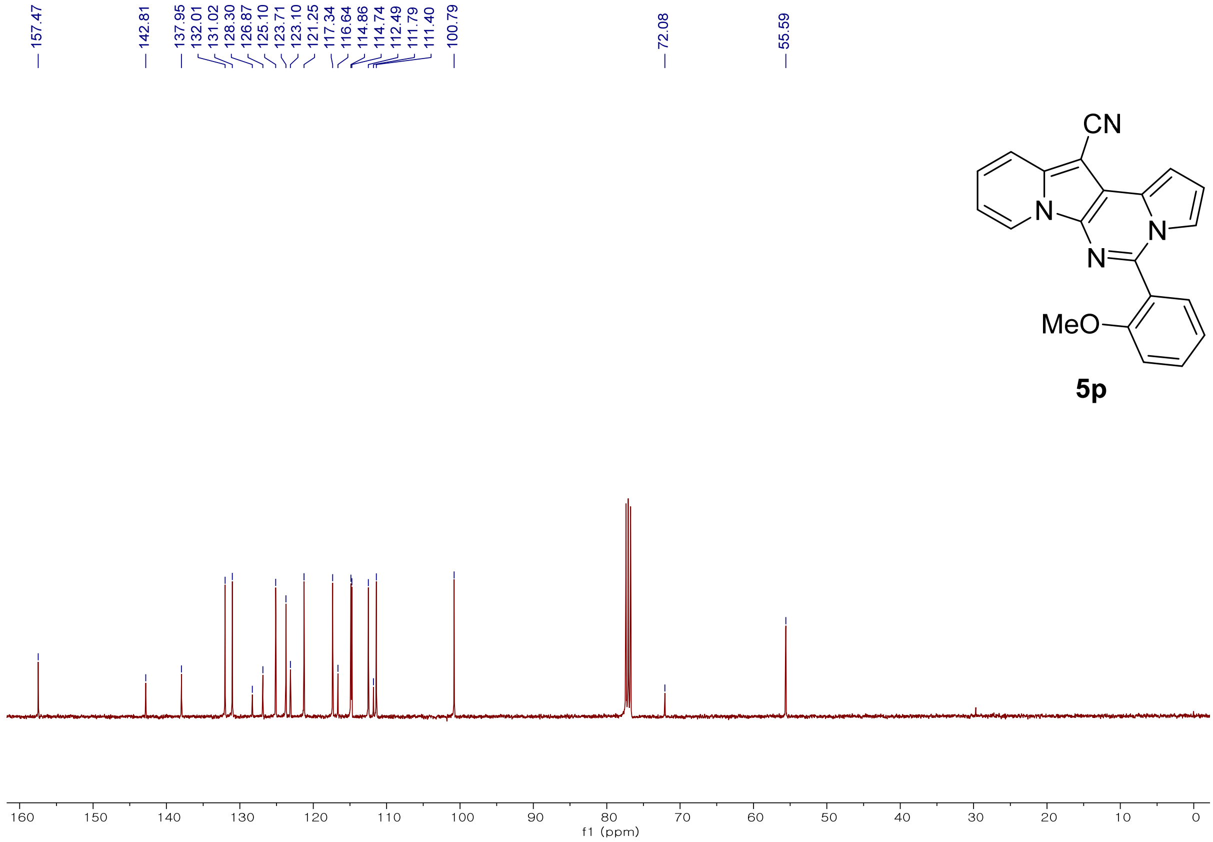
157.47
142.81
137.95
132.01
131.02
128.30
126.87
125.10
123.71
123.10
121.25
117.34
116.64
114.86
114.74
112.49
111.79
111.40
100.79
72.08
55.59
CN
N
N
N
MeO
5p
f1 (ppm)

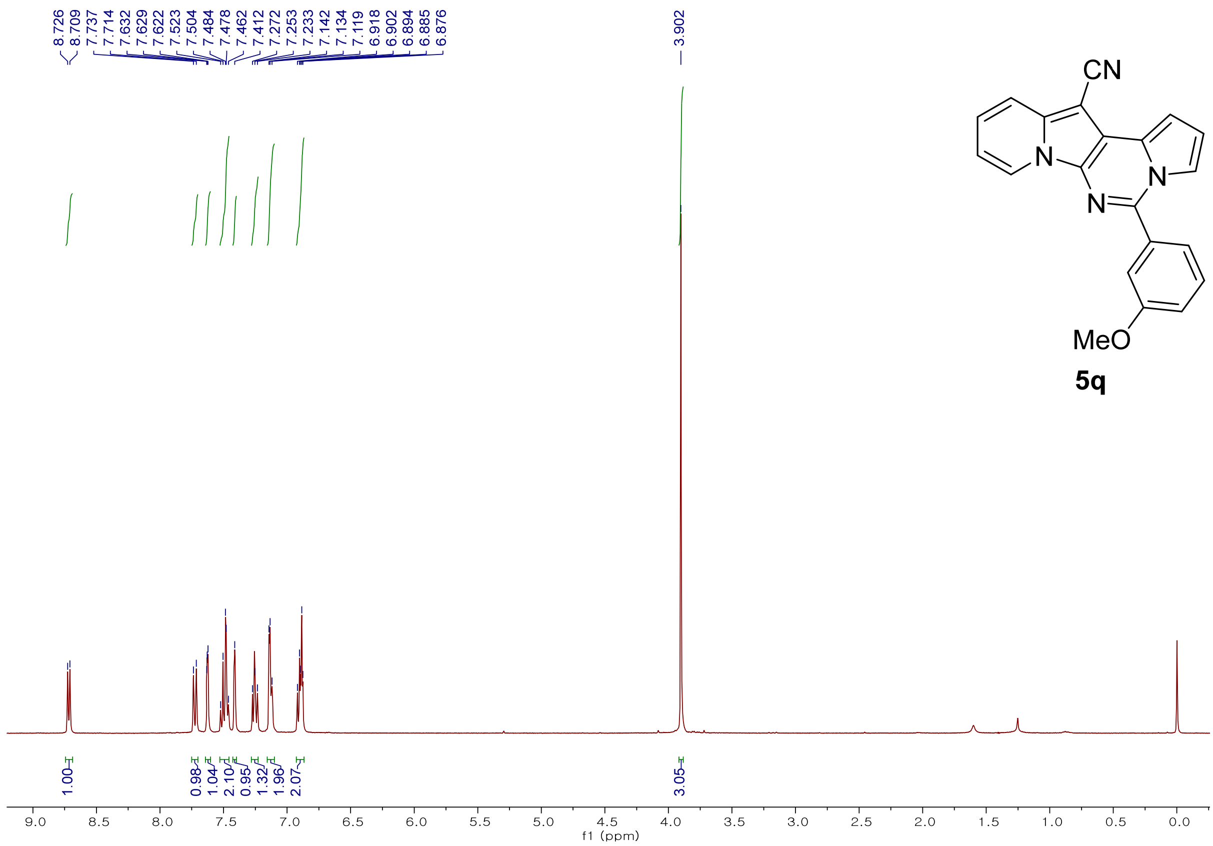

CN
N
N
N
MeO
5q
f1 (ppm)

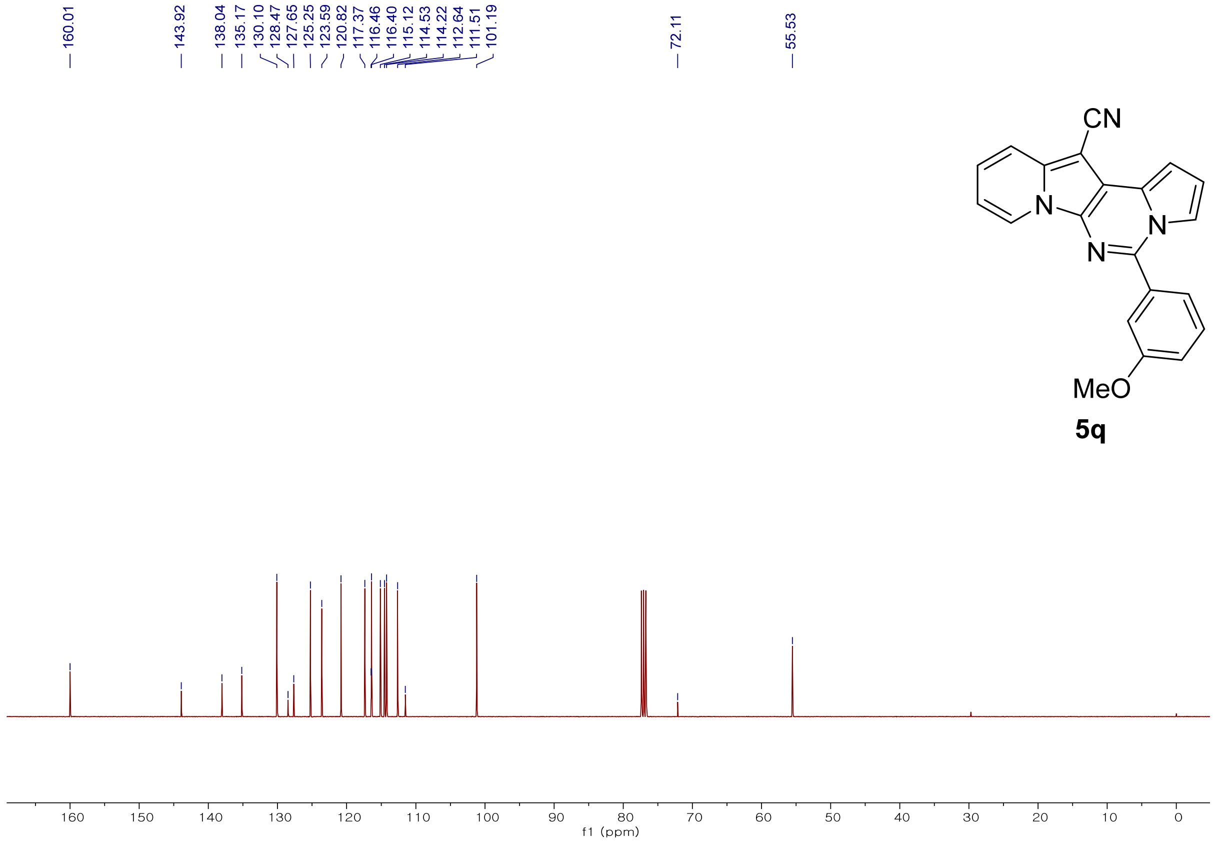

CN
N
N
N
MeO
5q
f1 (ppm)

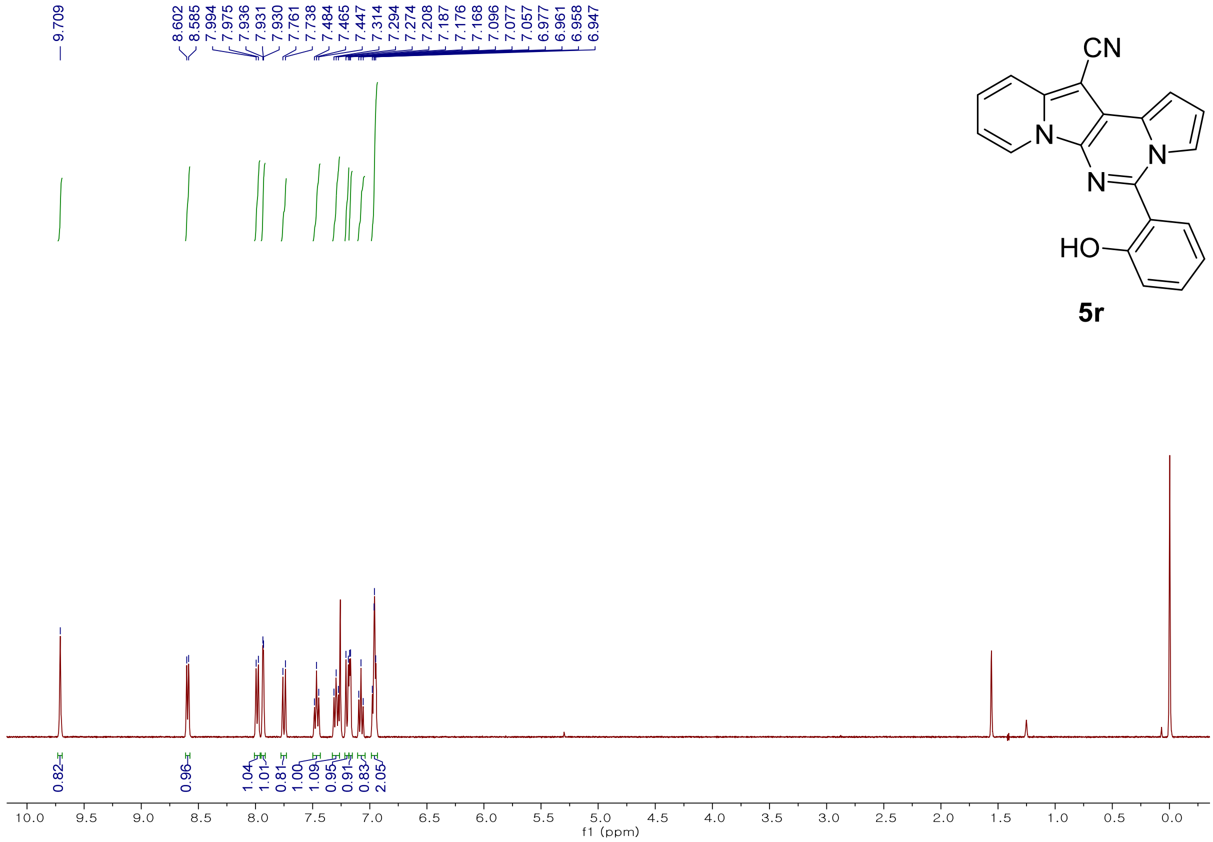
9.709
8.602 8.585 7.994 7.975 7.936 7.931 7.930 7.761 7.738 7.484 7.465 7.447 7.314 7.294 7.274 7.208 7.187 7.176 7.168 7.096 7.077 7.057 6.977 6.961 6.958 6.947
CN
N
N
N
HO
5r
0.82 0.96 1.04 1.01 0.81 1.00 1.09 0.95 0.91 0.83 2.05
f1 (ppm)

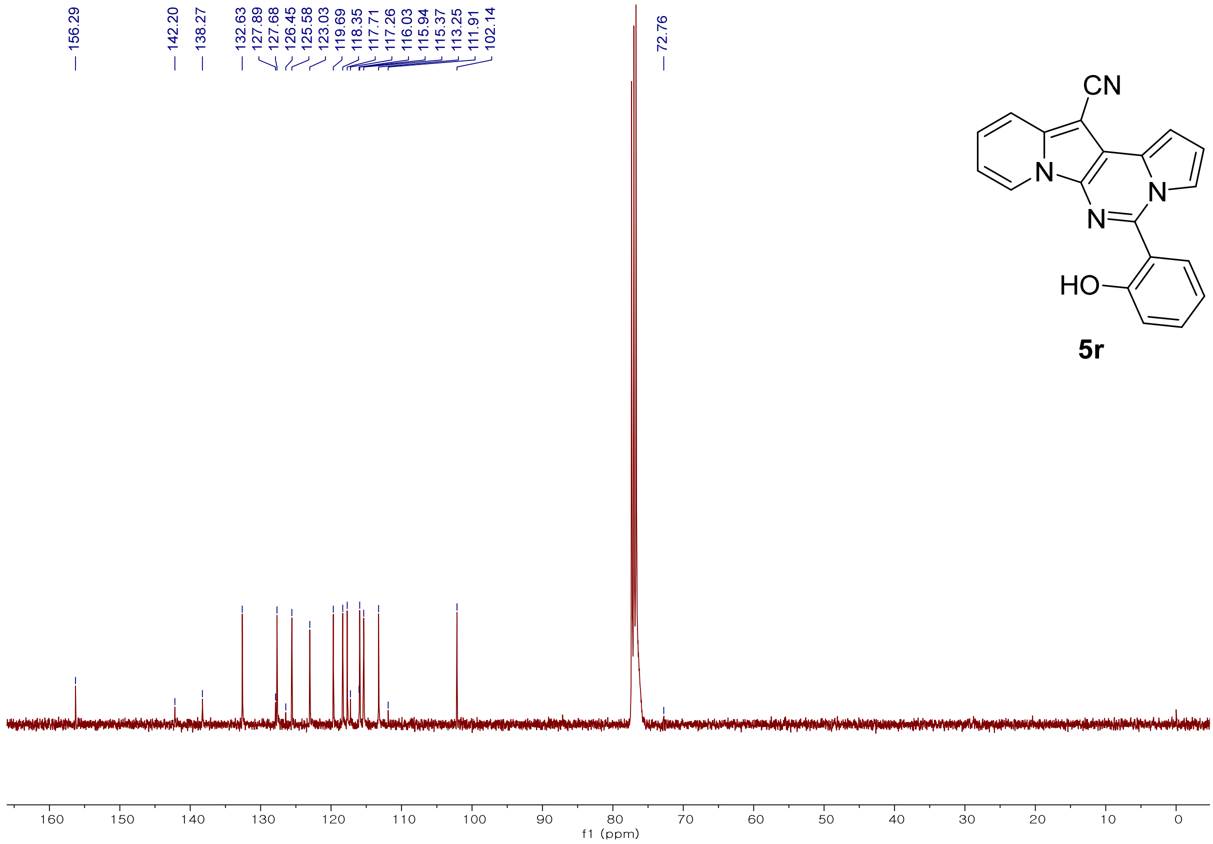
156.29
142.20
138.27
132.63 127.89 127.68 126.45 125.58 123.03 119.69 118.35 117.71 117.26 116.03 115.94 115.37 113.25 111.91 102.14
72.76
CN
N
N
N
HO
5r
f1 (ppm)

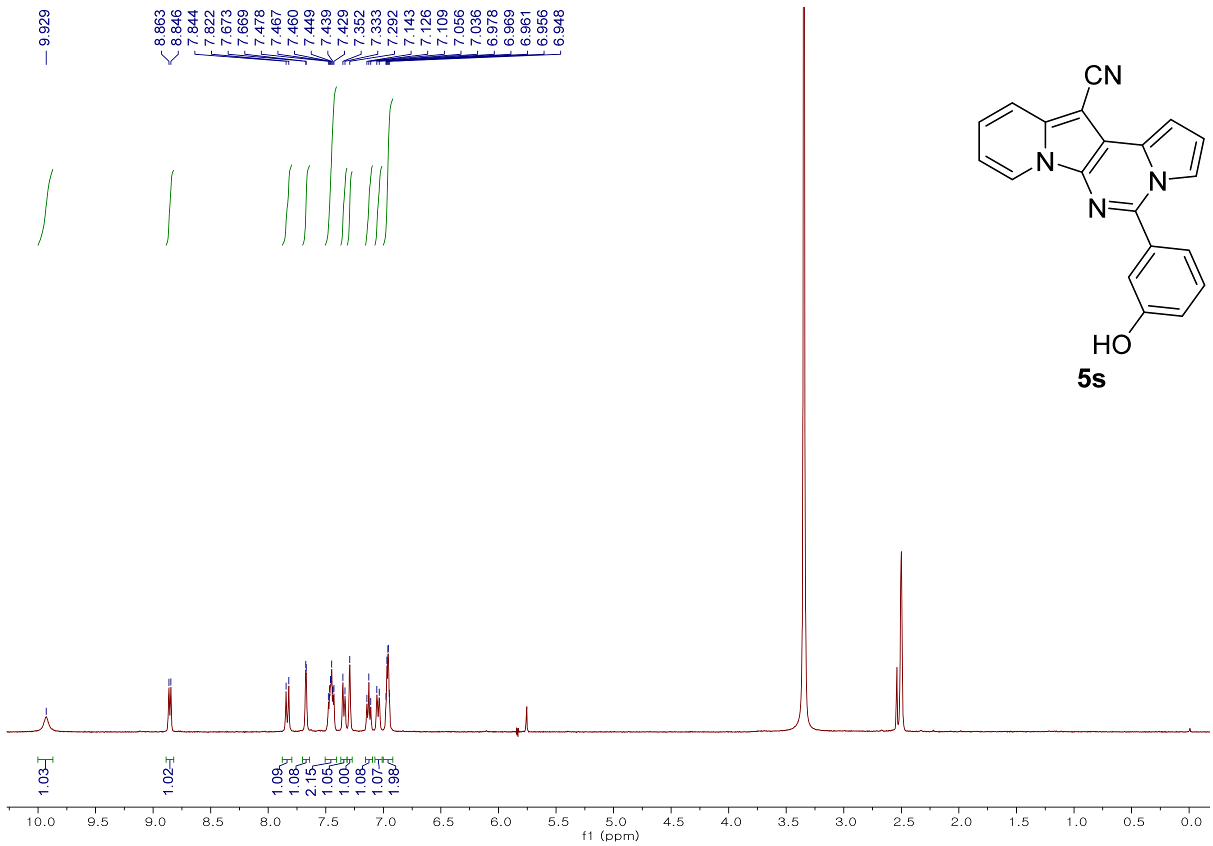

9.929
8.863
8.846
7.844
7.822
7.673
7.669
7.478
7.467
7.460
7.449
7.439
7.429
7.352
7.333
7.292
7.143
7.126
7.109
7.056
7.036
6.978
6.969
6.961
6.956
6.948
CN
N
N
N
HO
5s
1.03
1.02
1.09
1.08
2.15
1.05
1.00
1.08
1.07
1.98
f1 (ppm)

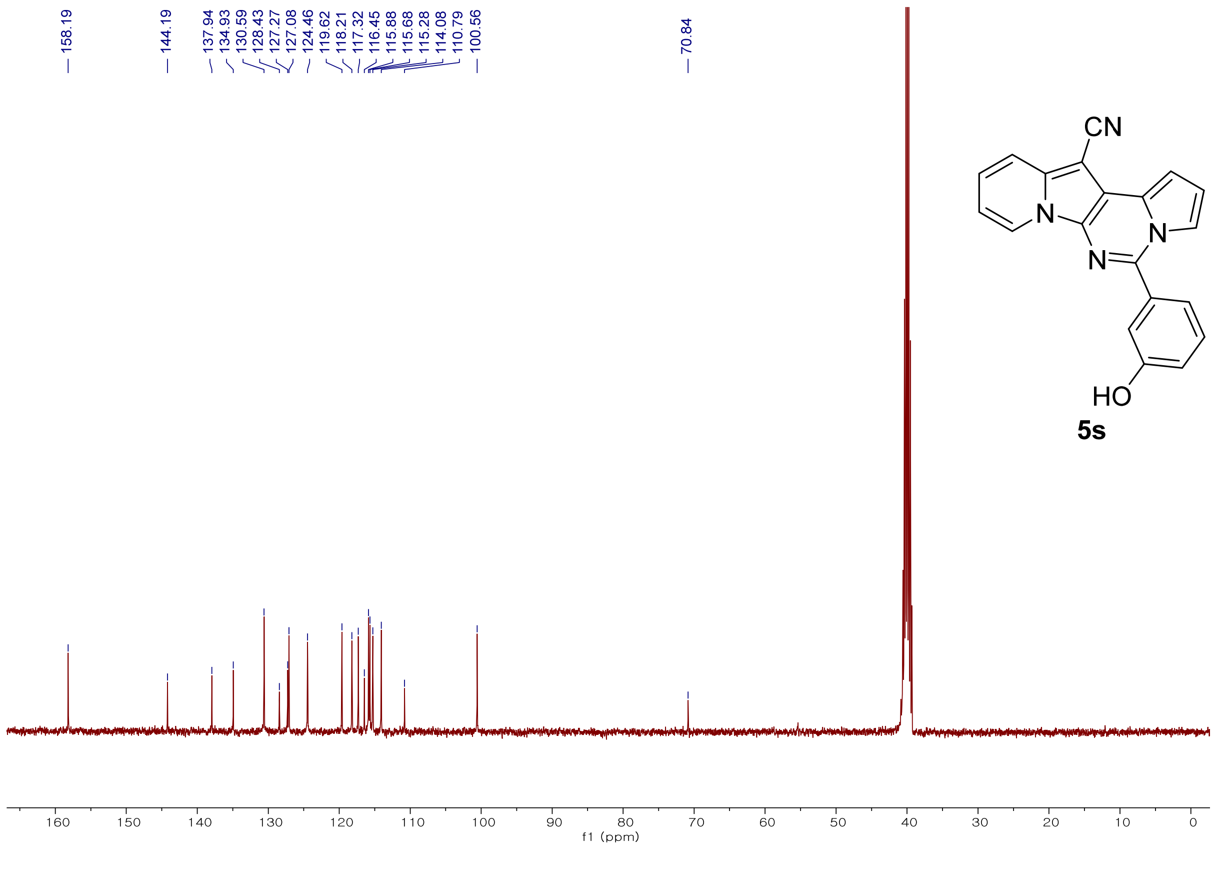

158.19
144.19
137.94
134.93
130.59
128.43
127.27
127.08
124.46
119.62
118.21
117.32
116.45
115.88
115.68
115.28
114.08
110.79
100.56
70.84
CN
N
N
N
HO
5s
f1 (ppm)

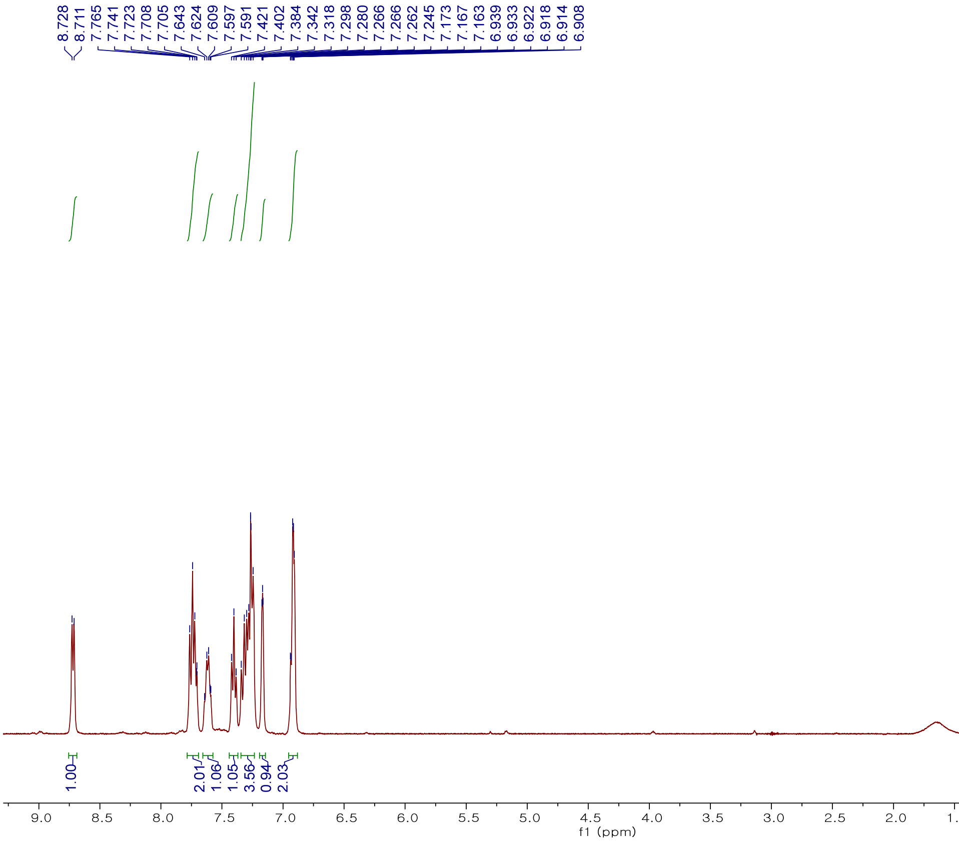
8.728
8.711
7.765
7.741
7.723
7.708
7.705
7.643
7.624
7.609
7.597
7.591
7.421
7.402
7.384
7.342
7.318
7.298
7.280
7.266
7.266
7.262
7.245
7.173
7.163
6.939
6.933
6.922
6.918
6.914
6.908
1.00
2.01
1.06
1.05
3.56
0.94
2.03
9.0 8.5 8.0 7.5 7.0 6.5 6.0 5.5 5.0 4.5 4.0 3.5 3.0 2.5 2.0 1.5 1.0 0.5 0.0 -0.
f1 (ppm)

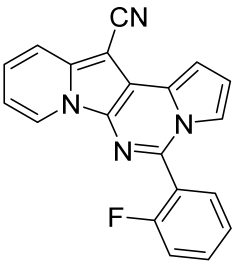
CN
N
N
N
F

5t

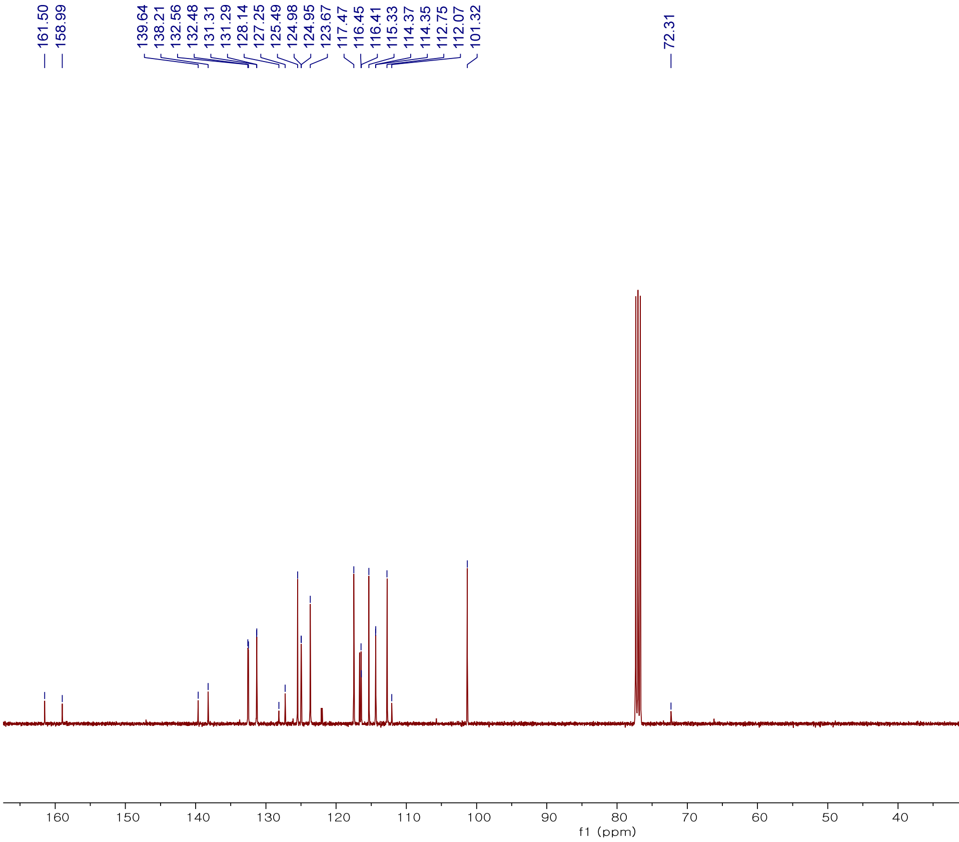
161.50
158.99
139.64
138.21
132.56
132.48
131.31
131.29
128.14
127.25
125.49
124.98
124.95
123.67
117.47
116.45
116.41
115.33
114.37
114.35
112.75
112.07
101.32
72.31
160 150 140 130 120 110 100 90 80 70 60 50 40 30 20 10 0
f1 (ppm)

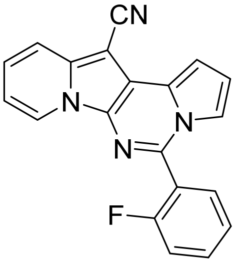
CN
N
N
N
F

5t

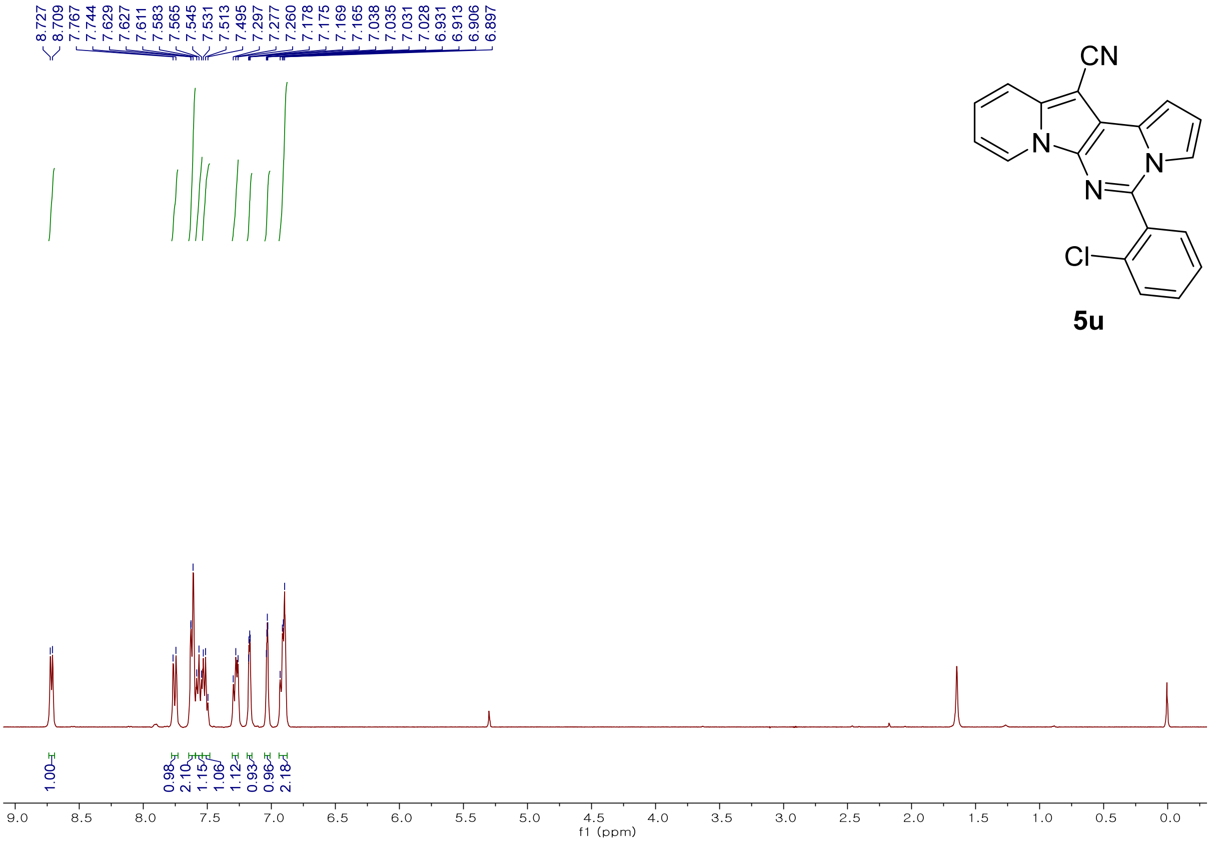
8.727
8.709
7.767
7.744
7.629
7.627
7.611
7.583
7.565
7.545
7.531
7.513
7.495
7.297
7.277
7.260
7.178
7.175
7.169
7.165
7.038
7.035
7.031
7.028
6.931
6.913
6.906
6.897
CN
N
N
N
Cl
5u
1.00
0.98
2.10
1.15
1.06
1.12
0.93
0.96
2.18
f1 (ppm)

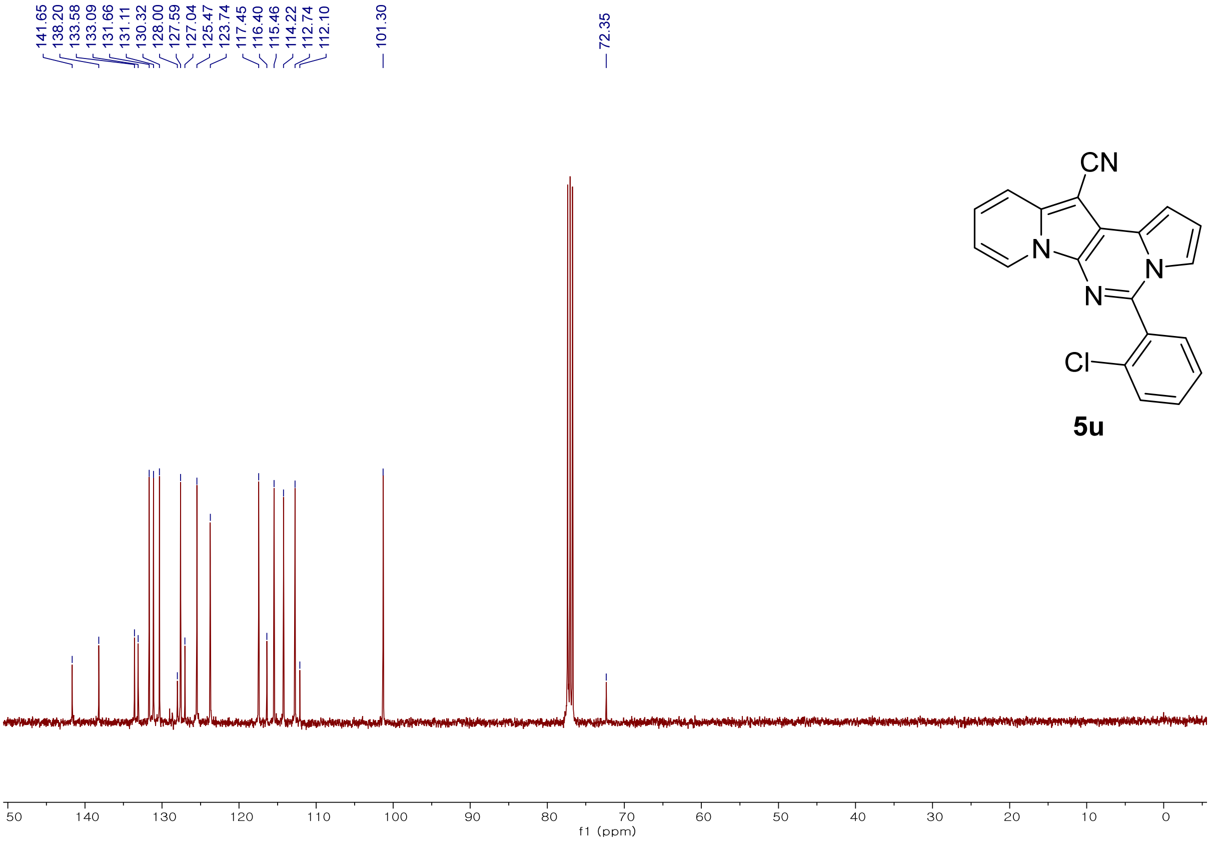
141.65
138.20
133.58
133.09
131.66
131.11
130.32
128.00
127.59
127.04
125.47
123.74
117.45
116.40
115.46
114.22
112.74
112.10
101.30
72.35
CN
N
N
N
Cl
5u
f1 (ppm)

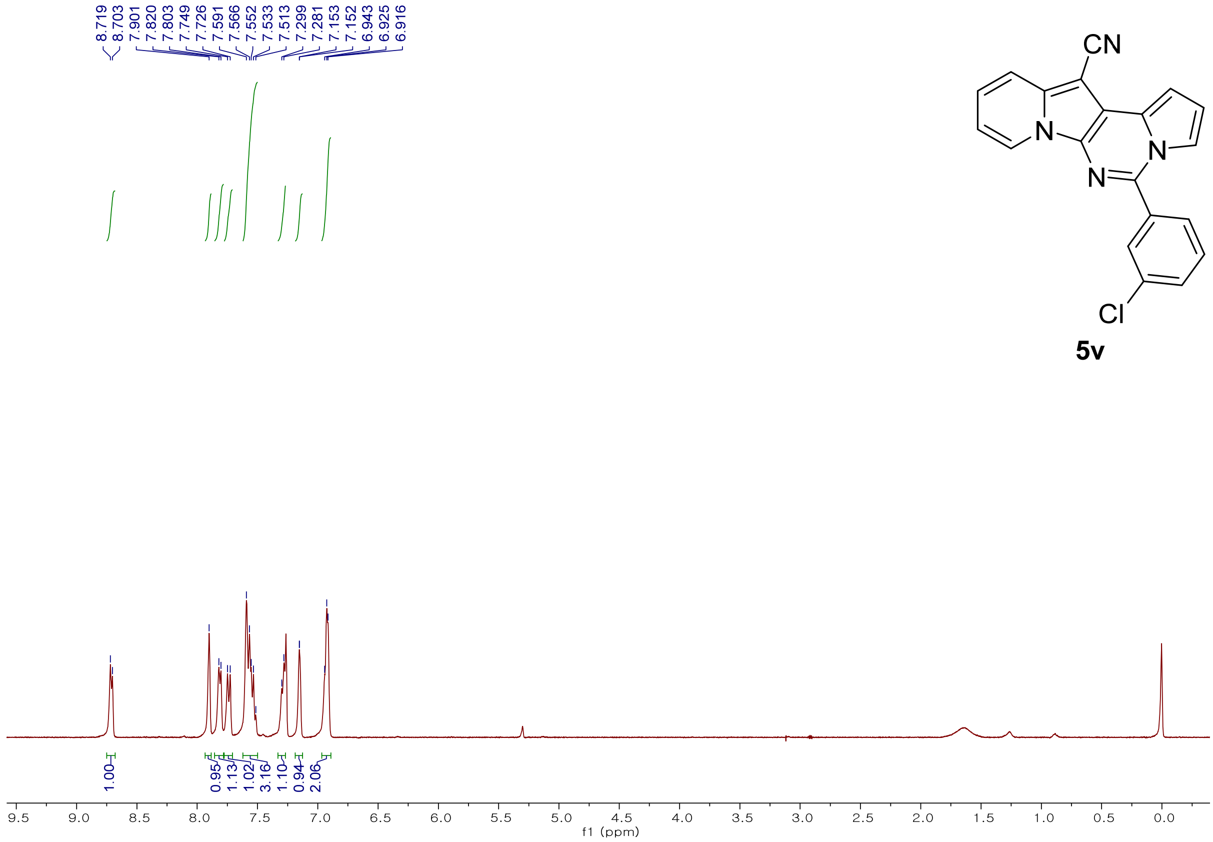

8.719
8.703
7.901
7.820
7.803
7.749
7.726
7.591
7.566
7.552
7.533
7.513
7.299
7.281
7.153
7.152
6.943
6.925
6.916
1.00
0.95
1.13
1.02
3.16
1.10
0.94
2.06
9.5 9.0 8.5 8.0 7.5 7.0 6.5 6.0 5.5 5.0 4.5 4.0 3.5 3.0 2.5 2.0 1.5 1.0 0.5 0.0
f1 (ppm)
CN
N
N
N
Cl

5v

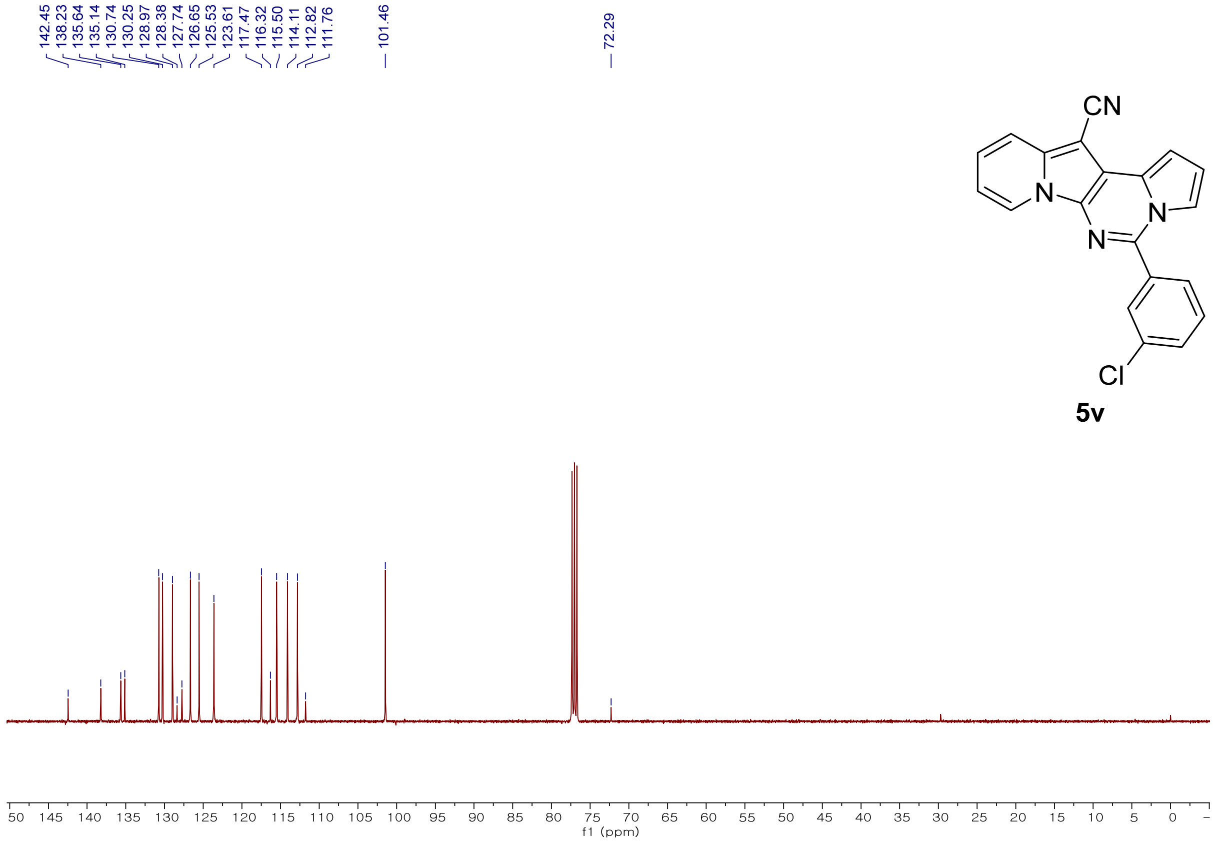

142.45
138.23
135.64
135.14
130.74
130.25
128.97
128.38
127.74
126.65
125.53
123.61
117.47
116.32
115.50
114.11
112.82
111.76
101.46
72.29
50 145 140 135 130 125 120 115 110 105 100 95 90 85 80 75 70 65 60 55 50 45 40 35 30 25 20 15 10 5 0
f1 (ppm)
CN
N
N
N
Cl

5v

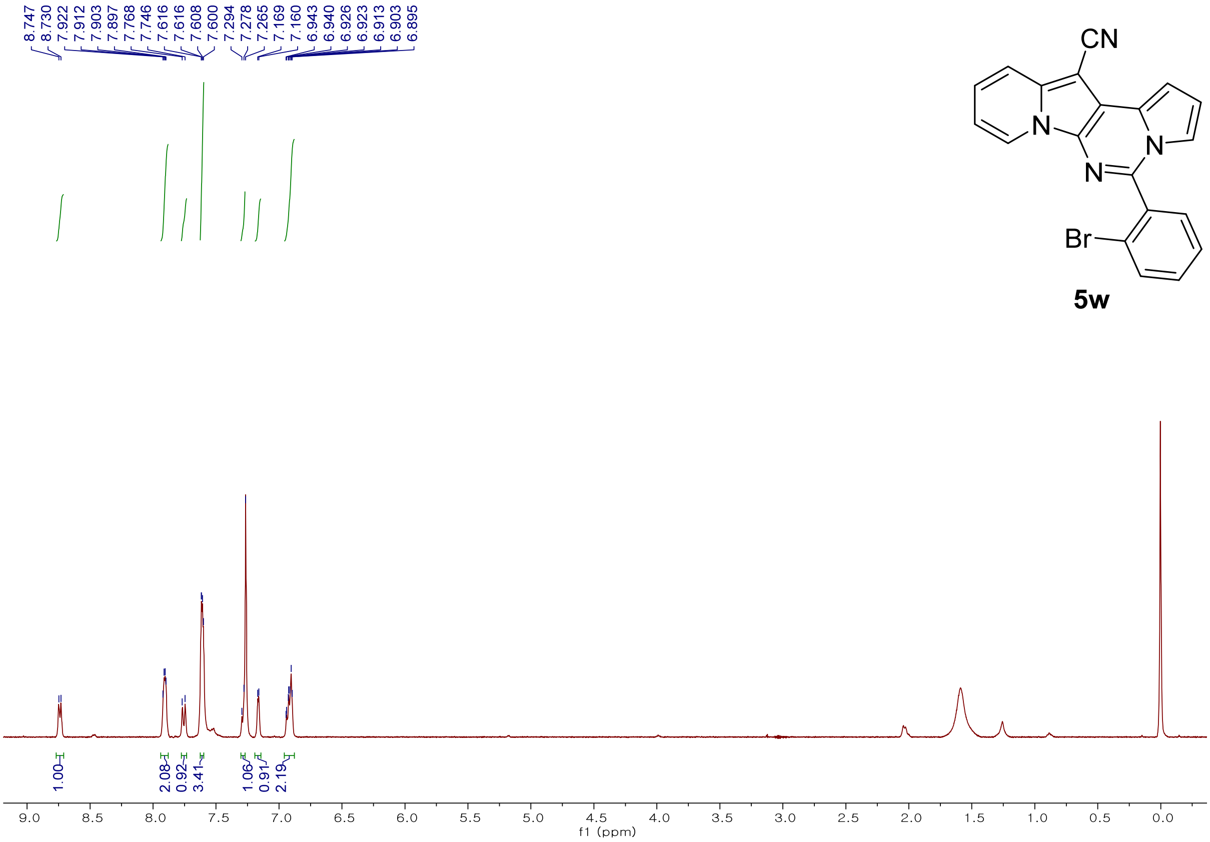

CN
N
N
N
Br
5w
8.747
8.730
7.922
7.912
7.903
7.897
7.768
7.746
7.616
7.616
7.608
7.600
7.294
7.278
7.265
7.169
7.160
6.943
6.940
6.926
6.923
6.913
6.903
6.895
1.00
2.08
0.92
3.41
1.06
0.91
2.19
9.0 8.5 8.0 7.5 7.0 6.5 6.0 5.5 5.0 4.5 4.0 3.5 3.0 2.5 2.0 1.5 1.0 0.5 0.0
f1 (ppm)

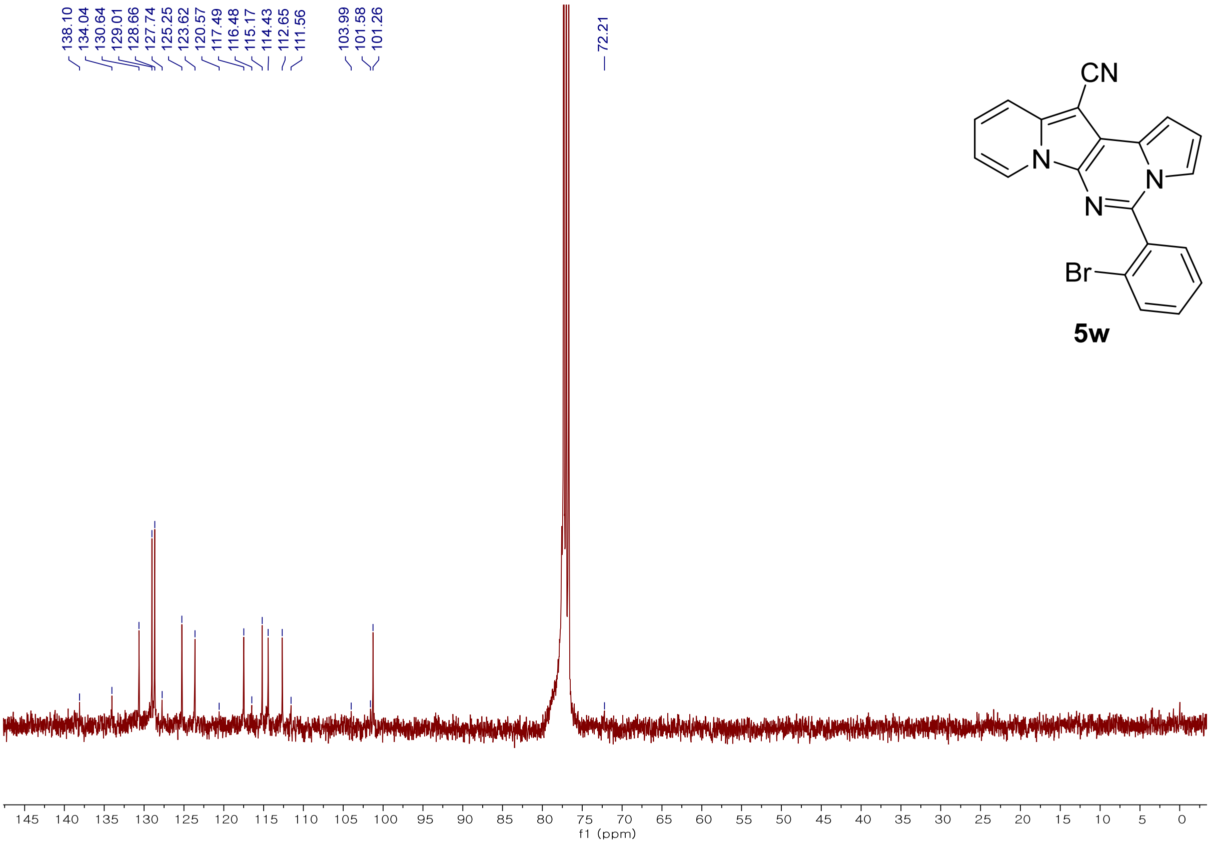

CN
N
N
N
Br
5w
138.10
134.04
130.64
129.01
128.66
127.74
125.25
123.62
120.57
117.49
116.48
115.17
114.43
112.65
111.56
103.99
101.58
101.26
72.21
145 140 135 130 125 120 115 110 105 100 95 90 85 80 75 70 65 60 55 50 45 40 35 30 25 20 15 10 5 0
f1 (ppm)

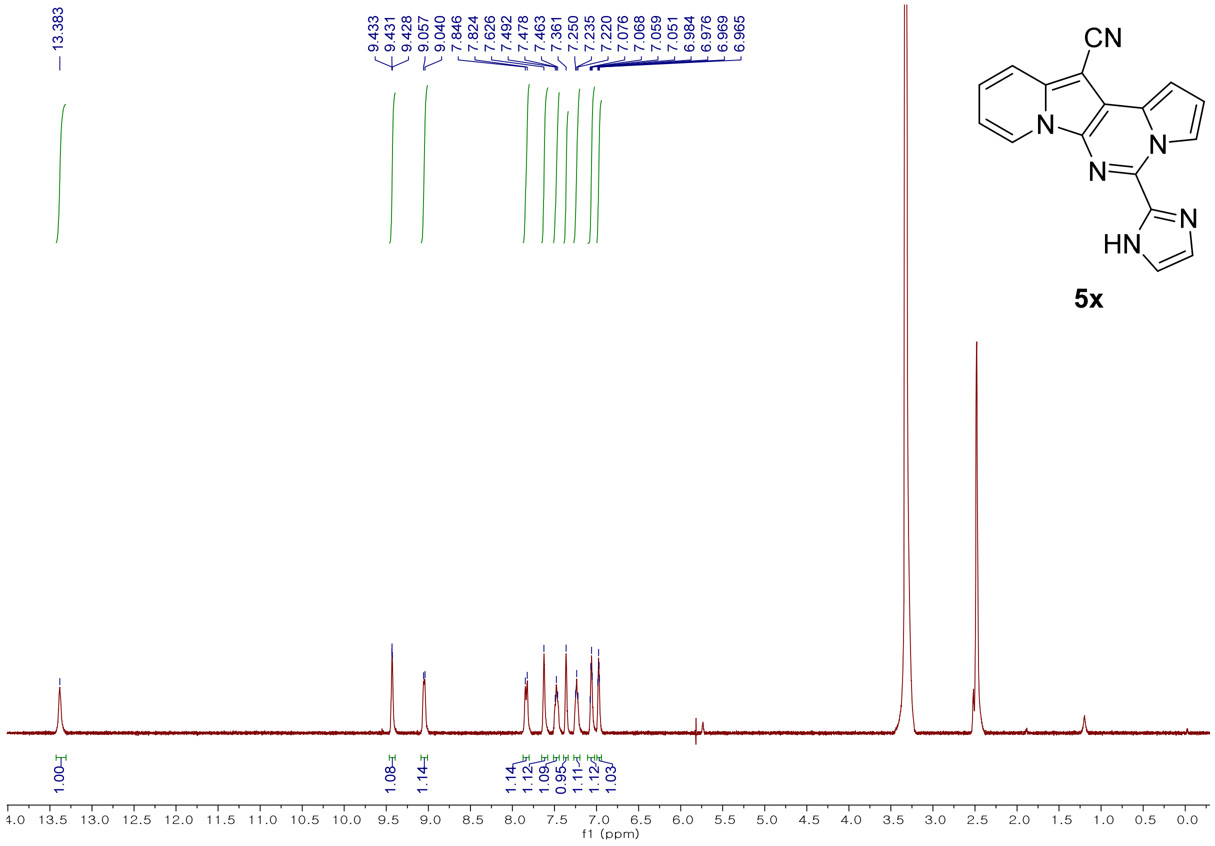
13.383
9.433
9.431
9.428
9.057
9.040
7.846
7.824
7.626
7.492
7.478
7.463
7.361
7.250
7.235
7.220
7.076
7.068
7.059
7.051
6.984
6.976
6.969
6.965
CN
N
N
N
HN
N
5x
1.00
1.08
1.14
1.14
1.12
1.09
0.95
1.11
1.12
1.03
f1 (ppm)

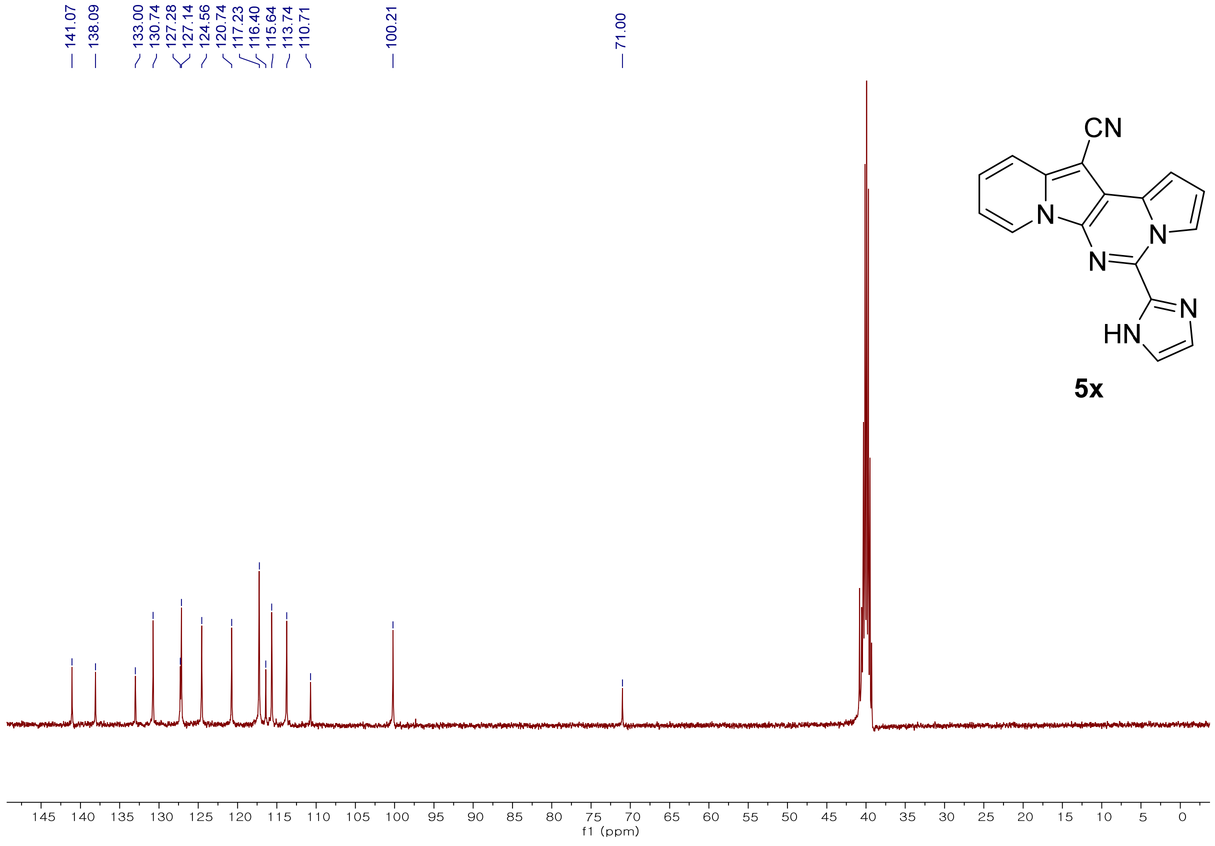
141.07
138.09
133.00
130.74
127.28
127.14
124.56
120.74
117.23
116.40
115.64
113.74
110.71
100.21
71.00
CN
N
N
N
HN
N
5x
f1 (ppm)

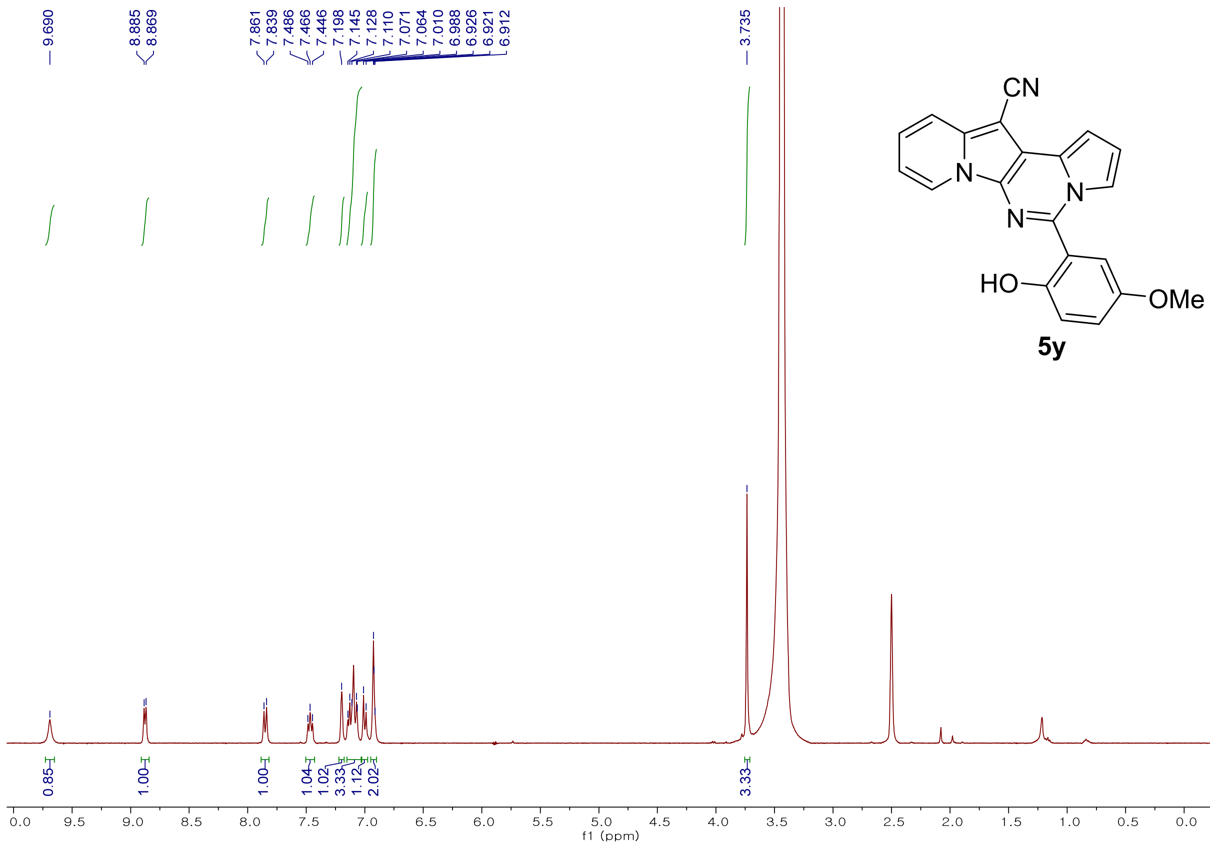

9.690
8.885
8.869
7.861
7.839
7.486
7.466
7.446
7.198
7.145
7.128
7.110
7.071
7.064
7.010
6.988
6.926
6.921
6.912
3.735
0.85
1.00
1.00
1.04
1.02
3.33
1.12
2.02
3.33
f1 (ppm)
CN
N
N
N
HO
OMe
5y

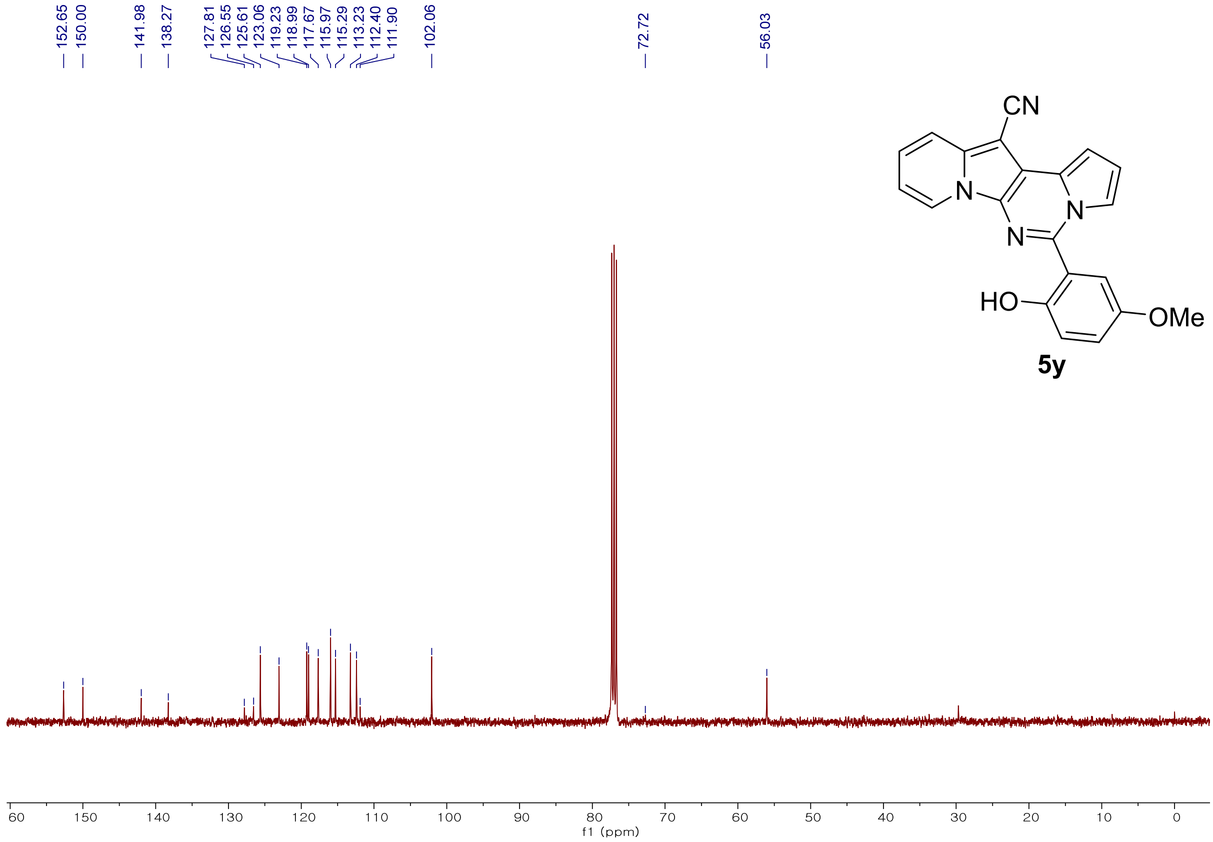

152.65
150.00
141.98
138.27
127.81
126.55
125.61
123.06
119.23
118.99
117.67
115.97
115.29
113.23
112.40
111.90
102.06
72.72
56.03
f1 (ppm)
CN
N
N
N
HO
OMe
5y

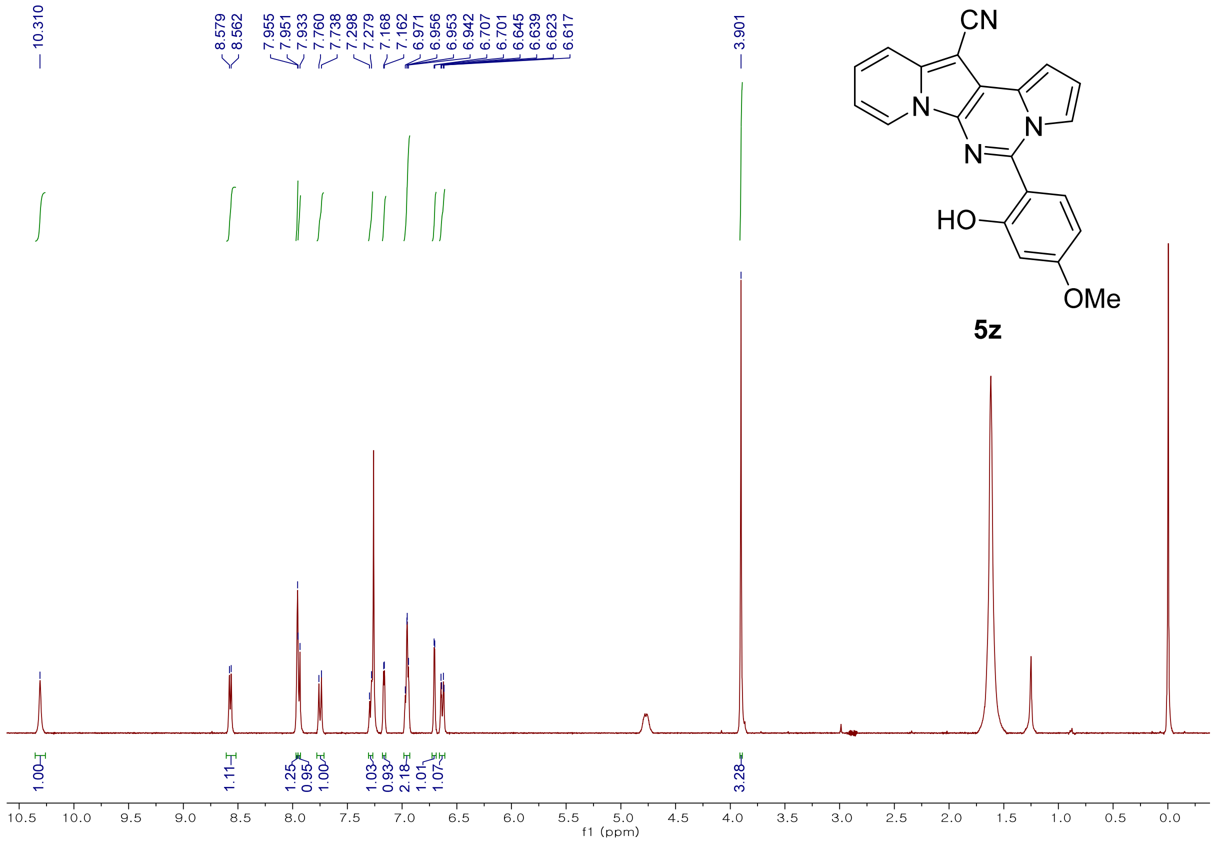

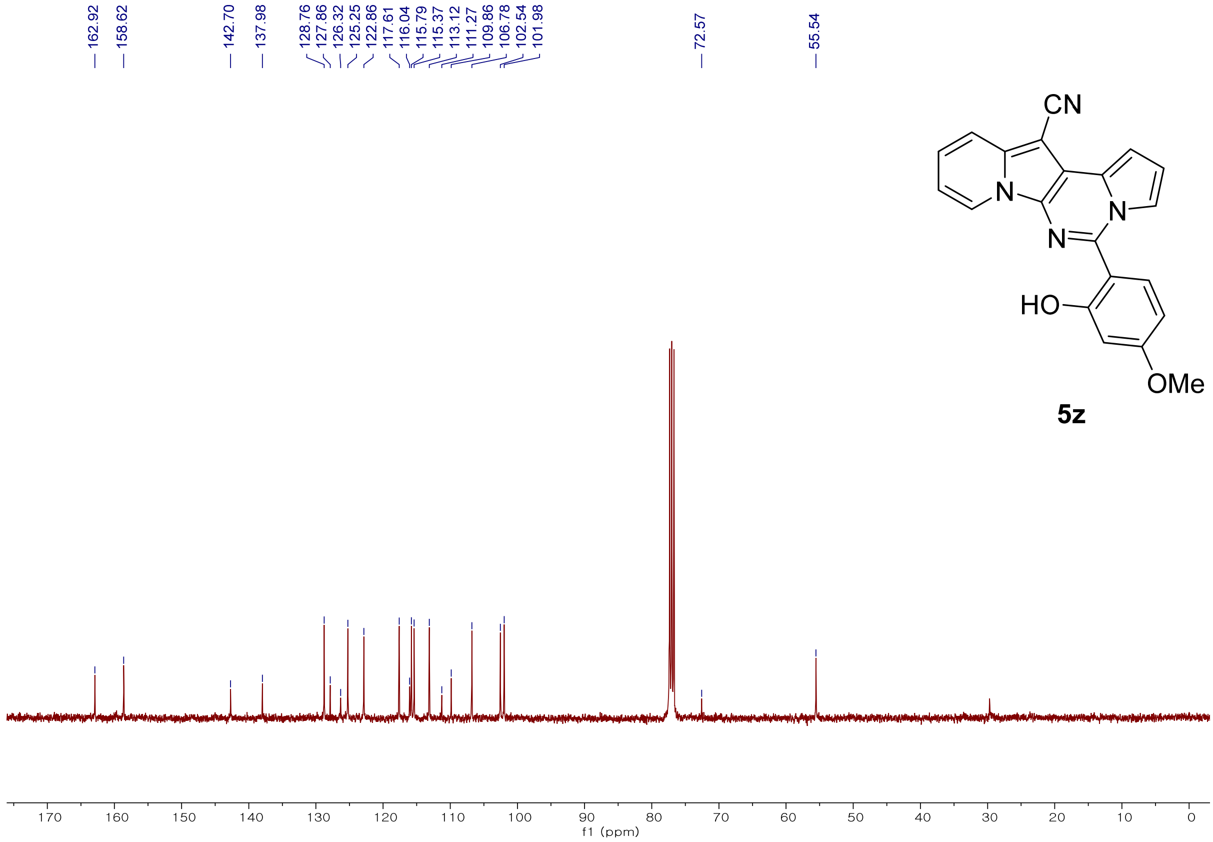

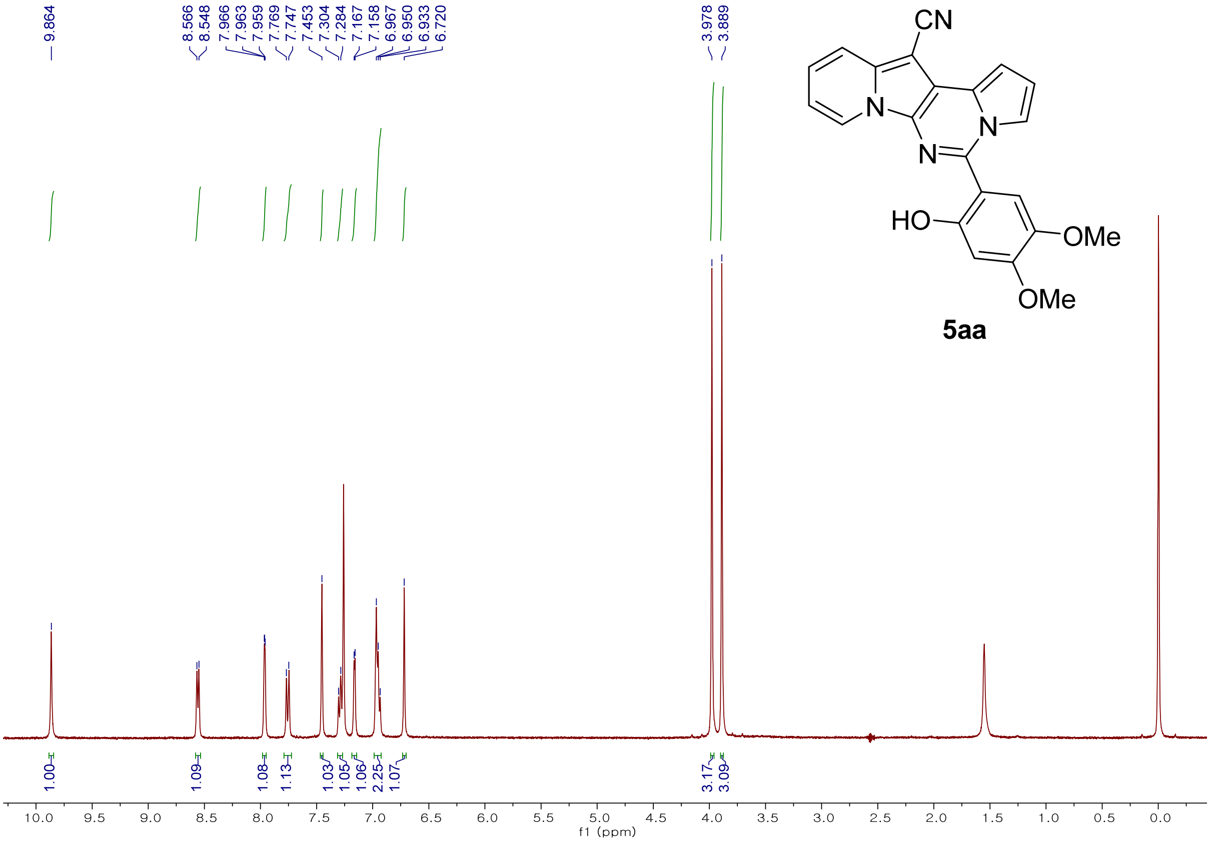
9.864
8.566
8.548
7.966
7.963
7.959
7.769
7.747
7.453
7.304
7.284
7.167
7.158
6.967
6.950
6.933
6.720
3.978
3.889
CN
N
N
N
HO
OMe
OMe
5aa
1.00
1.09
1.08
1.13
1.03
1.05
1.06
2.25
1.07
3.17
3.09
f1 (ppm)

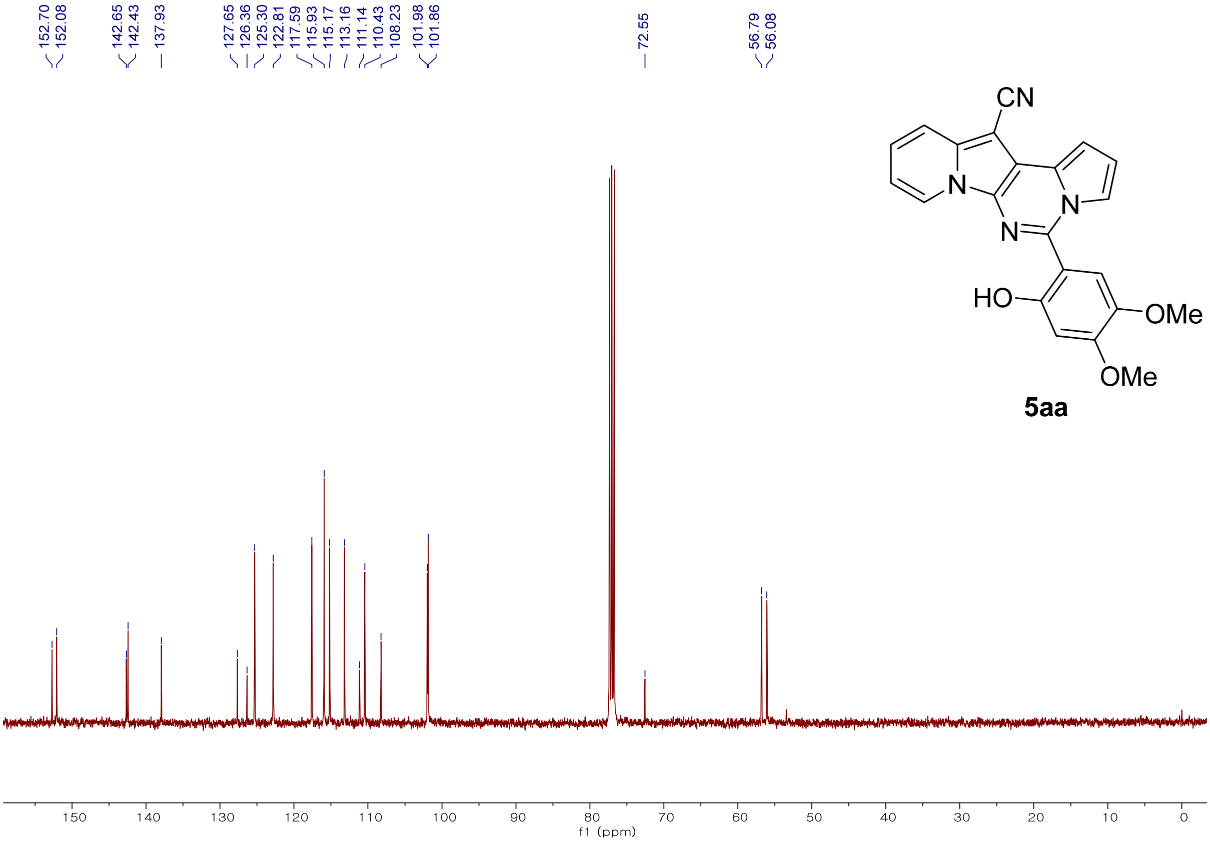
152.70
152.08
142.65
142.43
137.93
127.65
126.36
125.30
122.81
117.59
115.93
115.17
113.16
111.14
110.43
108.23
101.98
101.86
72.55
56.79
56.08
CN
N
N
N
HO
OMe
OMe
5aa
f1 (ppm)

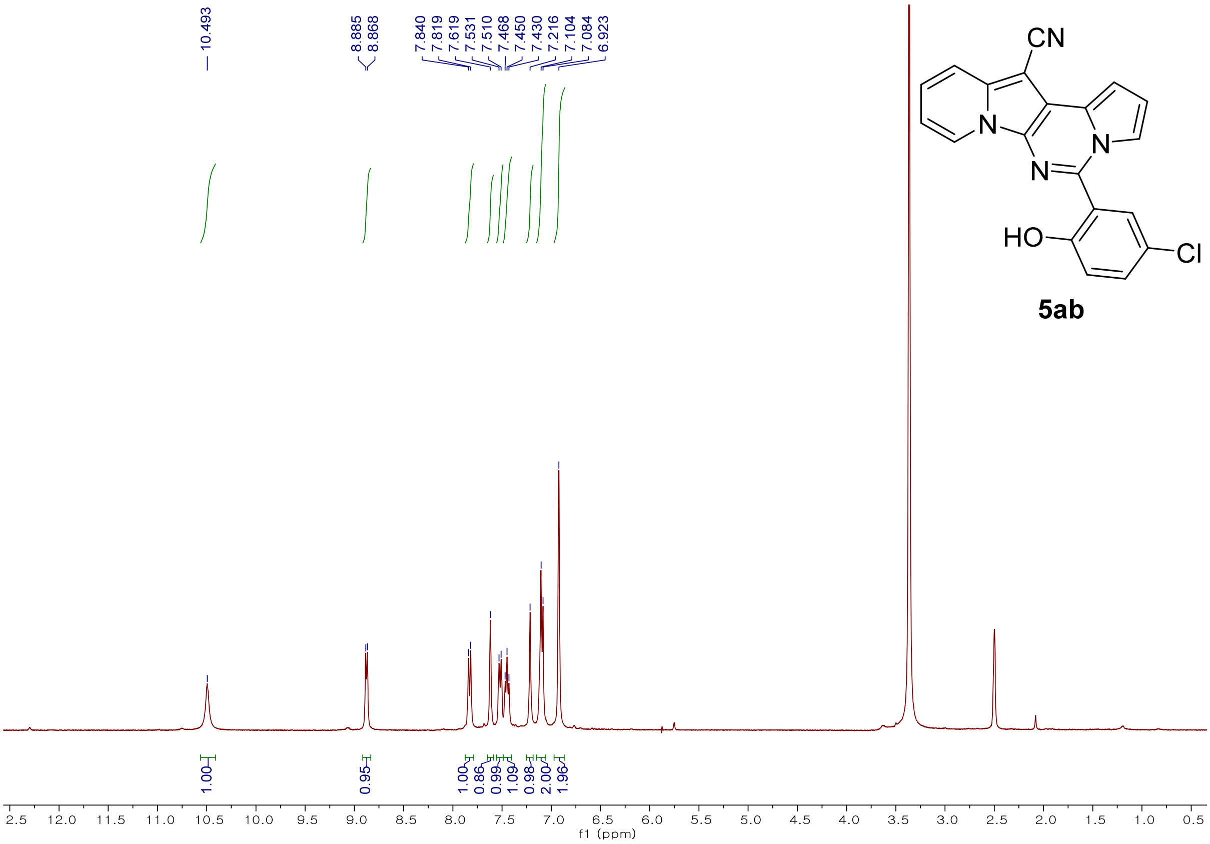
10.493
8.885
8.868
7.840
7.819
7.619
7.531
7.510
7.468
7.450
7.430
7.216
7.104
7.084
6.923
CN
N
N
N
HO
Cl
5ab
1.00
0.95
1.00
0.86
0.99
1.09
0.98
2.00
1.96
f1 (ppm)

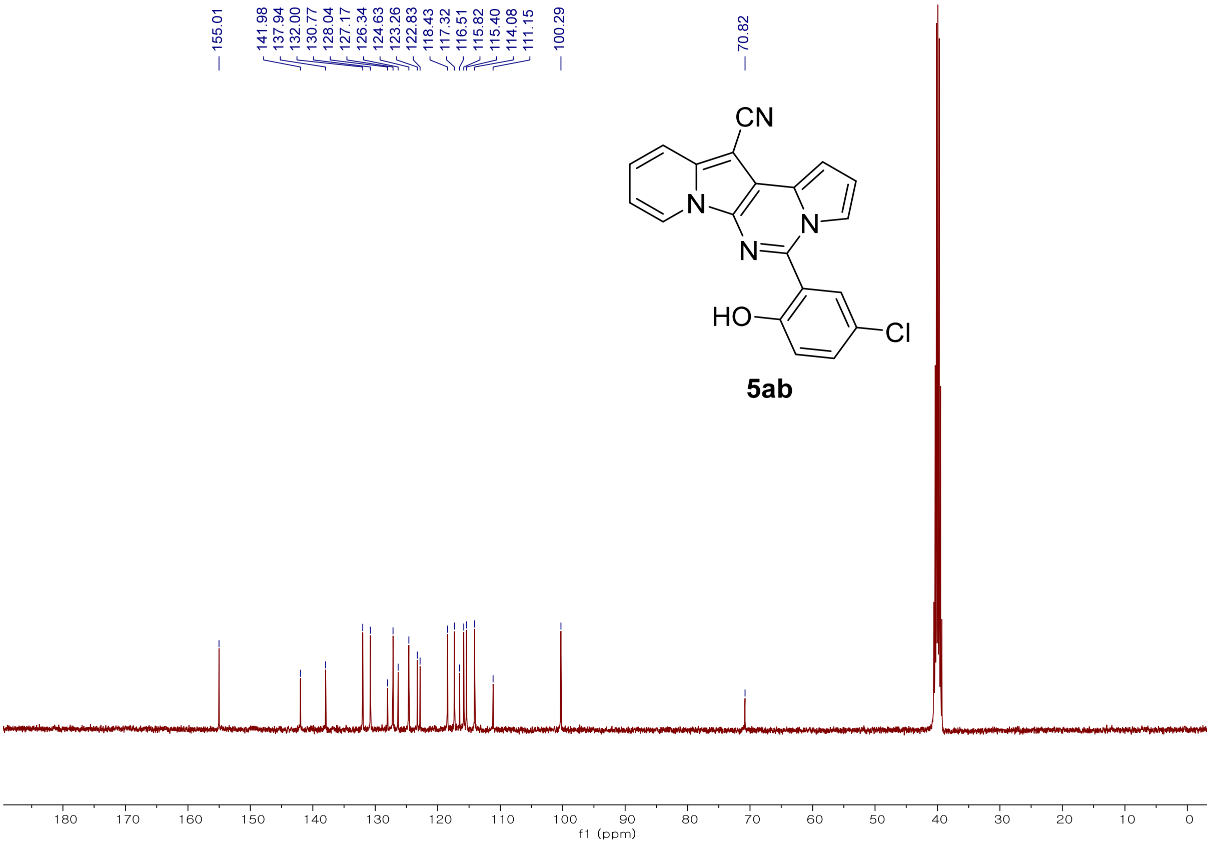
155.01
141.98
137.94
132.00
130.77
128.04
127.17
126.34
124.63
123.26
122.83
118.43
117.32
116.51
115.82
115.40
114.08
111.15
100.29
70.82
CN
N
N
N
HO
Cl
5ab
f1 (ppm)

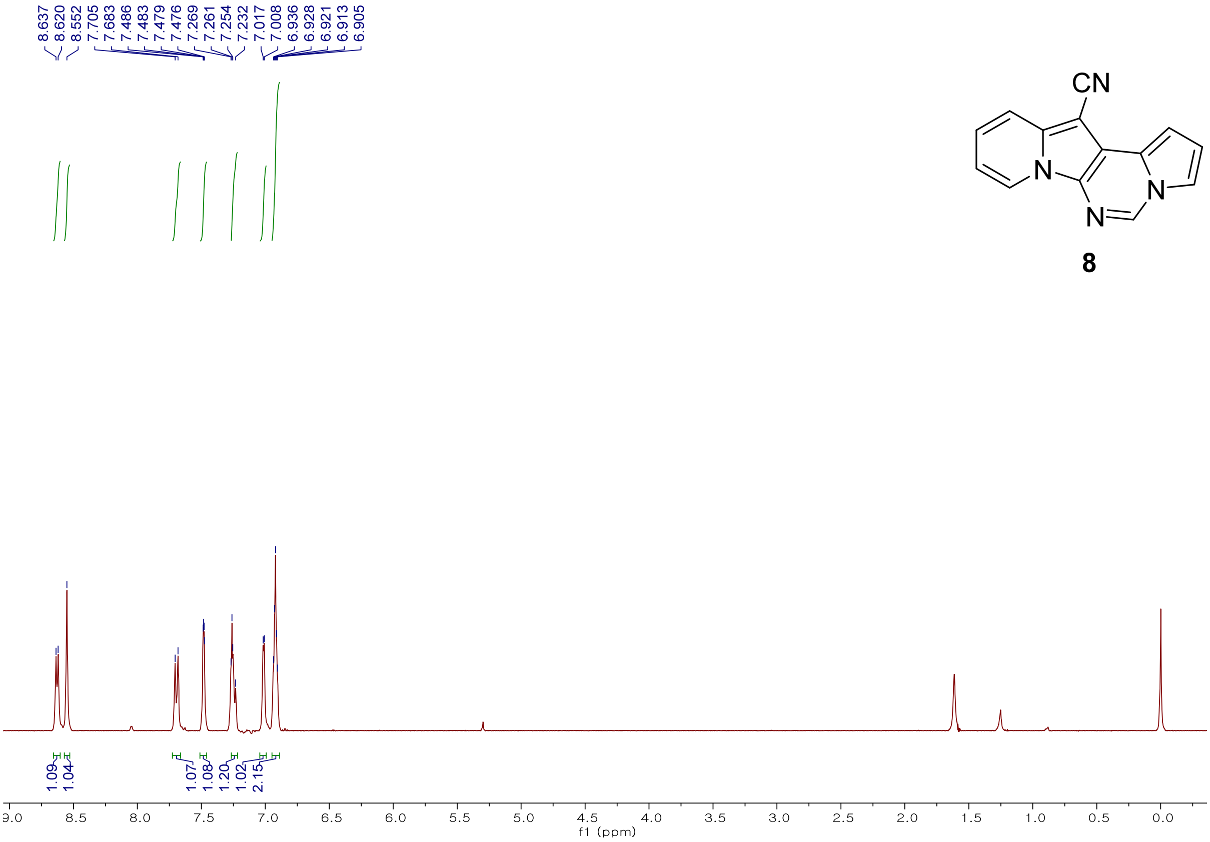
8.637
8.620
8.552
7.705
7.683
7.486
7.483
7.479
7.476
7.269
7.261
7.254
7.232
7.017
7.008
6.936
6.928
6.921
6.913
6.905
CN
N
N
N
1.09
1.04
1.07
1.08
1.20
1.02
2.15
f1 (ppm)

8

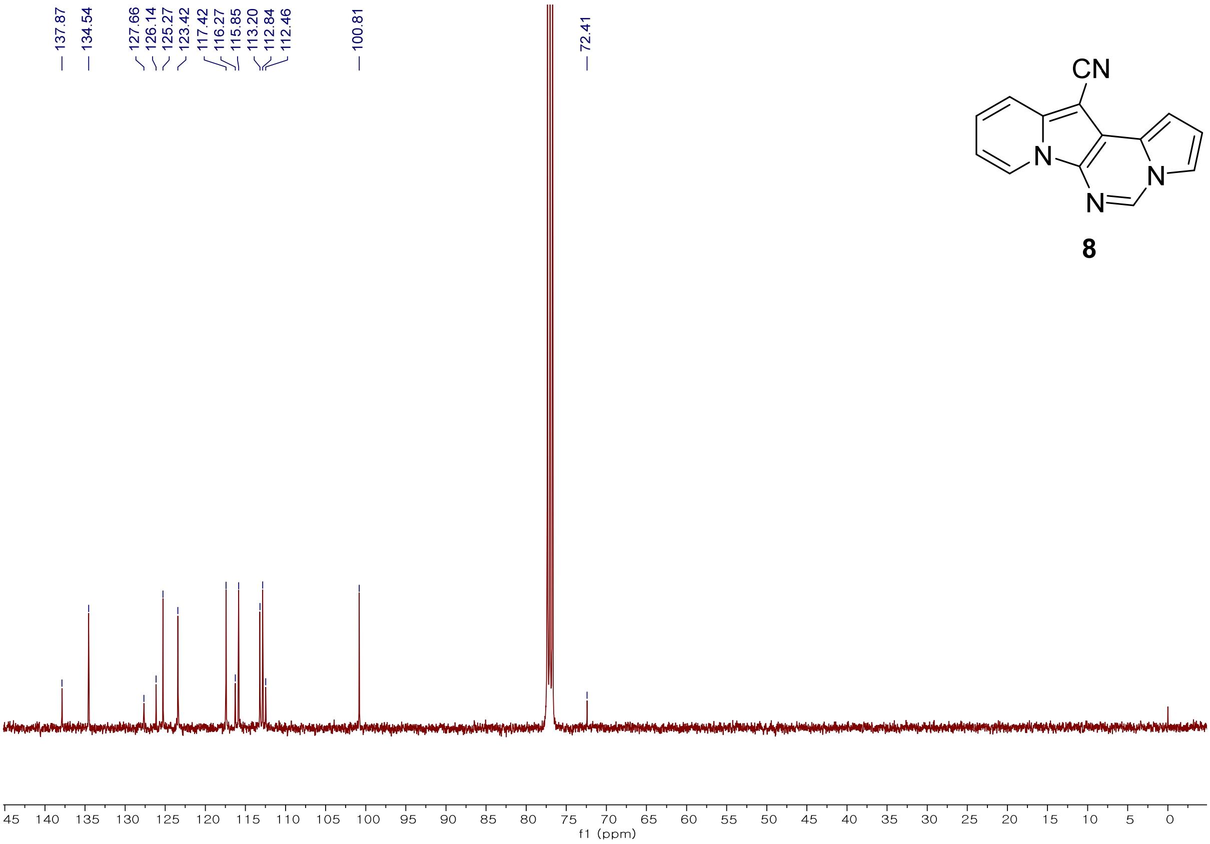
137.87
134.54
127.66
126.14
125.27
123.42
117.42
116.27
115.85
113.20
112.84
112.46
100.81
72.41
CN
N
N
N
f1 (ppm)

8

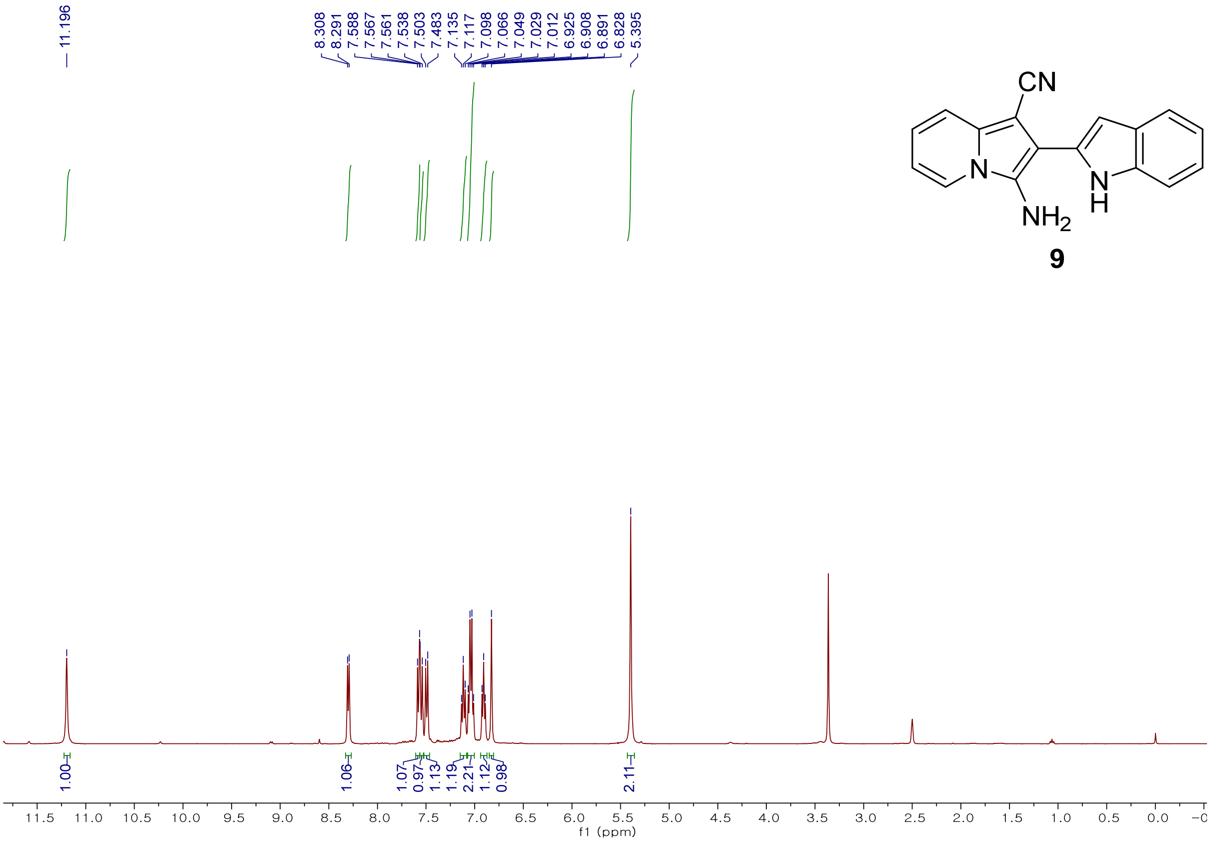
11.196
8.308
8.291
7.588
7.567
7.561
7.538
7.503
7.483
7.135
7.117
7.098
7.066
7.049
7.029
7.012
6.925
6.908
6.891
6.828
5.395
CN
N
NH2
N
H
9
1.00
1.06
1.07
0.97
1.13
1.19
2.21
1.12
0.98
2.11
f1 (ppm)

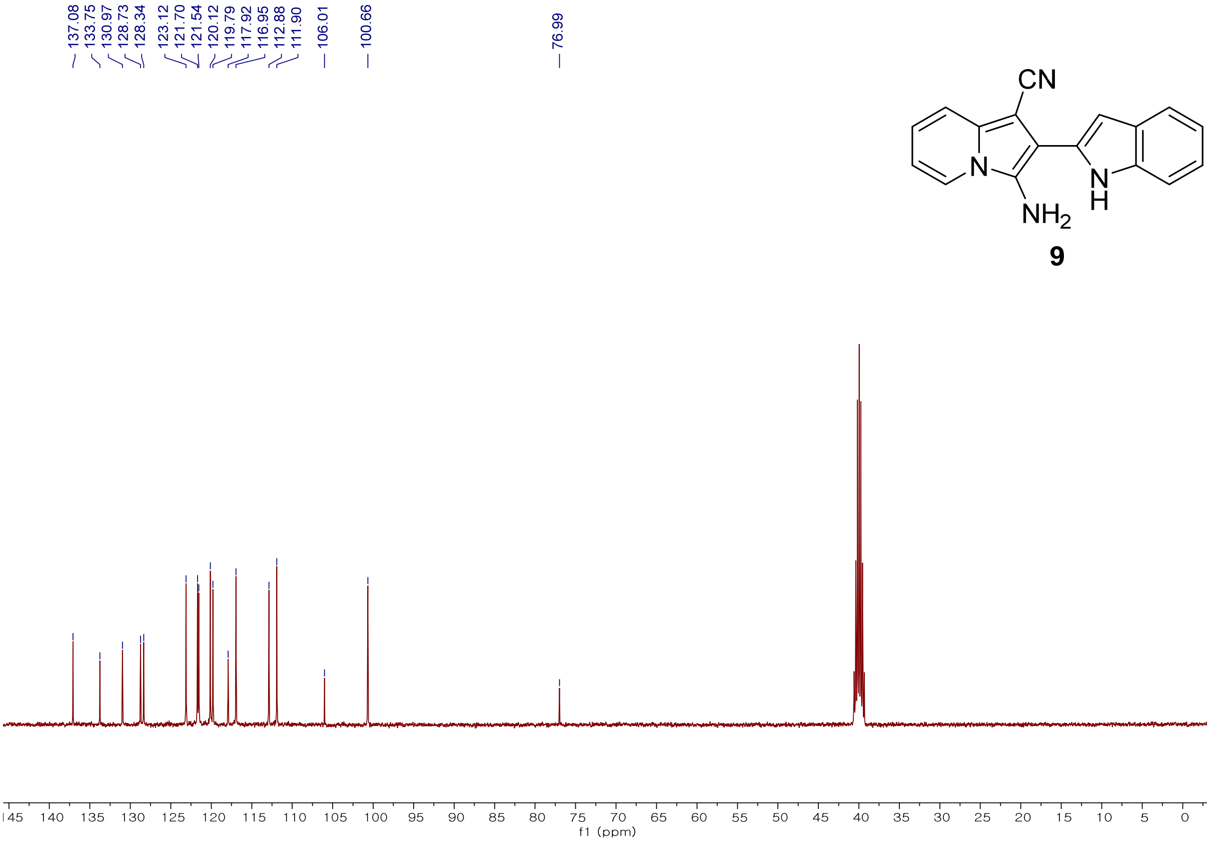
137.08
133.75
130.97
128.73
128.34
123.12
121.70
121.54
120.12
119.79
117.92
116.95
112.88
111.90
106.01
100.66
76.99
CN
N
NH2
N
H
9
f1 (ppm)

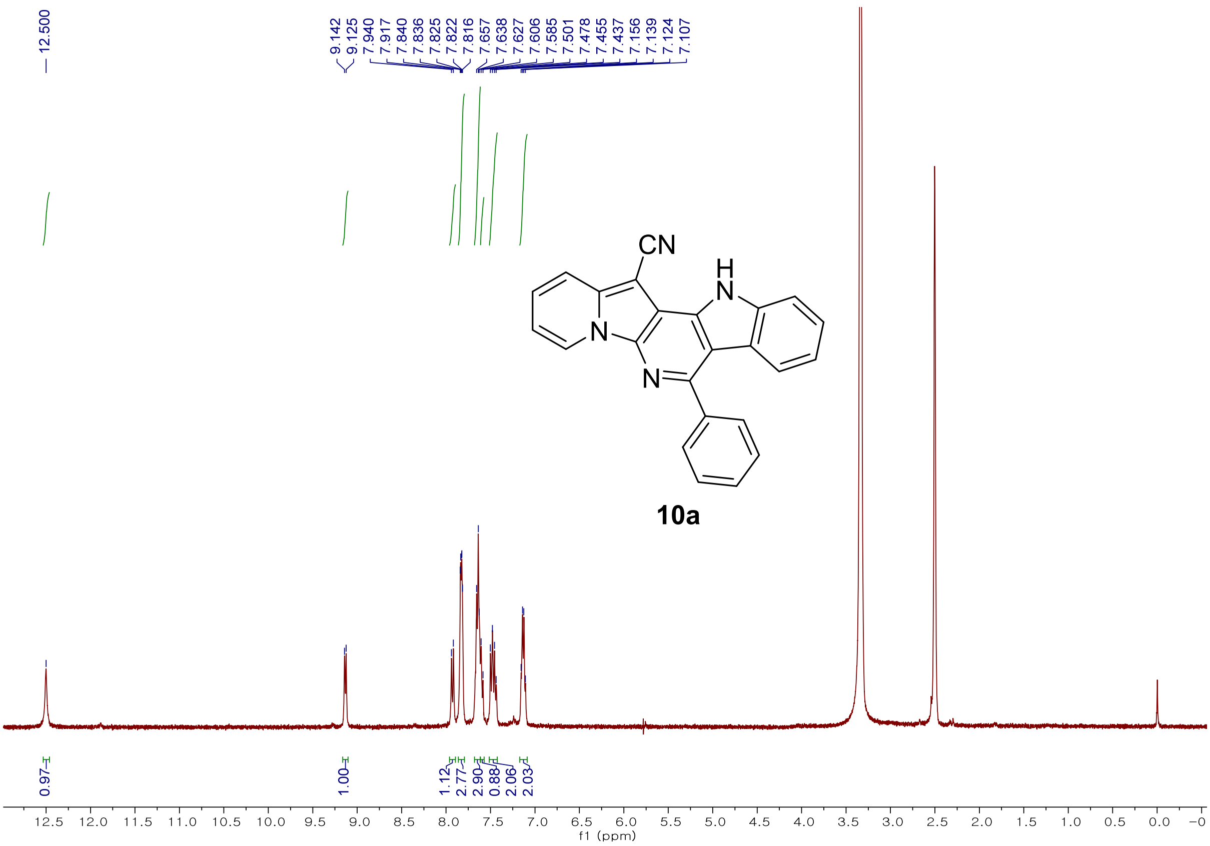
12.500
9.142
9.125
7.940
7.917
7.840
7.836
7.825
7.822
7.816
7.657
7.638
7.627
7.606
7.585
7.501
7.478
7.455
7.437
7.156
7.139
7.124
7.107
CN
H
N
N
N
10a
0.97
1.00
1.12
2.77
2.90
0.88
2.06
2.03
f1 (ppm)

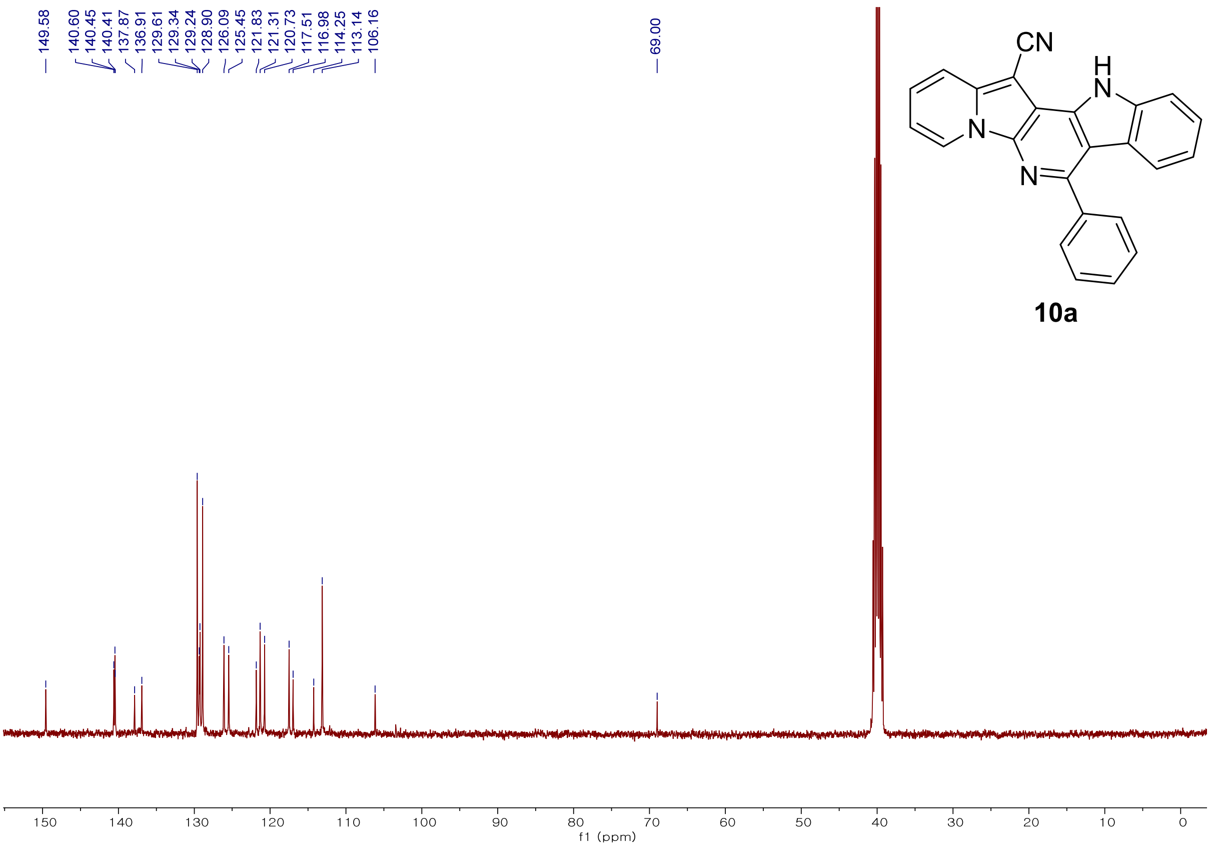
149.58
140.60
140.45
140.41
137.87
136.91
129.61
129.34
129.24
128.90
126.09
125.45
121.83
121.31
120.73
117.51
116.98
114.25
113.14
106.16
69.00
CN
H
N
N
N
10a
f1 (ppm)

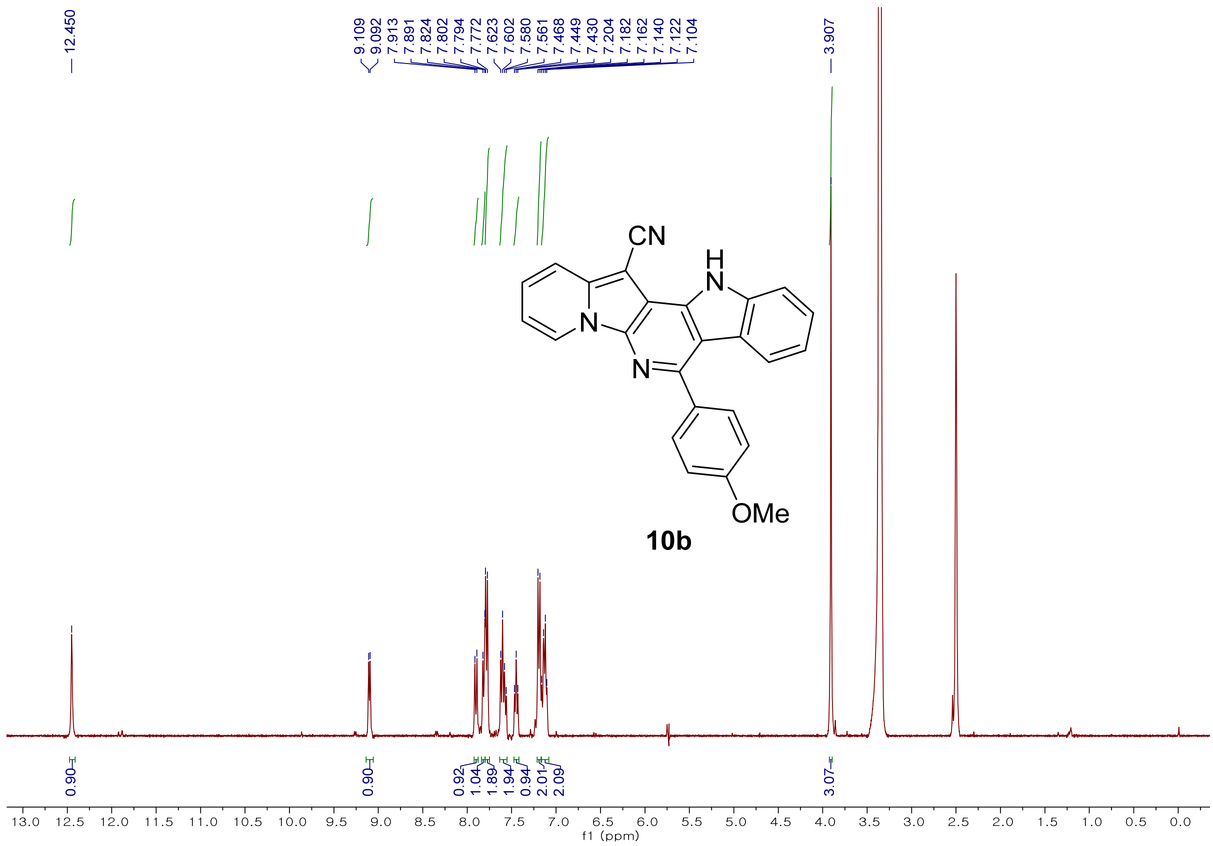
12.450
9.109
9.092
7.913
7.891
7.824
7.802
7.794
7.772
7.623
7.602
7.580
7.561
7.468
7.449
7.430
7.204
7.182
7.162
7.140
7.122
7.104
3.907
CN
H
N
N
N
OMe
10b
0.90
0.90
0.92
1.04
1.89
1.94
0.94
2.01
2.09
3.07
f1 (ppm)

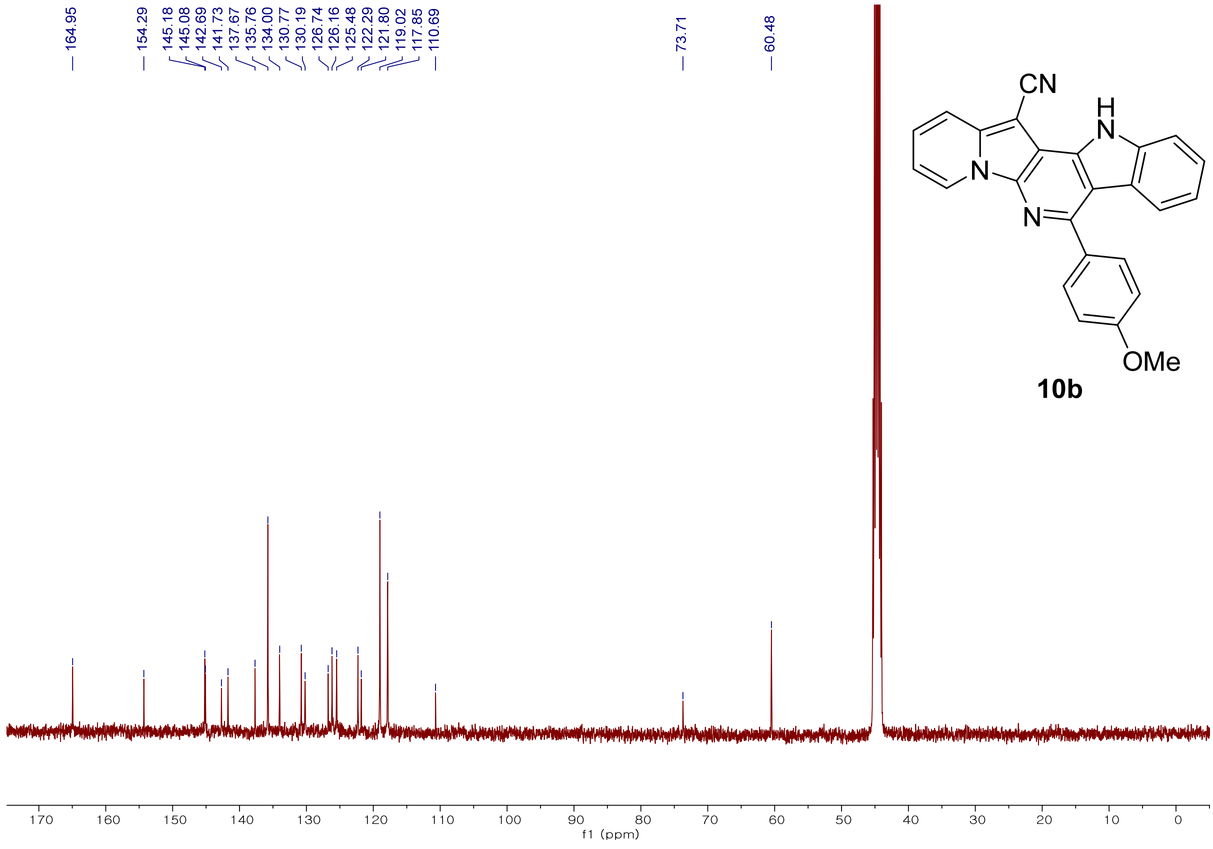
164.95
154.29
145.18
145.08
142.69
141.73
137.67
135.76
134.00
130.77
130.19
126.74
126.16
125.48
122.29
121.80
119.02
117.85
110.69
73.71
60.48
CN
H
N
N
N
OMe
10b
f1 (ppm)

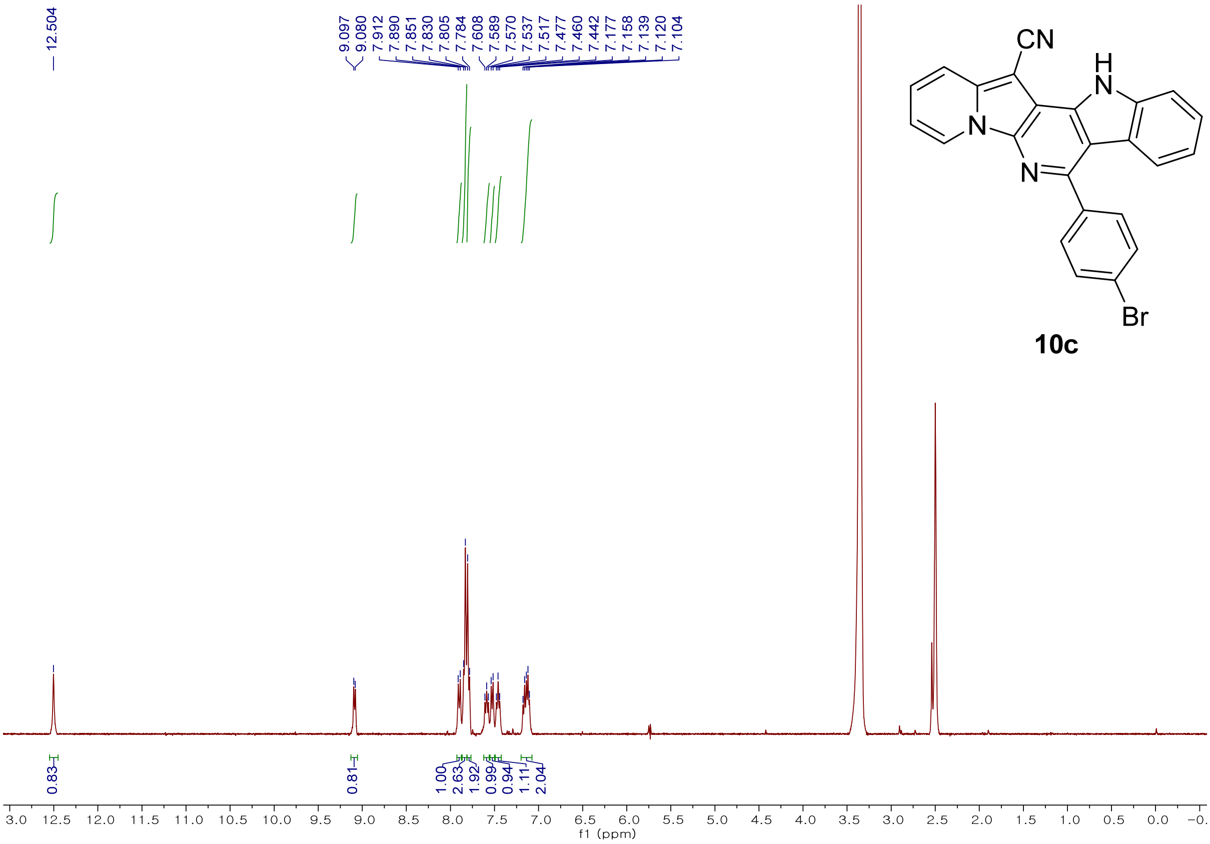
12.504
9.097
9.080
7.912
7.890
7.851
7.830
7.805
7.784
7.608
7.589
7.570
7.537
7.517
7.477
7.460
7.442
7.177
7.158
7.139
7.120
7.104
CN
H
N
N
N
Br
0.83
0.81
1.00
2.63
1.92
0.99
0.94
1.11
2.04
f1 (ppm)

10c

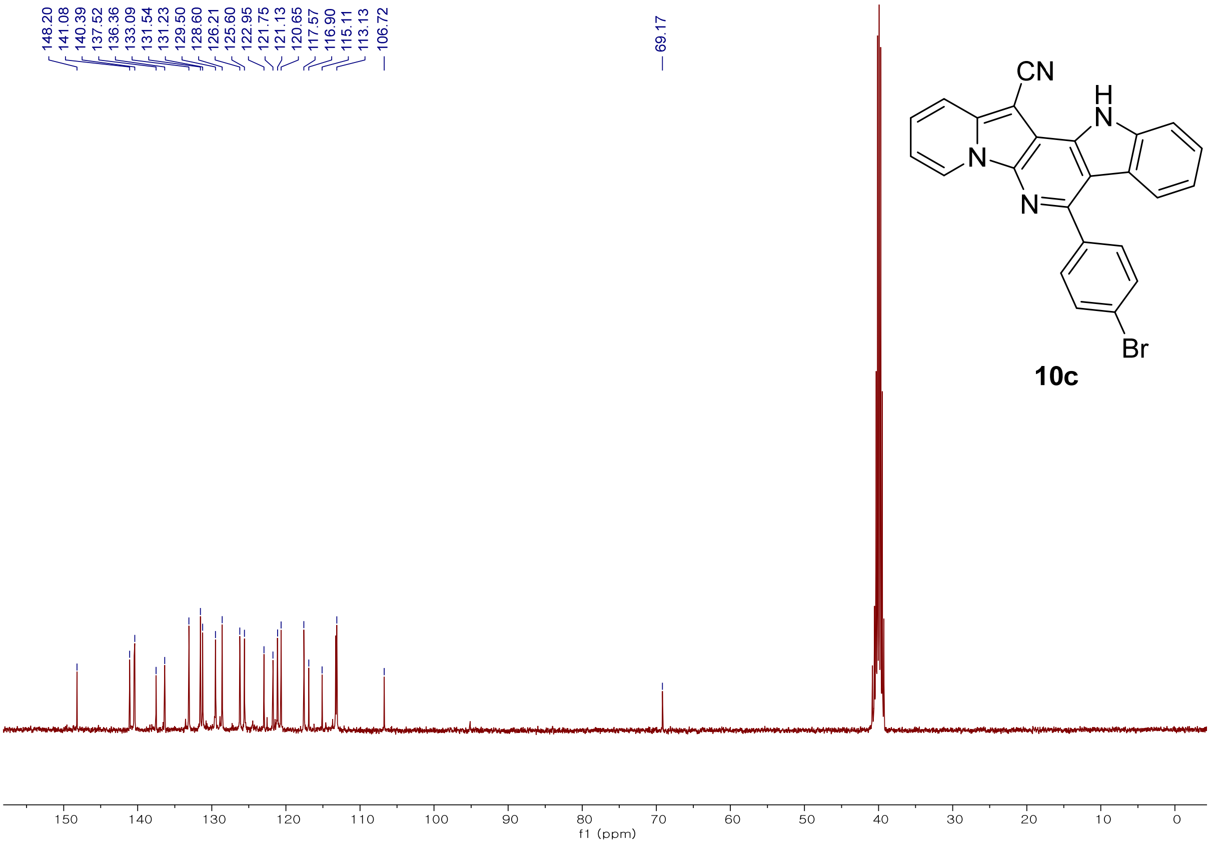
148.20
141.08
140.39
137.52
136.36
133.09
131.54
131.23
129.50
128.60
126.21
125.60
122.95
121.75
121.13
120.65
117.57
116.90
115.11
113.13
106.72
69.17
CN
H
N
N
N
Br
f1 (ppm)

10c

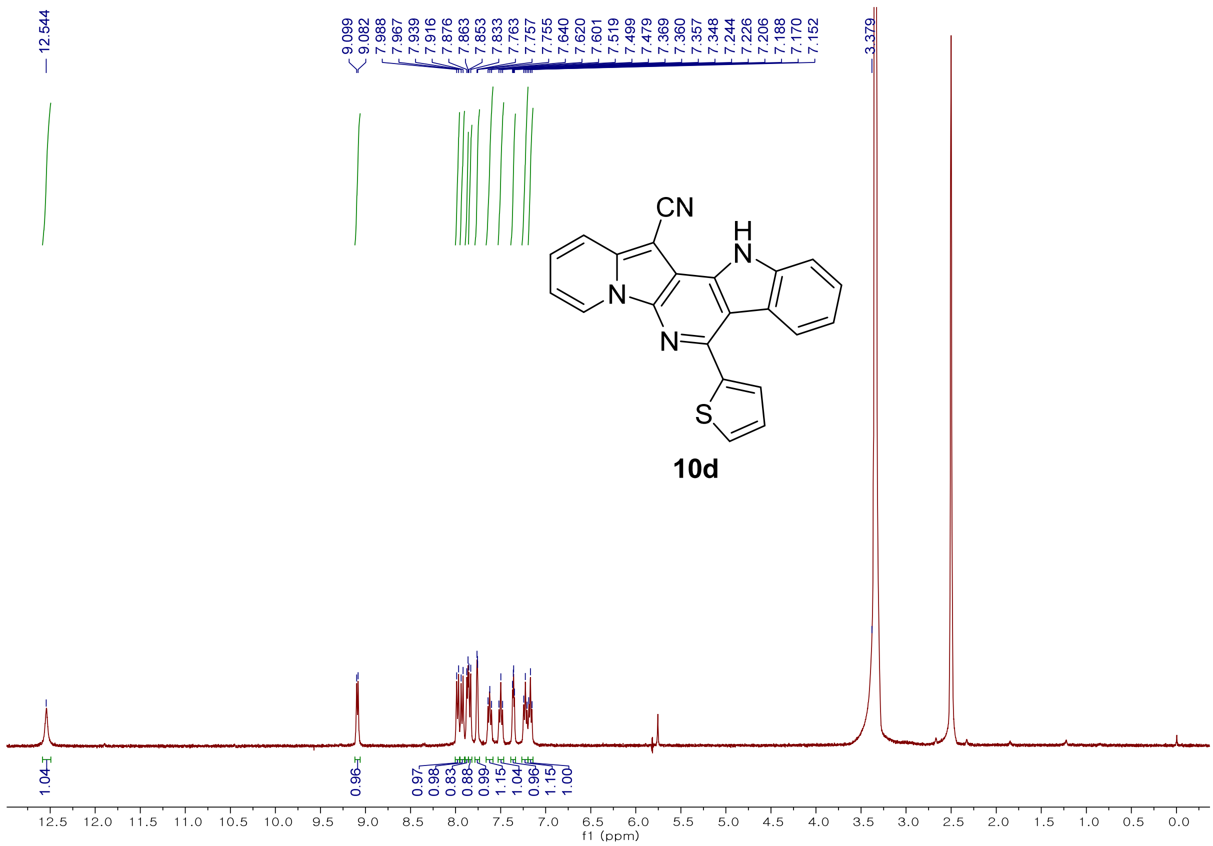

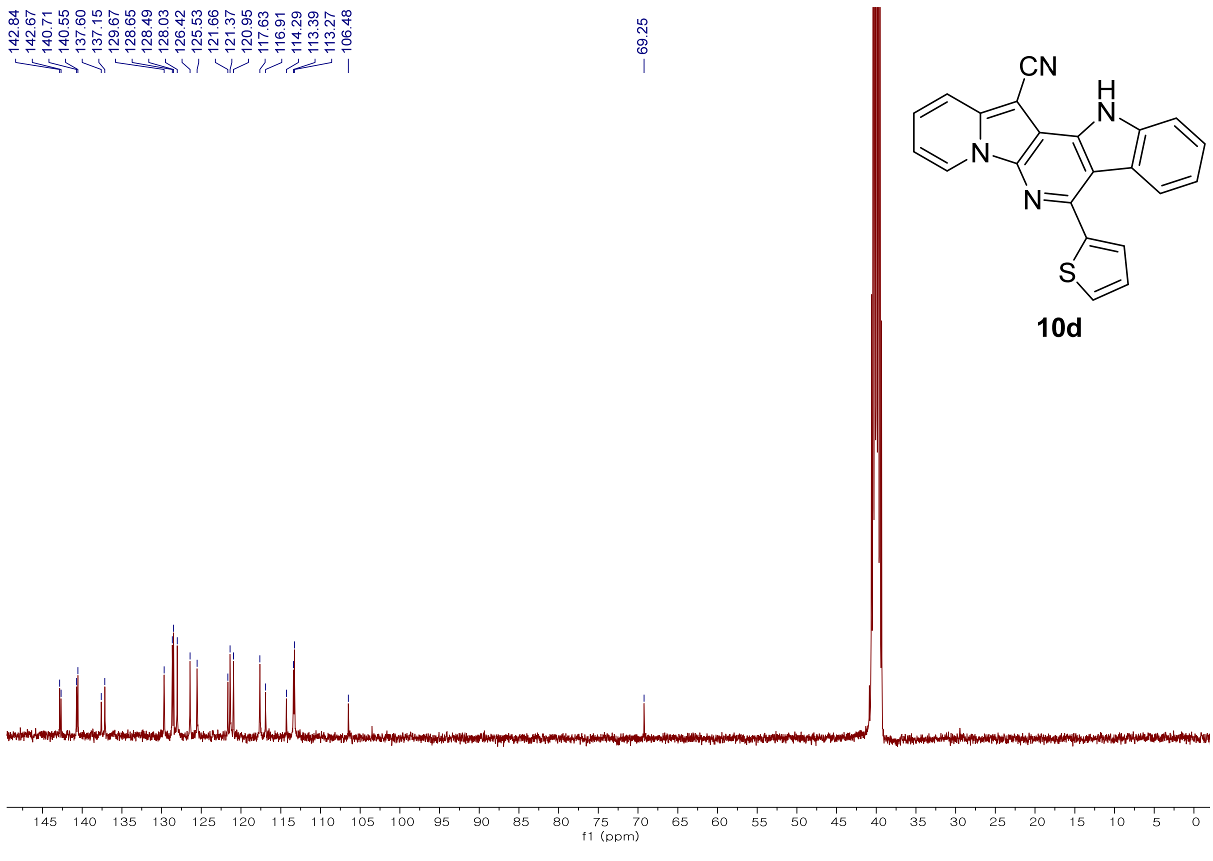

Supplement: Supplementary file 1 [file pharmaceuticals-15-01395-s001.zip › pharmaceuticals-1975836-supplementary.pdf]
